# Taxonomy and ecology of genus *Psyra* Walker, 1860 (Lepidoptera: Geometridae: Ennominae) from Indian Himalaya

**DOI:** 10.1371/journal.pone.0266100

**Published:** 2022-04-13

**Authors:** Kaushik Mallick, Rushati Dey, Uttaran Bandyopadhyay, Arna Mazumder, Subrata Gayen, Mohd Ali, Gaurab Nandi Das, Angshuman Raha, Abesh Kumar Sanyal, Sandeep Kumar Gupta, Virendra Prasad Uniyal, Kailash Chandra, Vikas Kumar

**Affiliations:** 1 Zoological Survey of India, New Alipore, Kolkata, West Bengal, India; 2 Wildlife Institute of India, Dehradun, Uttarakhand, India; Sichuan University, CHINA

## Abstract

The *Psyra* Walker, 1860, is a typical Sino-Himalayan genus of the subfamily Ennominae, currently known by 18 species/4 subspecies globally and 9 species from India. This study aims to revise the taxonomy and ecology of Indian *Psyra* by providing a morphology-based diagnostic key, highlighting their altitudinal, habitat and seasonal preferences, and modelling their distribution based on current and future climatic scenarios. Here, we describe a new species, *P*. *variabilis* **sp. nov.** and document 4 species and 1 subspecies as new to India, viz. *P*. *gracilis*, *P*. *szetschwana*, *P*. *dsagara*, *P*. *falcipennis* and *P*. *debilis debilis*, thus updating the global species count to 19 with 14 species/1 subspecies from India. We also submitted partial mitochondrial *COI* sequences of *P*. *crypta*, *P*. *similaria*, *P*. *spurcataria* and *P*. *gracilis* as novel to the global genetic database and calculated the overall genetic divergence was 5.17% within the genus, suggesting strong monophyly. Being a typical montane genus, most of the species of *Psyra* were active within 2000–2280 m altitude, 10.55–15.7°C annual mean temperature, 1200–2300 mm annual precipitation and 168–179 NDVI. *Psyra* species were predominant in wet temperate, mixed coniferous and moist temperate deciduous forests, their abundance and richness being at peak during post-monsoon months of October–November. The major bioclimatic variables influencing the overall distribution of the genus were mean temperature of warmest quarter, temperature seasonality and precipitation of coldest/driest quarter. While two of the modelled species were predicted to lose area occupancy under future climatic scenarios, the narrow-specialist, Trans-Himalayan species *P*. *debilis debilis* was projected to gain up to 75% additional area in the years 2041–60. The results of this study will be helpful to identify sites with maximum area loss projection in ecologically fragile Indian Himalaya and initiating conservation management for such climatically vulnerable insect species groups.

## Introduction

The Ennominae genus *Psyra* was established by Walker [1] with a single species, *Psyra cuneata*, collected by Major Parry from northern India. Subsequently, Walker [2] described a species under *Hyperythra* Guenée, [1858], *H*. *spurcataria*, collected from Darjeeling, West Bengal and presented to the British Museum of Natural History by the Secretary of the Indian Board. Butler transferred this species to genus *Psyra* while cataloguing the Lepidoptera collection by Hocking from Dharamshala and Kangra in Himachal Pradesh. In 1866, Walker [3] described another species *Scotosia angulifera* from Russell’s Dharamshala collection, which was subsequently transferred under genus *Psyra* in 1867 by Moore [4], who mentioned the species from Darjeeling. In the same publication, Moore described a fourth species *P*. *similaria* collected from Darjeeling by Russell and described another species *Hyperythra trilineata*, which was later transferred under genus *Psyra* by Hampson. Warren [5] in 1888, on examination of 3 male specimens collected by Major Yerburry from Thundiani, Pakistan Himalaya described a sixth species, *P*. *debilis* of this enigmatic genus which was till then known only from Indian Himalaya. In the following year, Butler [6] described a new species *Tetrcis indica* from Hocking’s Dharamshala collection and mentioned its affinity with Warren’s *P*. *debilis*, under which the species was later revised as a subspecies by Parsons et al. [7] while cataloguing global Geometridae. Thus, when Hampson wrote *The Fauna of British India*, *Moths Vol*. *III* [8], he mentioned seven species treating *P*. *similaria* as a synonym of *P*. *cuneata*, all distributed in the Himalaya and North-Eastern Hill ranges of Khasi. Moreover, he mentioned the species *P*. *cuneata* from Japan, which was later added to China by Leech in 1897 [9] from Preyer’s collection. The first species of *Psyra* described outside Himalaya was *P*. *boarmiata* (Graeser, 1892) [10] from Amurland, Russia. Later, Leech [9] described *P*. *rufolinearia* from Sichuan, China; Püngeler [11] described *P*. *bluethgeni* from Japan; Bastelberger [12, 13] described three species from Taiwan, among which *P*. *matsumurai* is currently treated as a subspecies of *P*. *cuneata*, while, *P*. *ferruginea* and *P*. *florida* are presently treated as junior subjective synonyms of *P*. *spurcataria*. Three new subspecies of *P*. *cuneata* were described by Wehrli [14] from China, two of them, *P*. *szetschwana* and *P*. *dsagara* had recently been upgraded to species status and the third, *P*. *lidjangica* had been downgraded as a synonym of *P*. *szetschwana* by Liu et al. [15]. Between 1954 to 1983, Inoue [16–20] described four new taxa under this genus: two subspecies of *P*. *boarmiata*, *P*. *b*. *subcuneata* and *P*. *b*. *massuii* from Japan, along with *P*. *conferta* from Taiwan and *P*. *moderata* from Nepal, the latter being recently added to the Chinese fauna by Liu et al. [15]. Yazaki [21, 22] established four new species from Nepal and India: *P*. *gracillis* from Godavari, Nepal; *P*. *fulvaria* from Sikkim, India; *P*. *falcipennis* and *P*. *crypta* from Mt. Phulchouki, Nepal, the last one added to the Indian fauna by Sanyal et al. [23] from Uttarakhand. The most recent addition to the genus, *P*. *breviprotrusa* was made by Liu et al. [15] from Gansu, China, along with the first most comprehensive genus revision on Chinese fauna. Thus, 18 species and 4 subspecies were till now known from the world, majority of which being described and reported from Sino-Himalayan Region: 13 species and 3 subspecies from China, 9 species from India, 8 species from Nepal and 4 species from Taiwan. Outside this area, only 4 species are reported from the Mandschuric region: 3 species from Japan and 1 species from Russia. Except for the recent revisionary work on Chinese fauna [15], the genus has not been properly revised within the Himalayan region. Moreover, little information exists regarding their ecology including habitat, altitudinal and seasonal preferences and biotic-abiotic determinants shaping their diversity and distribution patterns. Larval ecology of the genus is very poorly known except that some of the species are probably polyphagous feeding upon various families of trees and herbs like Saxifragaceae, Lauraceae, Rosaceae, Polygonaceae, Fagaceae etc. [24], documented mostly from Japan, especially for the species *P*. *bluethgeni*. Smetacek & Smetacek [25] reported genus *Rosa* (Rosaceae) as a larval food plant of *P*. *spurcataria* from Nainital district, Uttarakhand.

Confusion about the taxonomy of the genus *Psyra* exists mainly regarding their Tribal placement, as the genus has characteristics of both Boarmiini and Gnophini, thus existing in a transitional position between them [15]. *Psyra* possesses the Boarmiini character by the presence of a setal comb on the third abdominal sternite as well as Gnophini characters by the presence of a costal projection and an apical spine on the valvae in male genitalia [26]. A recent comprehensive phylogenetic analysis covering major Boarmiini genera revealed that *Psyra* along with *Loxaspilates*, *Menophra*, *Hirasa*, *Charissa* and *Phthonandria* formed a separate clade under tribe Gnophini [27], though the ambiguity regarding the systematic position of Gnophini is still far from resolved.

In an attempt to revise the taxonomic account of Indian/Himalayan *Psyra*, we have provided here a morphology-based key with diagnosis and illustrations of external features and genitalia for species identification. With the description of one new species and addition of 4 species and a subspecies to the country, we have updated the Indian count to 14 species/1 subspecies and the global number to 19 species/4 subspecies. We have submitted the partial mitochondrial Cytochrome C Oxidase I (*COI*) sequences of 4 morphologically identified species to the National Centre for Biotechnology Information (NCBI) and Barcode of Life Database (BOLD). Additionally, we sought to outline the crucial ecological characteristics of the genus and individual species, highlighting their climatic and habitat specificity, seasonal abundance and biotic-abiotic parameters governing their distribution. We have investigated the habitat-suitability of genus *Psyra* along with two selected species under current climatic conditions and predicted their future distributions under various carbon emission scenarios.

## Material and methods

### Field sampling & identification

**Fig 1:**
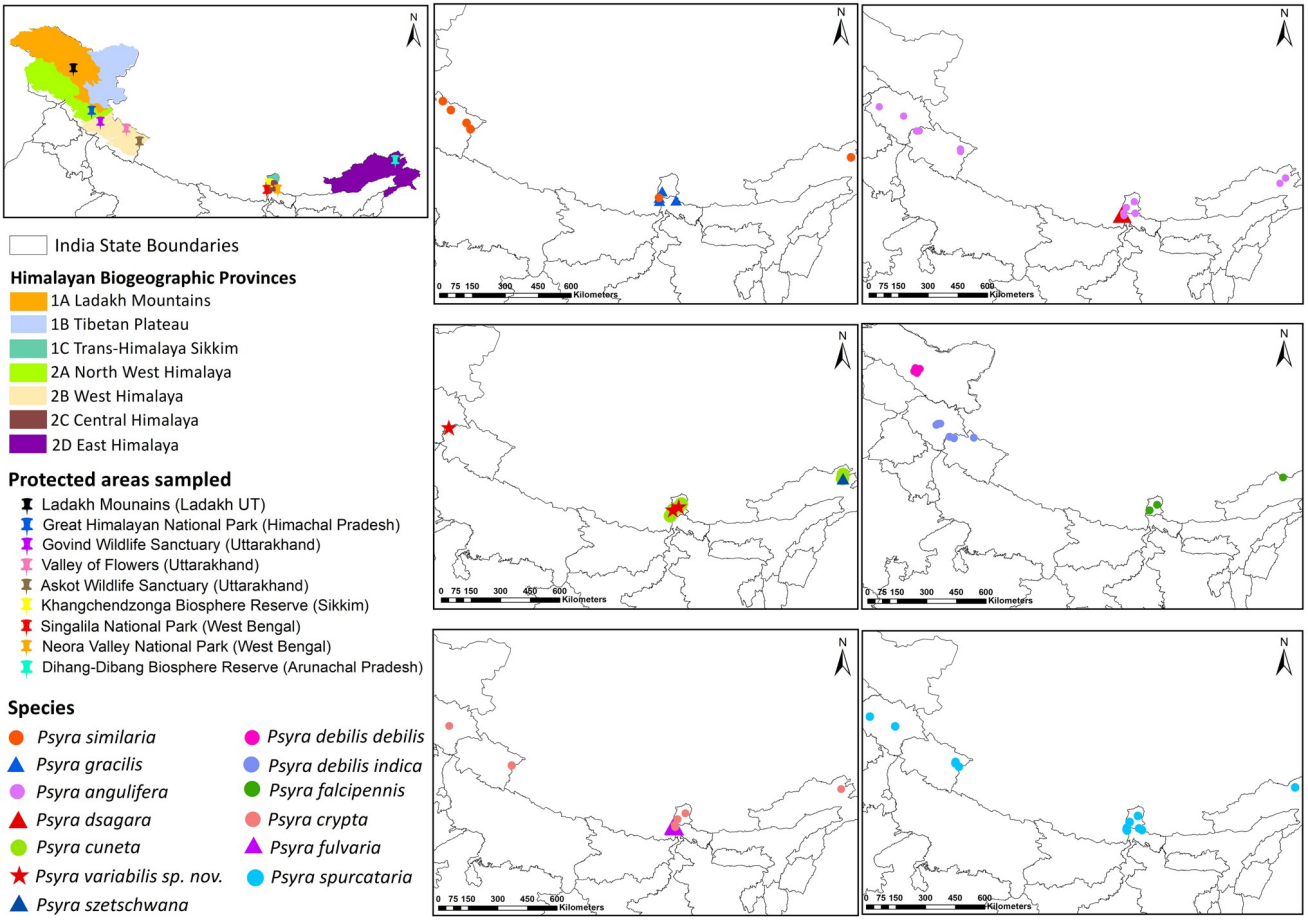
Collection localities of *Psyra* species/subspecies recorded from 5 biogeographic provinces and 9 Protected Areas in the Indian Himalaya during sampling period of 2010–2012 and 2016–2019.

Sampling was conducted in 9 Protected Areas within 5 major biogeographic provinces of the Indian Himalaya [28], viz. Ladakh, Trans-Himalaya (1A); Dharamshala and Great Himalayan National Park in Himachal Pradesh, North-Western Himalaya (2A); Govind Wildlife Sanctuary, Valley of Flowers National Park and Askot Wildlife Sanctuary in Uttarakhand, Western Himalaya (2B); Khangchendzonga Biosphere Reserve in Sikkim, Neora Valley National Park and Singalila National Park in West Bengal, Central Himalaya (2C); Dihang Dibang Biosphere Reserve in Arunachal Pradesh, Eastern Himalaya (2D) (Fig 1). Sampling permission in these Protected Areas were obtained from respective State Principal Chief Conservator of Forest offices and all the sampling was done with prior approval from respective Divisional Forest offices. Stratified random sampling framework was adapted to sample sites at every 200 m in pre-selected altitudinal gradients from lowest to the highest elevation within a Protected Area. We categorized all sites into forest/habitat types based on altitude and dominant plant species following Champion & Seth [29]. Sampling was carried out in 2010–2012 and 2016–2019, covering 3 broad seasons predominant in Himalaya, viz., pre-monsoon (April–June), monsoon (July–September) and post-monsoon (October–December). We collected specimens in front of a white-sheet that was reflecting light from artificial light-sources. In remote areas, we used a combination of solar-powered Light-Emitting-Diode (LED) lamp of 48 W with 32 bulbs (1.5 W each) and pressurized-paraffin lamp (petromax) of 80 W with white incandescent light, whereas Mercury Vapour (MV) lamp of 160 W was used wherever electricity was available. Garmin Oregon 550 GPS was used to record exact geographical coordinates and altitude. Temperature and relative humidity at the trap site were recorded using Kestrel 3000 Weather Meter. Moths attracted to the light trap were first photographed and then collected in a glass bottle with Ethyl Acetate vapour. We curated the collected specimens following standard protocols [30] and deposited voucher specimens in the National Zoological Collection of the Zoological Survey of India, Kolkata. For studying genitalia, abdomen from both male and female specimens were removed and digested in 10% Potassium Hydroxide solution overnight. The soaked abdominal segments were then dissected in 20% Ethyl Alcohol and studied under Leica S8AP0 HD binocular microscope and later stored in vials containing 1:3 ratio of Glycerol and Ethyl Alcohol. For individual species bionomy, data were compiled against both primary, i.e., sampling locations, and geo-referenced secondary locations extracted from existing literature.

### Nomenclatural acts

The electronic edition of this article conforms to the requirements of the amended International Code of Zoological Nomenclature, and hence the new names contained herein are available under that Code from the electronic edition of this article. This published work and the nomenclatural acts it contains have been registered in ZooBank, the online registration system for the ICZN. The ZooBank LSIDs (Life Science Identifiers) can be resolved and the associated information viewed through any standard web browser by appending the LSID to the prefix "http://zoobank.org/". The LSID for this publication is: urn:lsid:zoobank.org:pub:2CC484D3-7671-49AC-8E9D-846CCC38FCAB. The electronic edition of this work was published in a journal with an ISSN, and has been archived and is available from the following digital repositories: PubMed Central, LOCKSS.

### DNA barcoding

Two or three leg samples were removed from the morphologically identified specimens with sanitized forceps and stored in molecular grade 70% Ethanol at 4°C. The standard protocol of Phenol Chloroform-Isoamyl alcohol [31] was used to extract genomic DNA from the legs. The primer pair, LepF1: 5’-ATTCAACCAATCATAAAGATATTGG-3’ and LepR1: 5’-TAAACTTCTGGATGTCCAAAAAATCA-3’ [32] was used to amplify the 648 bp barcode region (*COI*) of the mitochondrial DNA. The total volume of PCR reaction was 30 μl containing: 20 picomoles of each primer, 20 mM Tris-HCl (pH 8.0), 100 mM KCl, 0.1 mM EDTA,1 mM DTT, 1.8 mM MgCl_2_, 0.25 mM of each dNTP and 1 μl of Taq polymerase (Takara Bio Inc., Shiga, Japan). The Veriti VR Thermal Cycler (Applied Biosystems, Foster City, CA) was used for the amplification with the following thermocycling profile: first cycle of 5 min at 94°C, followed by 5 cycles of 1 min at 94°C, 1 min 30 sec at 45°C, 1 min 30 sec at 72°C; followed by 30 cycles of 1 min at 94°C, 1 min 30 sec at 51°C, 1 min 30 sec at 72°C, and final extension for 5 min at 72°C. QIAquick Gel Extraction Kit (Qiagen Inc., Germantown, MD) was used to purify the PCR products. The cycle sequencing of the purified PCR products was performed with BigDye^®^ Terminator ver. 3.1 Cycle Sequencing Kit (Applied Biosystems Inc., California, USA) and finally sequenced using 48 capillary ABI 3730 Genetic analyzer in ZSI, Kolkata. Eight DNA sequences of 4 morphologically identified species of *Psyra* were generated, aligned against 4 available sequences of *Psyra* retrieved from NCBI and BOLD. As an outgroup, *Cleora fraterna* Moore, [33] (NCBI Accession no. KX861428) was used. In the final dataset, 13 sequences were aligned using Clustal X [34] and, 574 bp of *COI* were opted to estimate the genetic divergence and phylogeny. Kimura-2-parameter model was used to calculate the evolutionary genetic divergences and neighbour-joining (NJ) phylogenetic tree was constructed in the software MEGA X with 1,000 bootstraps of replications [35].

### Ecological analysis

#### Habitat preference

Finer level subcategories of forest types were grouped into broad habitat types according to their altitudinal range and biogeographic provinces. Normalized Difference Vegetation Index (NDVI) values against all the point locations were extracted for particular sampling date using NASA LPDAAC Collection’s MOD13Q1 v006 downloaded from USGS Earth Explorer [36], converted into 0–200 scale and averaged for each habitat type. *Psyra* abundance (converted into relative abundance (%) from total abundance recorded per sampling event) was compared among different habitat types and respective NDVI range through Exploratory Data Analysis.

#### Distribution modelling

We used both primary, i.e., sampling locations, and geo-referenced secondary locations compiled from existing literature along with the spatial environmental variables in the presence-only MaxEnt software (Ver: 3.4.1) to predict and quantify the current suitable habitat for the genus *Psyra* and to develop a futuristic habitat distribution model for the selected species. We chose one widely distributed species with the highest number of unique occurrence locations (*P*. *angulifera*) and two range-restricted taxa (*P*. *debilis debilis* and *P*. *debilis indica*) to predict their habitat changes during the years 2041–2060. For the occurrence locations to be spatially independent [37], the “spatially rarefy occurrence data” tool of SDM Toolbox v2.4 [38] was used to remove spatially auto-correlated points at a resolution of 2 km (reducing occurrence localities to a single point within 2 km) using ArcMap 10.4. Nineteen bioclimatic variables obtained from the WorldClim database Version 2.1 [39] representing climate features (averaged over 1970–2000) were used at two resolutions: 30 arc seconds for genus model and 2.5 arc minute for species model. Following CMIP6, the future projections of the *Psyra* species were based on two carbon emission scenarios of Shared Socioeconomic Pathways (SSPs): SSP2-4.5, defined as the moderate optimistic scenario where emissions continue to increase through the end of the century, with resulting warming of 3.8–4.2°C; and SSP5-8.5, the pessimistic “worst-case” scenario to have resultant warming of 4.7–5.1°C (www.carbonbrief.org). The future bioclimatic variables of respective scenarios were obtained from WorldClim for the years 2041–2060 at a resolution of 2.5 arc minutes under the General Circulation Models (GCMs) of BCC-CSM2-MR (The Beijing Climate Center Climate System Model). Values for all the climatic variables were extracted against each unique locality and Principal Component Analysis (PCA) was used to remove auto-correlated variables (correlation coefficient > 0.9). Finally, the non-correlated variables along with Digital Elevation Model (DEM) (USGS Landsat data; United States Geological Survey 2017) were used for the analysis. All the climatic and topographical variables thus used were cropped according to the species-specific biogeographical unit.

All the models were generated with a maximum of 5000 iterations and 10000 background points. For the genus and *P*. *angulifera*, 25 replicated models were generated by subsample method (25% test percentage) using MaxEnt logistic output format, while cross-validate and cloglog functions [40] were used for *P*. *debilis debilis* and *P*. *debilis indica*. Model validation was performed through threshold-independent evaluation using Receiver Operating Characteristics (ROC) from Area under ROC Curve (AUC) values ranging from 0 to 1 [40]. To calibrate and validate the robustness of results for the MaxEnt model, threshold-dependent True Skill Statistics (TSS) score was also calculated. The final potential species distribution map had a range of values from 0 to 1, which were regrouped into four classes of habitat suitability: unsuitable (0–0.25), low (0.25–0.5), moderate (0.5–0.75) and high (0.75–1). All the related GIS works were performed using ArcMap 10.4. For estimating the niche overlap between the two subspecies of *P*. *debilis*, Schoener’s D [41] was calculated using the software ENM Tools v1.3 [42].

#### Influence of major environmental predictors and seasonal abundance

Major bioclimatic variables like annual precipitation, precipitation of driest month, mean temperature of warmest quarter, precipitation of coldest quarter governing genus distribution and crucial environmental factors like altitude, NDVI, trap night temperature, relative humidity were used to explore their comparative influence on the species abundance pattern in different Himalayan biogeographic provinces through Canonical Correspondence Analysis (CCA) in the programme PAST (Version 2.17c). Total monthly encounters representing the seasonal abundance pattern was compared across trap night temperature and relative humidity gradients using MS Excel 2019.

## Results

### Systematic account

#### *Psyra* Walker, 1860

*Psyra* Walker [1]: 311, 482. Type species: *Psyra cuneata* Walker, 1860, by monotypy.

*Orbasia* Swinhoe [43]: 222. Type species: *Hyperythra spurcataria* Walker, 1863, by monotypy.

*Oncodocnemis* Rebel [44]: 354. Type species: *Phasiane boarmiata* Graeser, 1892, by monotypy.

#### Genus description

Head: Proboscis long, slender and coiled; antennae filiform in both sexes, in males slightly thicker and flatter; frons round; labial palpi short, upturned, hairy, crossing beyond frons, but not reaching vertex, third joint extremely small and concealed.

**Fig 2:**
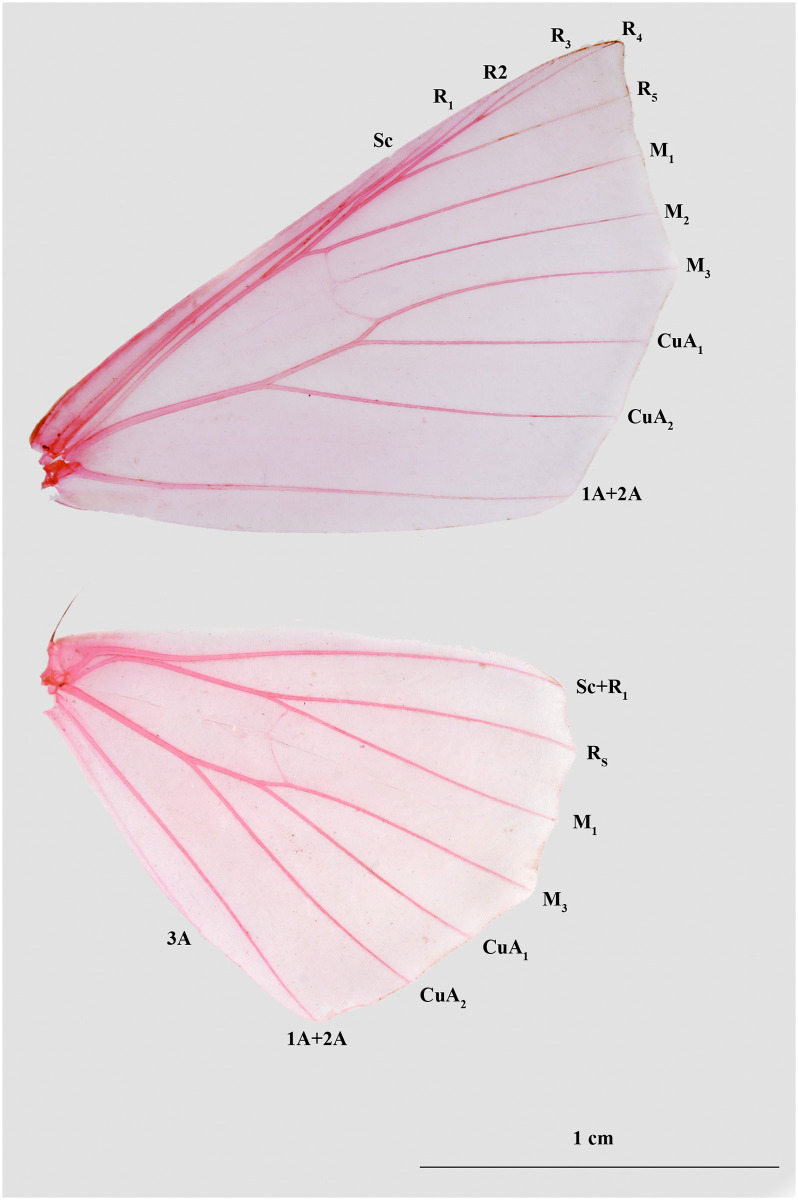
Forewing and hindwing venation of genus *Psyra* (of *P*. *spurcataria*).

Thorax: Metathorax with a pair of prominent specks; hind tibia much dilated, especially in males, with two pairs of stout spines; wings of various shades of straw colour, from golden-ochreous to much browner shades, suffused and irrorated with fuscous; forewing mostly subfalcate with acute apex, outer margin angled at M_3_; SC just touches R_1_ for a very short distance, R_1_ branches off from the common stalk of R-M_1_ just before touching SC; R_2_ anastomoses with R_1_ for a short distance, but with the distinct origin; M_1_ separates from R_3_-R_5_ before cell; M_3_ originates directly from the angle of the cell; CuA_1_ distinct from M_3_ and originates before the angle of cell (Fig 2). By forewing appearance, two distinct groups of species can be recognized. Indian group of species *P*. *angulifera*, *P*. *cuneata*, *P*. *similaria*, *P*. *gracilis*, *P*. *szetschwana*, *P*. *fulvaria* and *P*. *dsagara* can be defined by the presence of black, triangular or wedge-shaped patches of varying size, with the most prominent one being on the submarginal line between CuA_2_ and 1A+2A. The other group consisting of *P*. *debilis*, *P*. *crypta*, *P*. *falcipennis* and *P*. *spurcataria* lacks any such prominent black, wedge-shaped patches in forewing; hindwing with the outer margin slightly angulated at R_S_ and M_3_; R_S_ and M_1_ separate off before cell; CuA_1_ separates from M_3_ and originates before the angle of cell; 3A distinct; species vary in the pattern of the medial band, which are sometimes broken into distinct lines.

Male Genitalia: Uncus more or less triangular with a broad base and tapering apex, sometimes with a lesser degree of sclerotization; gnathos mostly tongue-like with broad apex; valvae mostly triangular, broad, short to scimitar-shaped with curved apex; costal basal process prominent, long, mostly straight, sometimes curved outward and reaching almost the length of the uncus and mostly ends in hook-like tips; saccus mostly U-shaped to triangular with medial protrusion; juxta moderately sclerotized; aedeagus short to moderately long with varying number of vesica spines: none to single as in *P*. *crypta*, *P*. *fulvaria*; mostly two as in *P*. *angulifera*, *P*. *similaria*, *P*. *dsagara*, *P*. *szetschwana*; sometimes three as in *P*. *cuneata*, *P*. *gracilis*, *P*. *spurcataria*; cornutus usually thin and spine-like.

Female Genitalia: Papilla analis distinctly oval; ductus bursae sclerotized, wrinkled, with variable length and degree of coiling. Corpus bursae round to irregularly oval with a large, ovoid signum bearing multiple, large marginal spines and few central spines.

#### Identification key to the Indian species of Genus *Psyra*

1. Forewing submarginal area with a large, black, triangular patch between vein 2A and CuA_2_…….**2**

• Forewing submarginal area without large, black, triangular patch between vein 2A and CuA_2_……**8**

2. In hindwing, the medial line is very close to the discal spot.………………………….……….…**3**

• In hindwing, the medial line is far apart from the discal spot.……………………….………….…**7**

3. Black patches on the forewing are distinctly outlined………………………,,……………………**4**

• Black patches on the forewing are not outlined…………………………***P***. ***gracilis* Yazaki, 1992**

4. Black patches on the forewing outlined with golden-brown………***P***. ***angulifera* (Walker, 1866)**

• Black patches on the forewing outlined with pale ochreous…………………………………….…**5**

5. In forewing, discal spots are conjoined………….………………………….…………………….**6**

• In forewing, discal spots are disjoined…………………………………..***P***. ***cuneata* Walker, 1860**

6. Hindwing with double medial band…………………………………***P***. ***szetschwana* Wehrli, 1953**

• Hindwing with single medial band……………………….……………….***P***. ***fulvaria* Yazaki, 1992**

7. In forewing, the last submarginal triangular, black patch touches the inner margin………………… ***P***. ***dsagara* Wehrli, 1953**

• In forewing, the last submarginal triangular, black patch doesn’t touch the inner margin…………. ***P***. ***similaria* Moore, 1867**

8. Forewing with four prominent black costal blotches……………………***P***. ***debilis* Warren, 1888**

• Forewing without any costal blotch………………………………………………………………**9**

9. In forewing, series of three black spots from inner margin up to CuA_2_ just beyond postmedial band…………………………………………………………………………………………………**10**

• Forewing without any black spot from inner margin up to CuA_2_ beyond postmedial band………**11**

10. In forewing, two small, black, triangular spots between M_3_ and M_1_, disconnected from and inside the apical streak…………………………………………………………***P***. ***falcipennis* Yazaki, 1994**

• In forewing, two small, black, triangular spots between M_3_ and M_1_, on the apical streak…………. ***P***. ***variabilis* Mallick, Bandyopadhyay & Sanyal sp. nov.**

11. In hindwing underside, outer postmedial line very prominent, complete and crenulated………. ***P*. *crypta* Yazaki, 1994**

• In hindwing underside, outer postmedial line obsolescent and incomplete…………………….…. ***P***. ***spurcataria* (Walker, 1863)**

#### *Psyra gracilis* Yazaki, 1992

**Fig 3:**
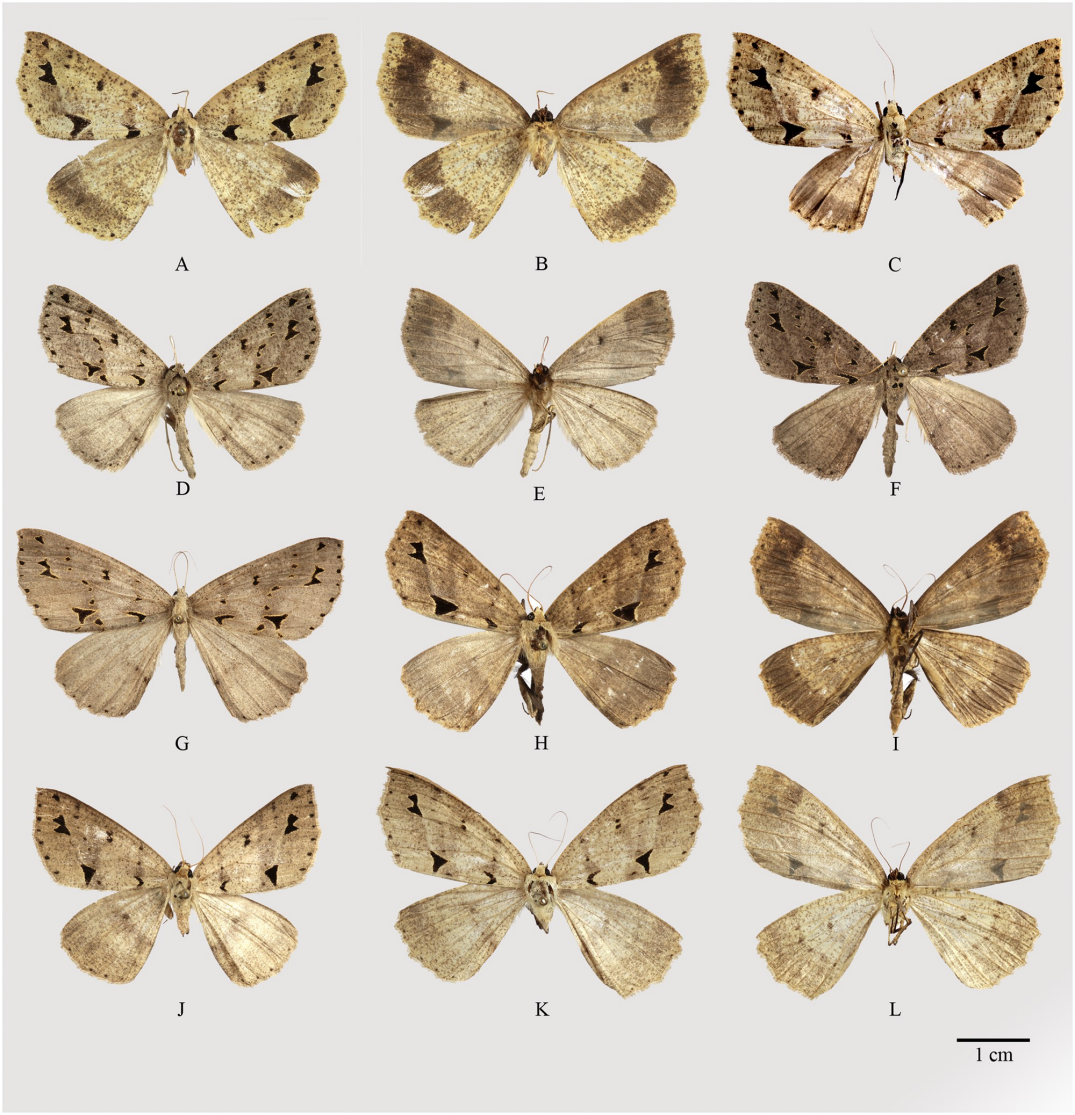
Habitus of *Psyra gracilis*, *P*. *angulifera*, *P*. *cuneata*. A. *P*. *gracilis* ♂ B. *P*. *gracilis* ♂ underside C. *P*. *gracilis* ♀ D. *P*. *angulifera* ♂ E. *P*. *angulifera* ♂ underside F. *P*. *angulifera* ♂ (Dark morph) G. *P*. *angulifera* ♀ H. *P*. *cuneata* ♂ I. *P*. *cuneata* ♂ underside J. *P*. *cuneata* ♂ (Pale morph) K. *P*. *cuneata* ♀ L. *P*. *cuneata* ♀ underside.

**Fig 4:**
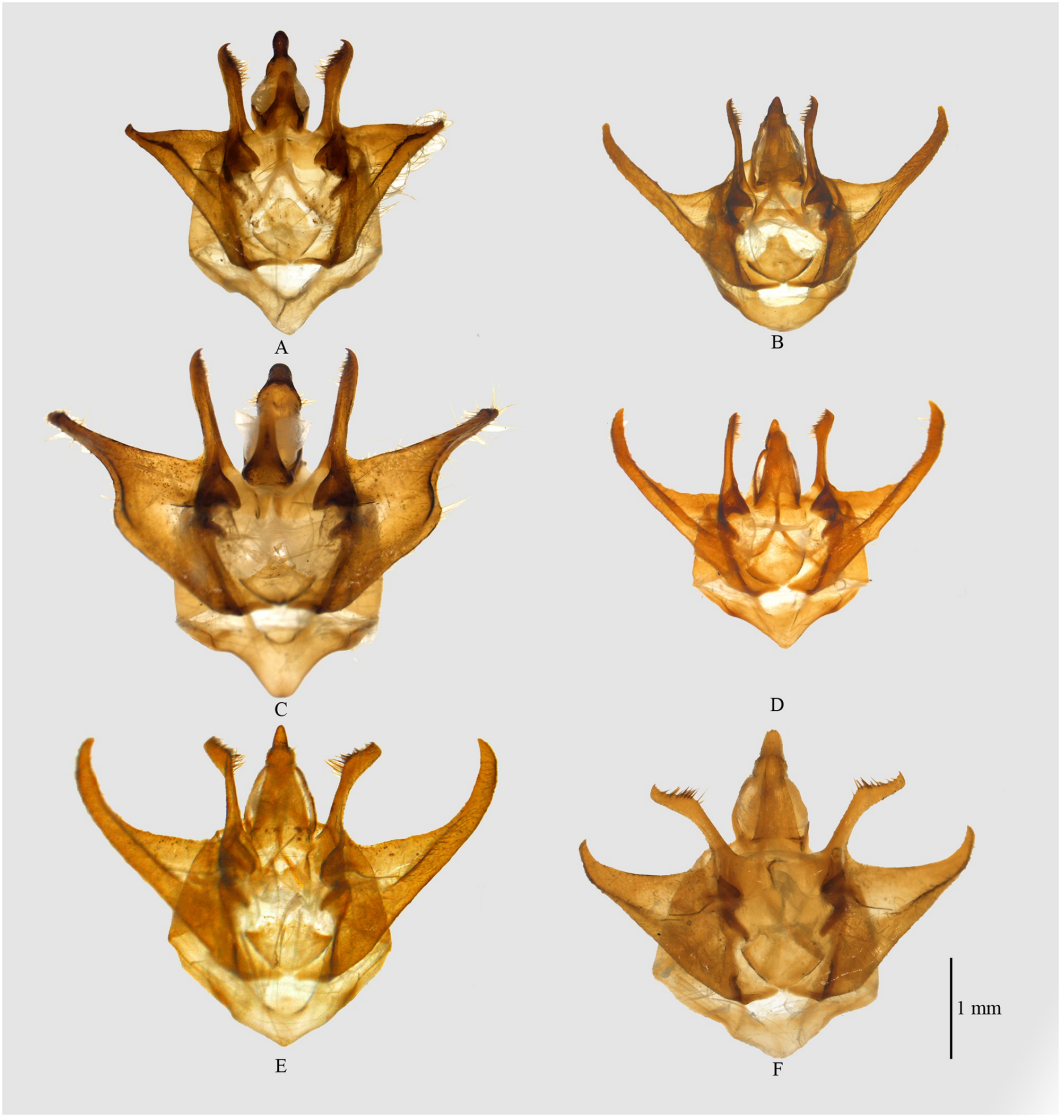
Male genitalia of *Psyra*. A. *P*. *gracilis* B. *P*. *angulifera* C. *P*. *cuneata* D. *P*. *szetschwana* E. *P*. *fulvaria* F. *P*. *similaria*.

**Fig 5:**
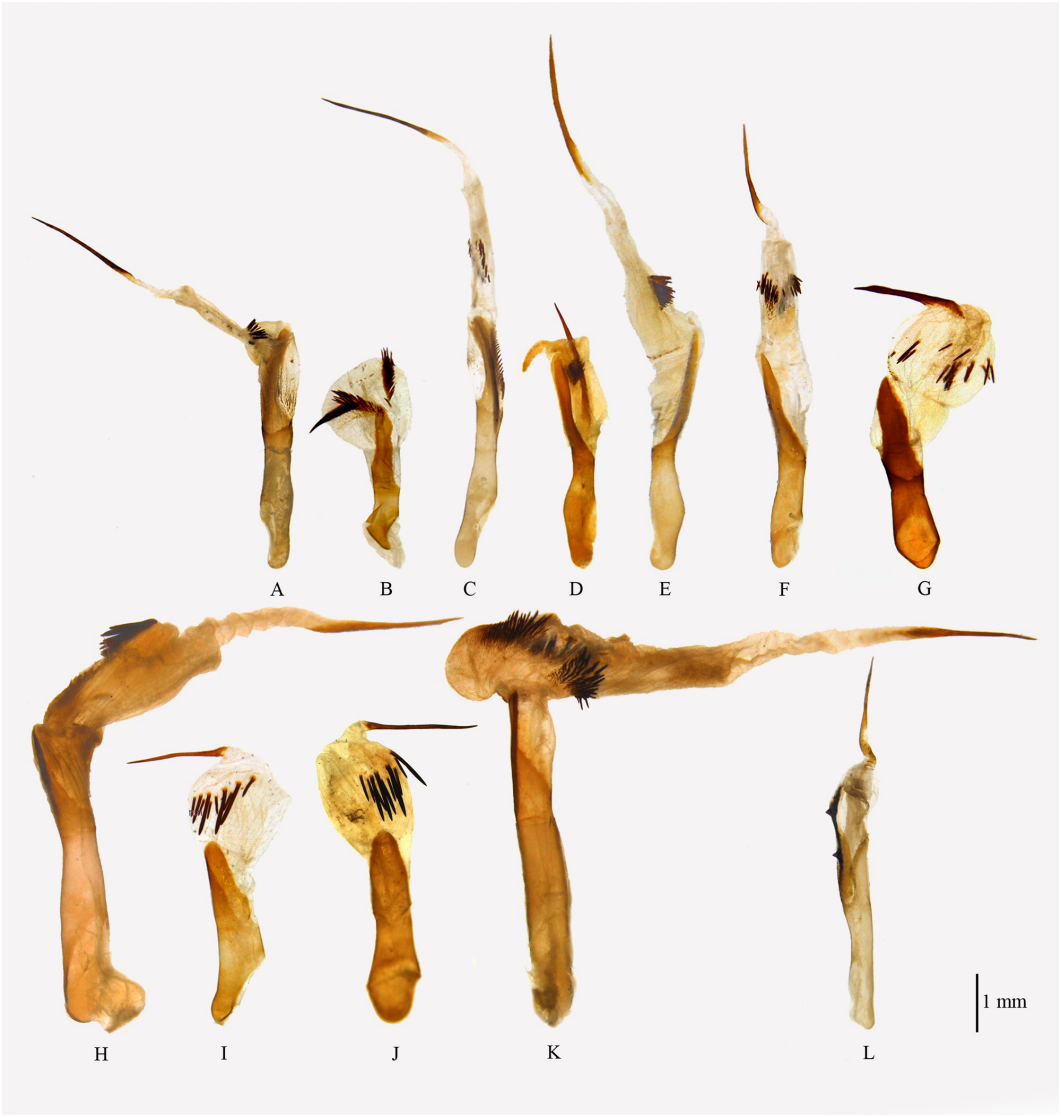
Aedeagus of *Psyra species*. A. *P*. *gracilis* B. *P*. *angulifera* C. *P*. *cuneata* D. *P*. *szetschwana* E. *P*. *fulvaria* F. *P*. *similaria* G. *P*. *similaria* (Dihang Dibang Biosphere Reserve morph) H. *P*. *crypta* I. *P*. *debilis debilis* J. *P*. *debilis indica* K. *P*. *spurcataria* L. *P*. *falcipennis*.

**Fig 6:**
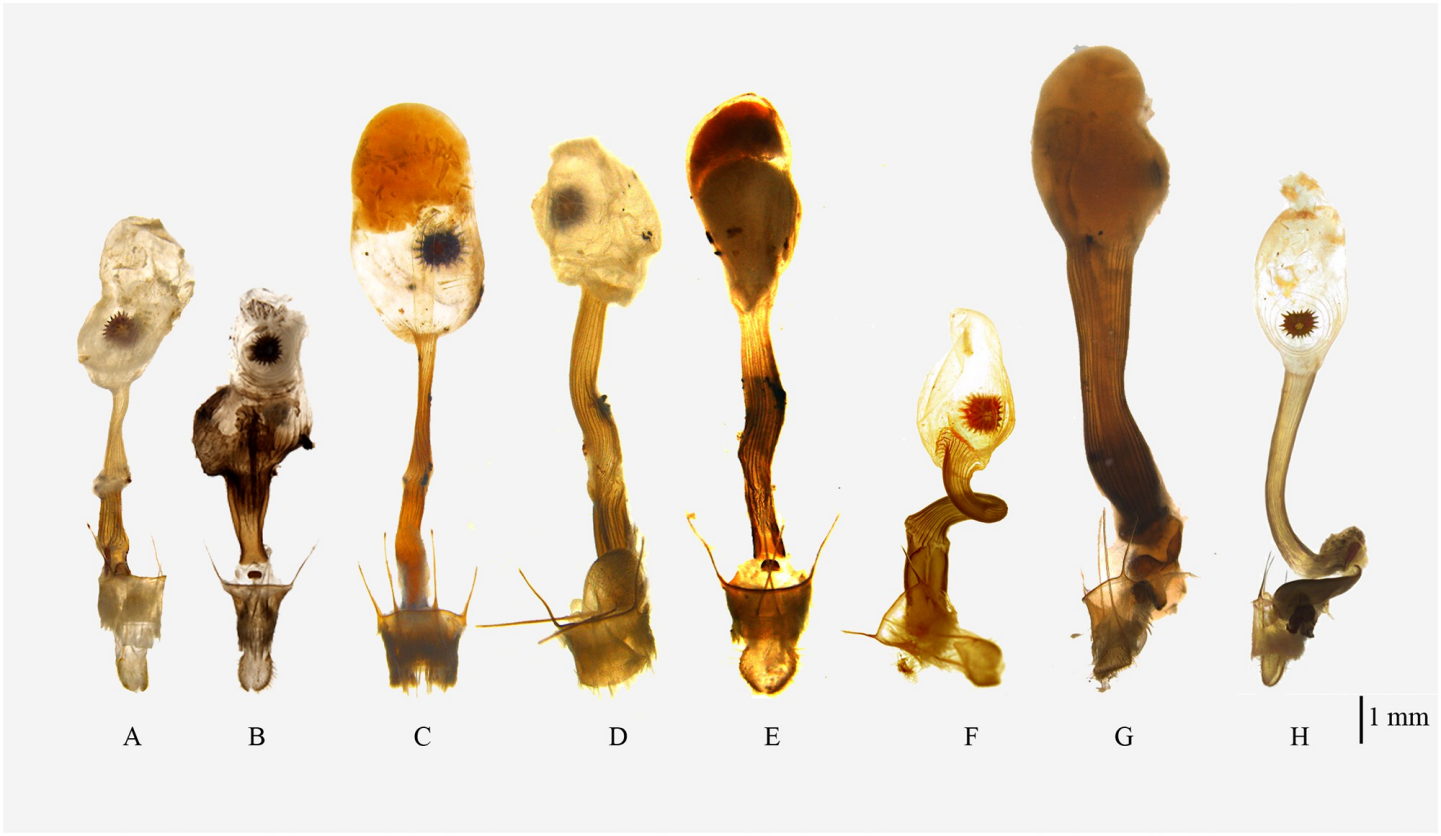
Female genitalia of *Psyra* species. A. *P*. *gracilis* B. *P*. *angulifera* C. *P*. *cuneata* D. *P*. *dsagara* E. *P*. *similaria* F. *P*. *falcipennis* G. *P*. *crypta* H. *P*. *spurcataria*.

**Fig 7:**
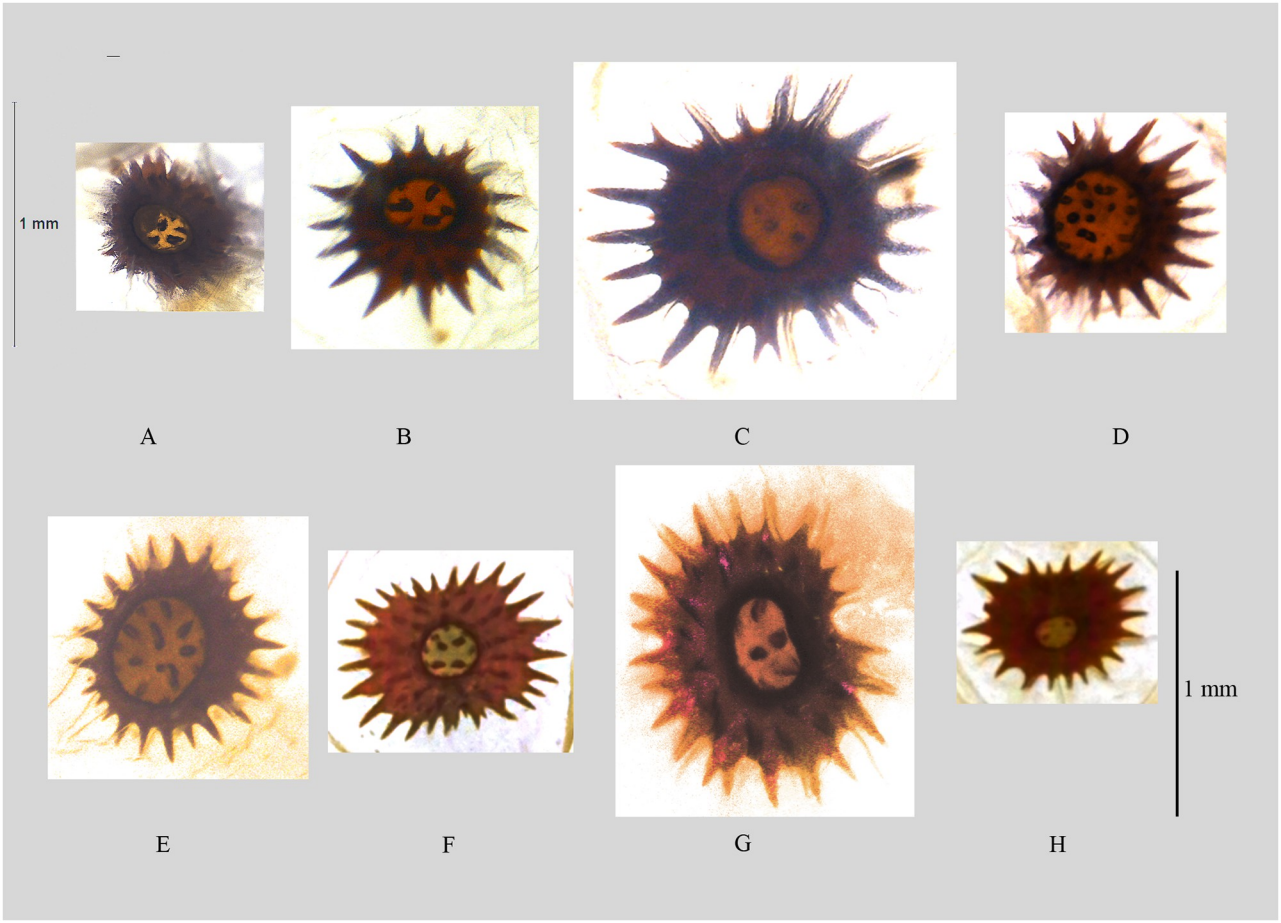
Signum of *Psyra* species. A. *P*. *gracilis* B. *P*. *angulifera* C. *P*. *cuneata* D. *P*. *dsagara* E. *P*. *similaria* F. *P*. *falcipennis* G. *P*. *crypta* H. *P*. *spurcataria*.

(Figs 3A–3C, 4A, 5A, 6A and 7A).

*Psyra gracilis* Yazaki [21]: 35, pl. 11, figs 8, text-fig. 31. Holotype ♂, Nepal: Godavari. (NSMT).

*Psyra gracilis*: [15]: 467.

*Material examined*. INDIA: 1 ♂, Sikkim, Dist. West Sikkim, Khangchendzonga Biosphere Reserve, Yoksum, 27.37864° N, 088.22087° E, 1879 m, 19. XI. 2019, leg. A. K. Sanyal & Team.

1 ♂, West Bengal, Dist. Darjeeling, Singalila National Park, Chitrey, 26.99126° N, 088.11189° E, 2295 m, 15. V. 2018; 1 ♀, Rimbik, 27.1141° N, 088.1105° E, 1905 m, 18. X. 2018; 1 ♀, Dist. Kalimpong, Neora Valley National Park, Suntale Khola, 27.01042° N, 088.78983° E, 760 m, 7. XI. 2016, leg. K. Bhattacharyya & Team.

*Description*. Length of forewing: Male: 19–23 mm; Female: 24–26 mm.

Head, vertex, thorax and patagia bright ochreous; collar with olive tinge; a black spot at the middle of prothorax; two black spots on metathorax; palpi brown, yellow at tips; antennae bright ochreous dorsally and brown beneath; abdomen dorsally greyish-brown and ventrally ochreous, striated with brown and segmental dark patches; fore, mid and hind tibia shining violaceous-brown.

Forewing golden-ochreous, irrorated and suffused with black; costa golden-yellow, basally with black outline and gradually striated with black towards apex; concave series of minute black streaks, most prominent at costal half, represents the impression of basal line; antemedial line, represented by darker impression and two black spots, one just below costa and another on median nervure, a large lunular black patch on vein 2A touching inner margin; bilobed discal spot; postmedial line represented by slightly darker suffusion and composed of eight black specks on each vein, last one is elongated and touching inner margin; submarginal area slightly dark-suffused and with three large black patches: subapical one small, triangular; second one between M_3_ and M_1_, bidentate outwardly, formed by two triangular conjoined patches; last one largest, wedge-shaped, starting below CuA_2_ and extends up to inner margin forming an acute inward angle at vein 2A; a marginal series of seven black dots; cilia bright golden-ochreous.

Hindwing slightly paler golden-ochreous, thickly irrorated and suffused with brown; impression of a medial band just below discal spot; a complete, broad submarginal band and marginal series of black specks; cilia bright golden-ochreous.

Underside pale golden-ochreous, thickly irrorated with brown; prominent discal spot, broad submarginal band and marginal series of black specks in both wings.

Uncus bell-shaped; gnathos broad at the base, robust, not reaching the tip of uncus; valvae triangular, costal margin slightly concave at the subapical region with the projected terminal area bent inwards at apex; ventral margin serrate, nearly straight, strongly sclerotized; costal basal process moderately long, robust, not reaching the tip of the uncus, slightly curved at the apical area and tip hook-like; saccus V-shaped, produced at middle; juxta moderately sclerotized, basally broad; Aedeagus long, stout, straight with ridges of scobination; vesica with three bundles of dense spines and an apical, long, pointed cornutus.

Papilla analis round; apophyses anterior shorter than apophyses posterior; antrum broad, strongly sclerotized; ductus bursae narrow, wrinkled and highly sclerotized, forming an invagination at middle; corpus bursae large, elliptical, bearing signum which consists of multiple short marginal spines and very narrow central space with few small spines.

*Diagnosis*. *P*. *gracilis* can be readily distinguished from other closely related species by the acute inward angle on vein 2A of submarginal wedge-shaped patch; the species is most easily separable from the other group members by its black patches of the forewing not being distinctly outlined by any colour.

In the male genitalia, uncus is more or less like *P*. *cuneata*, but valvae triangular and bears a serrate sclerite at the inner side of the ventral margin. Aedeagus has three bundles of vesica spines as in *P*. *cuneata* and *P*. *spurcataria* but the spines are longer in *P*. *gracilis*.

*Distribution*. **India**: Sikkim (West), West Bengal (Darjeeling, Kalimpong); **Global:** Nepal (Godavari), China (Yunnan) [21, 15]. ***The species is being reported here as new to India*.**

*Bionomy*. In Indian Himalaya, the species is distributed in Central Himalaya with mean abundance at an altitude of 1850 m with most of the individuals recorded between 1300–2200 m though the species range extends between 750 m to 2200 m. Individuals were recorded preferably within a narrow range of annual mean temperature of 15.17°C to 17.7°C and an annual precipitation range between 1600 mm and 4700 mm, though very rarely beyond 2600 mm. Species were active throughout the year mainly in May and in the post-monsoon months of October–November, in wide habitat types ranging from moist mixed deciduous, wet temperate and mixed coniferous forest (S1 Fig).

#### *Psyra angulifera* (Walker, 1866)

(Figs 3D–3G, 4B, 5B, 6B and 7B).

*Scotosia angulifera* Walker [3]: 1687. Holotype ♂, India: Himachal Pradesh, Dharmasala (BMNH).

*Psyra angulifera*: Moore [4]: 659.

*Psyra angulifera*: Butler [6]: 20.

*Psyra angulifera*: Hampson [8]: 222.

*Psyra angulifera*: [45]: 410, pl. 24.

*Psyra angulifera*: [21]: 34.

*Psyra angulifera*: [15]: 461.

*Material examined*. INDIA: 1 ♀, Himachal Pradesh, Dist. Kangra, Dharamshala, 32.23165° N, 076.31414° E, 1492 m, 29. X. 2018, leg. A. Raha & Team; 1 ♀, Dist. Kullu, Great Himalayan National Park, Riush Thatch, 31.78602° N, 077.49277° E, 2425 m, 06. VI. 2018, leg. K. Mallick & Team.

1 ♂, Uttarakhand, Dist. Uttarkashi, Govind Wildlife Sanctuary, Istragad, 31.06386° N, 078.14769° E, 2200 m, 11. V. 2010; 4 ♀♀, Taluka, 31.07802° N, 078.24636° E, 2114 m, 27. X. 2011; 1 ♂, Netwar, 31.07094° N, 078.10628° E, 1405 m, 31. X. 2011; 1 ♂, Dist. Pithoragarh, Askot Wildlife Sanctuary, Syuni, 30.19712° N, 080.2267° E, 2279 m, 07. X. 2017, leg. A. K. Sanyal & Team; 1 ♀, Sarmoli, 30.0769° N, 080.23568° E, 2195 m, 19. X. 2019, leg. U. Bandyopadhyay.

1 ♂, Sikkim, Dist. West Sikkim, Khangchendzonga Biosphere Reserve, Yuksom, 27.37864° N, 088.22087° E, 1879 m, 19. XI. 2019, 1 ♀, 24. XI. 2019; 1 ♂, Khoyngtey, 27.37947° N, 088.22678° E, 1950 m, 27. XI. 2019; 2 ♂♂, Dist. North Sikkim, Khangchendzonga Biosphere Reserve, Rabum, 27.65842° N, 088.60463° E, 2000 m, 13. XII. 2019, leg. A. K. Sanyal & Team.

1 ♂, West Bengal, Dist. Darjeeling, Singalila National Park, Chitre, 26.9913° N, 088.1119° E, 2366 m, 25. VIII. 2016, leg. K. Bhattacharya & Team; 1 ♀, Manedara, 27.11471° N, 088.100° E, 2168 m, 24. X. 2018; 1 ♂, 28. X. 2018; 1 ♀, 3. XI. 2018; 1 ♀, 4. XI. 2018, leg. A. K. Sanyal & Team; 1 ♀, Dist. Kalimpong, Neora Valley National Park, Chaudapheri, 27.0863° N, 088.6596° E, 2600 m, 12. VII. 2018; 1 ♂, Rishop, 27.1073° N, 088.6512° E, 2136 m, 15. VII. 2018, leg. K. Bhattacharya & Team.

1 ♀, Arunachal Pradesh, Dist. Dibang Valley, Dihang Dibang Biosphere Reserve, Engalin, 28.54335° N, 095.63117° E, 925 m, 22. XII. 2016, leg. A. K. Sanyal & Team; 1 ♂, Anini Base, 28.797069° N, 095.909513° E, 1660 m, 14. V. 2018, leg. S. Gayen & Team; 1 ♂, Anini, 28.801828° N, 095.894469° E, 2351 m, 04. IV. 2018, leg. G. N. Das & Team.

*Description*. Length of forewing: Male: 18–21 mm, Female: 21–23 mm.

Head, thorax, patagia and abdomen dark olive-brown; metathorax with two pale-bordered black dots; palpi black at sides, tips yellow; antennae fulvous dorsally and brown beneath. Fore tibia fulvous, mid and hind tibia striated with dark brown, hind tibia dilated with fulvous hair-pencil.

Forewing ground colour varying from pale, creamy-brown to dark purplish-brown; costa golden-brown, a prominent black speck at base; traces of antemedial, postmedial and submarginal lines, composed of triangular black patches outlined with golden-brown and varying in size; antemedial line containing three black lunules; a small, irregular one at R_1_, second one slightly larger, triangular, at base of median nervure and the third one largest, on vein 2A, lunular, lower arm reaching inner margin, the upper arm being much elongated; the postmedial line consists of seven small, black, triangular spots on each vein, slightly incurved below M_2_. The submarginal line having three triangular black patches, one subapical, one at the middle, which is bidentate exteriorly and the third one largest, situated between vein 2A and CuA_2_, not reaching the inner margin and followed by a small black lunule touching the inner margin. Discal spot black, bilobed, bordered with golden-brown; a marginal series of seven black spots.

Hindwing paler with traces of a straight medial line, composed of small black specks on each vein, prominent at abdominal half and obscure at costal half; an obscure cell spot just above the medial line; an indistinct submarginal band; a marginal series of black specks; cilia dark brown.

Underside paler with prominent cell speck, traces of medial line and submarginal band in both wings.

Uncus triangular, broad at base; gnathos broad, long, almost reaching the length of uncus; valvae long, slender, slightly curved at tip; costal margin concave at middle; costal basal process long, narrow, slightly curved with a hooked tip, reaching the length of uncus; saccus rounded, not produced; juxta moderately sclerotized, trowel-shaped. Aedeagus short and stout; vesica with two dense bundles of spines and one short and thick cornutus.

Papilla analis elongated, oval with long setae; apophyses anterior nearly two-third in the length of apophyses posterior; antrum broad; ductus bursae broad, wrinkled and highly sclerotized; corpus bursae large, round, bearing signum which consists of multiple broad marginal spines and few small central spines.

*Diagnosis*. Very **s**imilar to *P*. *cuneata* and *P*. *szetschwana*, but differs in the ground colour of forewing being pale to dark purplish-brown, instead of ochreous. The golden-brown outline of the black patches in the forewing is unique to *P*. *angulifera*.

In the male genitalia, *P*. *angulifera* differs from most closely related *P*. *szetschwana* by having relatively shorter valvae and rounder saccus, which in the case of *P*. *szetschwana* is triangular, expanded in the middle; the aedeagus vesica contains two bunch of spines as in *P*. *szetschwana*, but spines are more in number and the cornutus is shorter, thicker and less pointed.

In the female genitalia, ductus bursae and antrum is broad like *P*. *szetschwana*, but much shorter in size; corpus bursae is significantly large compared to most of the species in the genus.

*Distribution*. **India:** Himachal Pradesh (Kangra, Kullu), Uttarakhand (Uttarkashi, Tehri Garhwal, Pithoragarh), Sikkim (West, North), West Bengal (Darjeeling, Kalimpong), Arunachal Pradesh (Tawang, Dibang Valley), Assam [3, 8, 23, 43, 46, 47]; **Global:** Nepal, China (Hubei, Sichuan, Yunnan, Tibet) [15, 21].

*Bionomy*. The species is widely distributed from North-Western to Eastern Himalaya with mean abundance at an altitude of 2000 m with most of the individuals recorded between 1800–2200 m though the species range extends on rare occasions below 1500 m and up to 2800 m. Mean abundance was recorded at annual mean temperature of 15°C, whereas most of the individuals were recorded between 12.6°C to 16.1°C, though rarely as low as 7.5°C to as high as 22°C. The annual precipitation range of the species was between 750 mm and 2900 mm, while most abundance was observed between 1300 mm to 2300 mm. Species was rarely active before monsoon, with maximum population bloom detected in the post-monsoon season of October–November, mostly in moist temperate deciduous forest of Western Himalaya and in wet temperate forest of Eastern Himalaya (S2 Fig).

#### *Psyra cuneata* Walker, 1860

(Figs 3H–3L, 4C, 5C, 6C and 7C).

*Psyra cuneata* Walker [1]: 483. Holotype ♂, India: North Hindostan (BMNH).

*Psyra cuneata*: Moore [4]: 659.

*Psyra cuneata*: Butler [6]: 20.

*Psyra cuneata*: Swinhoe [43]: 202.

*Psyra cuneata*: Hampson [8]: 223.

*Psyra cuneata*: Leech [9]: 213.

*Psyra cuneata*: Yazaki [21]: 34.

*Psyra cuneata*: [15]: 464.

*Material examined*. INDIA: 4 ♂♂, Sikkim, Dist. West Sikkim, Khangchendzonga Biosphere Reserve, Yuksom, 27.37864° N, 088.22087° E, 1879 m, 19. XI. 2019; 1 ♀, 22. XI. 2019; 2 ♂♂, 1 ♀, Khoyngtey, 27.37947° N, 088.22678° E, 1950 m, 27. XI. 2019; 1 ♂, Dist. North Sikkim, Khangchendzonga Biosphere Reserve, Lingthem, 27.52537° N, 088.49294° E, 1496 m, 11. XII. 2019; 3 ♂♂, 1 ♀, Rabum, 27.65842° N, 088.60463° E, 2000 m, 13. XII. 2019, leg. A. K. Sanyal & Team.

2 ♂♂, West Bengal, Dist. Darjeeling, Singalila National Park, Manedara, 27.11471° N, 088.100° E, 2168 m, 30. X. 2018; 1 ♀, 31. X. 2018, leg. A. K. Sanyal & Team.

1 ♂, Arunachal Pradesh, Dist. Dibang Valley, Dihang Dibang Biosphere Reserve, Chaipani, 29.00368° N, 095.97028° E, 1776 m, 16. IV. 2017; 1 ♀, Etabe, 28.807083° N, 095.934755° E, 1397 m, 15. V. 2018, leg. S. Gayen & Team.

*Description*. Length of forewing: Male: 19–22 mm; Female: 22–24 mm.

Head and thorax dark ochreous-brown; vertex pale ochreous; palpi dark brown with whitish-yellow tip; antennae fulvous dorsally and brown beneath; prothorax with a dorso-medial black spot and metathorax with a pair of dorsal black spots; abdomen dorsally dark ochreous-brown, ventrally pale brown with slight fulvous suffusion, irrorated with brown. Fore tibia fulvous, mid tibia dark brown, hind tibia in male dilated with dark brown hair pencil.

Forewing ground colour dull ochreous-brown, suffused with fuscous; costa golden-yellow, a prominent black speck at base; antemedial, postmedial and submarginal line represented by dark suffusion and black specks and patches; the antemedial formed by a lunular, black patch touching inner margin and two black spots, one at R_1_ and the other at the median nervure; postmedial line consists of seven indistinguishable, minute black specks, one on each vein, the last one being most prominent; submarginal line composed of one subapical, minute triangular spot, followed by two large triangular black patches, one between R_5_ and M_1_, which is bidentate outwardly, third one largest, below vein CuA_2_, touching the inner margin. Two prominent disjoined black discal spots; marginal series of seven black dots; all the black spots and patches of the forewing are outlined with pale ochreous.

Hindwing paler with black discal spot and prominent medial and broad submarginal dark brown bands; a marginal series of black specks; cilia golden-brown.

Underside suffused with brown with a prominent discal spot to both wings; in forewing traces of a postmedial line prominent only towards costa; the two larger submarginal triangular black patches are distinct, sometimes invisible under broad and diffused submarginal band; in hindwing, traces of a medial and submarginal band, which is more prominent in females.

Uncus moderately long, apically stout, blunt and medio-basally broad; gnathos long, triangular at base; valvae broad at middle, costal margin straight, apically narrow, ventral margin undulating, inwardly curved in the apical area, followed by a broad bulge at the middle with small vertical sclerotization and then running almost straight till the base with a slight concavity; costal basal process long, straight, exceeding the length of the uncus, with a hooked tip; saccus highly produced at middle forming a perfect V; juxta moderately sclerotized, basal margin semi-circular; aedeagus long, straight with longitudinal sclerotization and a ridge of short spines at apex; vesica with three bundles of short spines and an apical, very long, thin cornutus.

Papilla analis oval; apophyses anterior shorter than apophyses posterior; ductus bursae long, narrow, wrinkled and highly sclerotized, basally broader than distal; corpus bursae large, ellipsoid, slightly sclerotized, bearing signum which consists of multiple broad marginal spines, some of which are bifurcated and very few small central spines.

*Diagnosis*. *P*. *cuneata* can be easily distinguished from the two similar species *P*. *szetschwana* and *P*. *angulifera* by its broad, dark submarginal band in the hindwing and significantly smaller subapical black spot on forewing submarginal line. The species is also typically characterized by its disjoined discal spots in the forewing.

In the male genitalia, uncus is similar to *P*. *gracilis* but the ventral margin of valvae is distinctly undulating with medial bulge and apical inward curving in *P*. *cuneata*; aedeagus with three bundles of vesica spines like *P*. *gracilis* and *P*. *spurcataria* but in the case of *P*. *cuneata*, spines are not so long. In the female genitalia, the ductus bursae is narrow and long; corpus bursae large and roundish; the junction between antrum and ductus bursae is slightly expanded.

*Distribution*. **India:** North-West Himalaya, Sikkim (West, North), West Bengal (Darjeeling), Arunachal Pradesh (Dibang Valley), Meghalaya (East Khasi) [8, 46]; **Global:** Nepal, China (Yunnan, Tibet), Taiwan, Japan [9, 15, 21].

*Bionomy*. The species is distributed in Central and Eastern Himalaya with maximum abundance between 1850 m to 2150 m altitudes, though rarely the species was recorded at 2800 m. Most individuals were active between annual mean temperature of 13–16°C and annual precipitation of 1500 mm to 2400 mm, though the range extended within 10 to 19°C and 900 mm to 3500 mm. The species was very rare in pre-monsoon months of April–May but very abundant in October–November, mostly in East Himalayan wet temperate forest habitats (S3 Fig).

#### *Psyra szetschwana* Wehrli, 1953

**Fig 8:**
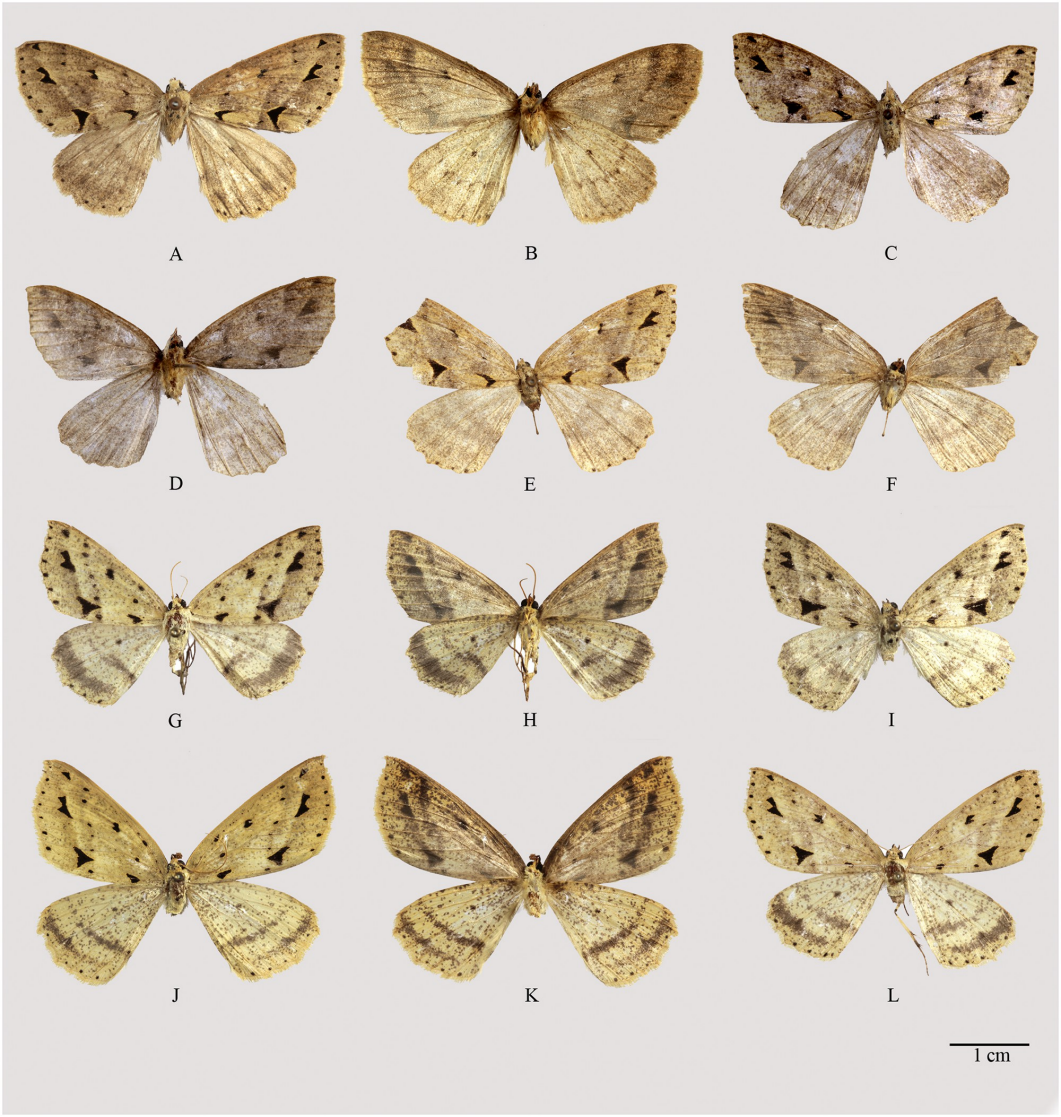
Habitus of *Psyra szetschwana*, *P*. *fulvaria*, *P*. *dsagara*, *P*. *similaria*. A. *P*. *szetschwana* ♂ B. *P*. *szetschwana* ♂ underside C. *P*. *fulvaria* ♂ D. *P*. *fulvaria* ♂ underside E. *P*. *dsagara* ♀ F. *P*. *dsagara* ♀ underside G. *P*. *similaria* ♂ H. *P*. *similaria* ♂ underside I. *P*. *similaria* ♂ (variation from Dihang Dibang Biosphere Reserve) J. *P*. *similaria* ♀ K. *P*. *similaria* ♀ underside L. *P*. *similaria* ♀ (pale morph).

(Figs 4D, 5D, 8A and 8B).

*Psyra cuneata szetschwana* Wehrli [14]: 670. Syntype (s), China: Szetschwan, Tachien-lu (ZFMK).

*Psyra cuneata lidjangica* Wehrli [14]: 671. Syntype (s), China: Yunnan: Likiang (ZFMK). syn. nov.

*Psyra szetschwana*: [15]: 465. stat. rev.

*Material examined*. INDIA: 1 ♂, Arunachal Pradesh, Dist. Dibang Valley, Dihang Dibang Biosphere Reserve, Etabe, 28.80089° N, 095.95936° E, 2273 m, 06. IV. 2017, leg. S. Gayen & Team.

*Description*. Length of forewing: Male: 21 mm.

Head, thorax and patagia pale ochreous; vertex pale ochreous; palpi dark brown with fulvous at tips; antennae fulvous dorsally and brown beneath; abdomen dorsally brown, ventrally ochreous. Fore tibia fulvous, mid tibia dark brown, hind tibia dilated with dark brown hair pencil.

Forewing ground colour brownish-ochreous with fuscous suffusion, apex not falcate with rounder outer margin without any outward angle at M_3_; costa golden-yellow, a prominent black speck at base; traces of an antemedial, a curved postmedial and a submarginal line; the antemedial with two black spots, one subcostal and the other at the middle, another elongated lunular black patch reaching inner margin, upper arm being slightly extended; the postmedial line slightly incurved below M_2_, consisting of seven small, black specks; submarginal having three triangular black patches, one subapical, distinctly triangular, the second one between veins M_3_ and M_1_, which is bidentate exteriorly and the third largest one between vein 2A and CuA_2_; discal spot black, bilobed, triangular in shape and conjoined; a marginal series of seven black dots; all the black marks are pale yellow-bordered.

Hindwing pale ochreous with blackish discal speck; a double yet well-separated medial band, originating from a single black speck on inner margin, first one straight, just below the discal spot and indistinguishable above M_3_, second one curved, complete and more post-medial in position; a broad indistinct submarginal fuscous band; a marginal series of black specks; cilia golden-brown.

Underside paler with distinct discal spots in both wings, in forewing double, while single in hindwing; curved postmedial and submarginal line in the forewing, prominent on costal half and almost parallel to each other; in hindwing the postmedial line is distinctly crenulated followed by an indistinct, broad submarginal band; marginal series of black specks and thin, golden-ochreous cilial lines to both wings.

Uncus triangular, tip blunt, broad at base; gnathos broad, long, almost reaching the length of uncus; valvae long, slender, scimitar-shaped, curved and pointed at the tip, triangular basally; costal basal process long, narrow, the length reaching beyond uncus, tip swollen, truncated on the inner side with prominent sclerotization; saccus protruded in the middle; juxta moderately sclerotized; aedeagus short and stout; vesica with two bundles of spines and one short and thin, curved cornutus.

*Diagnosis*. *P*. *szetschwana* can be distinguished from *P*. *cuneata* by its conjoined discal dots in the forewing and double medial band of the hindwing. The first submarginal spot distinct and triangular, while they are obscure in *P*. *cuneata*; the last submarginal patch in *P*. *cuneata* is so large that its inner edge crosses the postmedial line, while in *P*. *szetschwana* it is smaller and never crosses the postmedial line. The submarginal band of the hindwing is indistinct in *P*. *szetschwana*, which is rather distinct in *P*. *cuneata*. The crenulated postmedial line in the hindwing underside is also typical of *P*. *szetschwana*.

In the male genitalia, valvae is similar to *P*. *angulifera* and *P*. *fulvaria*, but differs from *P*. *angulifera* by being more apically curved and from *P*. *fulvaria* by being shorter; the tip of the costal basal process is swollen in *P*. *fulvaria* and *P*. *szetschwana* but tapering in case of *P*. *angulifera;* saccus is also more protruding in *P*. *szetschwana* than in *P*. *angulifera;* aedeagus has two bundles of spines and one apical spine as in *P*. *angulifera* instead of one bundle of the spine in *P*. *fulvaria*.

*Distribution*. **India:** Arunachal Pradesh (Dibang Valley); **Global:** China (Shaanxi, Sichuan, Yunnan) [14, 15]. ***The species is being reported here as new to India*.**

*Bionomy*. The species is probably very rare in Indian Himalaya, discovered in this study only from the Eastern Himalayan habitat of wet temperate forest in the pre-monsoon month of April, with the night time temperature at 29°C and relative humidity of 68%. The altitudinal range of the species extends between 2250 m to 3500 m, with mean abundance at 3200 m. Being mostly a high-altitude specific species, it was recorded at low annual mean temperature range of 7°C to 10°C and very low annual precipitation of 800 mm (S4 Fig).

#### *Psyra fulvaria* Yazaki, 1992

(Figs 4E, 5E, 8C and 8D).

*Psyra fulvaria* Yazaki [21]: 35, pl. 11, figs 7, text-fig. 32. Holotype ♂, India: West Sikkim, Bakkhim (2670m).

*Material examined*. INDIA: 1 ♂, West Bengal, Dist. Darjeeling, Singalila National Park, Meghma, 27.01586° N, 088.08993° E, 2733 m, 18. V. 2018; 1 ♂, Gairibash, 27.05093° N, 088.03366° E, 2494 m, 21. V. 2018, leg. A. K. Sanyal & Team.

*Description*. Length of forewing: Male: 19.5–21.5 mm.

Head, vertex, thorax and patagia fulvous; palpi dark brown, yellow at tips; antennae ochreous dorsally and brown beneath; abdomen dorsally brown, ventrally light ochreous, striated with brown.

Forewing ground colour pale golden-ochreous with brown suffusion; costa golden-yellow with slight fuscous suffusion; the antemedial with three black spots, one just below the costa, second one larger on median nervure and another elongated triangular black patch on vein 2A up to inner margin, with much elongated upper arm; the postmedial line very indistinct, slightly incurved below M_1_, formed of seven minute, black specks on each vein; the submarginal area with three triangular, large, black patches: first below apex; second, bidentate exteriorly, between M_3_ and M_1_ and the third one largest one between 2A and CuA_2_ with elongated lower arm reaching inner margin. Discal spots black, formed of two conjoined triangular patches; a marginal series of seven black dots; all the black marks are bordered with pale golden-yellow.

Hindwing darker with indistinct brown cell spot; an obscure, single medial line and a broad indistinct submarginal fuscous band; a marginal series of black specks; cilia pale ochreous.

Underside pale ochreous with indistinct cell spot and crenulated postmedial line to each wing, the line is more prominent in hindwing; other markings as in upper side.

Uncus long, triangular, broad at the base, tip pointed; gnathos long, apically narrow, tongue-shaped; valvae long, crossing the length of the uncus, much curved apically, costal margin concave, with slight dentation, ventral margin straight, much sclerotized; costal basal process long, swollen at the tip, inner side truncate at the subapical region, curved apically; saccus broad, triangular with pointed tip; juxta moderately sclerotized, semi-circular at the base, upper half triangular with two lateral, medial projections; aedeagus slender, long, apically pointed; vesica with one bundle of short spines and a terminal, thin, acute cornutus.

*Diagnosis*. In outer morphology, *P*. *fulvaria* is most similar to *P*. *szetschwana*, but the former is smaller, having more falcate forewing apex with outer margin distinctly angular at M_3_, while *szetchwana* is larger, with the forewing apex less falcate and the outer margin roundish, not angulated. *P*. *fulvaria* has single dark medial band in hindwing, which is double in *P*. *szetschwana*. In the forewing underside, the crenulated postmedial line is almost straight and oblique in *fulvaria*, while it is incurved below M_3_ in *szetschwana*. In hindwing also, the crenulation and curvature of the postmedial line are less than that of *szetschwana*.

In the male genitalia, *P*. *fulvaria* has similar long and slender, scimitar-shaped valvae like *P*. *angulifera* and *P*. *szetschwana*, but the valvae is distinctly longer and apically curved in *fulvaria* compared to *angulifera*. The main difference of *fulvaria* with *szetschwana* lies in the shape of saccus where it is without any medial protrusion in *szetschwana*, but with the pointed tip in *fulvaria*, aedeagus of *fulvaria* has only one bundle of spine whereas both *angulifera* and *szetschwana* have two bundle of spines.

*Distribution*. **India:** Sikkim (West), West Bengal (Darjeeling) [21]; **Global:** Nepal (Janakpur) [21].

*Bionomy*. The species is currently known only from the Central Himalaya between a narrow altitude range of 2300–2500 m, rarely up to 2600 m. Species was recorded between 12–13°C annual mean temperature and 1900–2300 mm of annual precipitation, only in the pre-monsoon month of May from wet temperate and mixed coniferous forests (S5 Fig).

#### *Psyra dsagara* Wehrli, 1953

(Figs 6D, 7D, 8E and 8F).

*Psyra cuneata dsagara* Wehrli [14]: 671. Lectotype ♂, China: Tibet (north-east): Dsagar Mountain (ZFMK).

*Psyra dsagara*: [15]: 466. stat. rev.

*Material examined*. INDIA: 1 ♀, West Bengal, Dist. Darjeeling, Singalila National Park, Gairibash, 27.05093° N, 088.03366° E, 2494 m, 21. V. 2018, leg. K. Bhattacharya & Team.

*Description*. Length of the forewing: Female: 20 mm.

Head, thorax and patagia pale ochreous; palpi dark brown, pale ochreous at sides; antennae filiform, dorsally ochreous and ventrally brown; mesothorax with white scales; metathorax with two black spots; abdomen ochreous-brown, segments marked with black; all the legs brown, joints marked with black.

Forewing apex not falcate, angulation at M_3_ not much pronounced; ground colour ochreous and very sparsely striated with black, costa golden-yellowish, irrorated with blackish-brown streaks. An antemedial line composed of three golden bordered black spots, 1^st^ one below cost, 2^nd^ one on median nervure and 3^rd^ one lunulate, on CuA_2_, the upper arm elongated, lower arm touching the inner margin; discal spot black, double and conjoint; a postmedial series of seven round, blackish specks on each vein; submarginal line composed of three large, golden bordered, black, triangular patches; 1^st^ on just below the costa, 2^nd^ one between M_3_ and M_1_, bidentate outwardly, 3^rd^ one largest, below CuA_1_, its lower arm touches the inner margin; a marginal series of seven black spots; cilia golden-ochreous.

Hindwing paler, thickly irrorated with fuscous, which is denser towards base; a prominent large, black, discal spot; a slightly curved, complete, brownish, medial line; a curved postmedial line composed of series of brownish vein blotches; a broad, complete, purplish-brown submarginal band, broader and darker toward costa; series of seven marginal black specks.

Underside much paler, forewing with costa golden-yellow; traces of a discal dot; a curved postmedial and submarginal lines; Hindwing with faint discal speck and prominent crenulated postmedial line.

Papilla analis oval; apophyses anterior shorter than apophyses posterior; ductus bursae long, relatively broad, wrinkled and sclerotized; corpus bursae round, relatively small, bearing signum which consists of multiple broad marginal spines and broad central region with multiple minute spines.

*Diagnosis*. In external appearance, *P*. *dsagara* is most similar to *P*. *similaria* and *P*. *szetschwana*. It differs from *P*. *szetschwana*, having the hindwing discal spot quite distant from the medial line, while in *P*. *szetschwana*, the medial line is immediately after or almost touches the discal spot. The last submarginal triangular, black patch of forewing touches the inner margin, while in *P*. *similaria* it never reaches the inner margin. In hindwing, a curved postmedial line composed of series of brownish vein blotches is the unique character of this species.

In the female genitalia, *P*. *dsagara* is unique, having the central space of signum very much broader, leaving the marginal area quite narrow, while in, *P*. *similaria* and *P*. *szetschwana*, the central space is not so broad. Ductus bursae is also quite narrow in *P*. *dsagara*, compared to *P*. *similaria* and *P*. *szetschwana*.

*Distribution*. **India:** West Bengal (Darjeeling); **Global:** China (Sichuan, Hunan, Yunnan) [14, 15]. ***The species is being reported here as new to India*.**

*Bionomy*. The species is extremely rare in Indian Himalaya recorded in this study only from the Central Himalayan wet temperate forest in the pre-monsoon month of May, with night temperature at 12.24°C and relative humidity of 93.7%. The altitudinal range of the species extends between 1600 m to 2600 m, on rare occasion recorded from 4500 m. The species occurs within a wide range of annual mean temperature between 2°C to 13°C, preferably within 8°C to 12°C, while, 800–1000 mm annual mean precipitation, rarely up to 2300 mm characterize the species occurrence (S6 Fig).

#### *Psyra similaria* Moore, 1867

**Fig 9:**
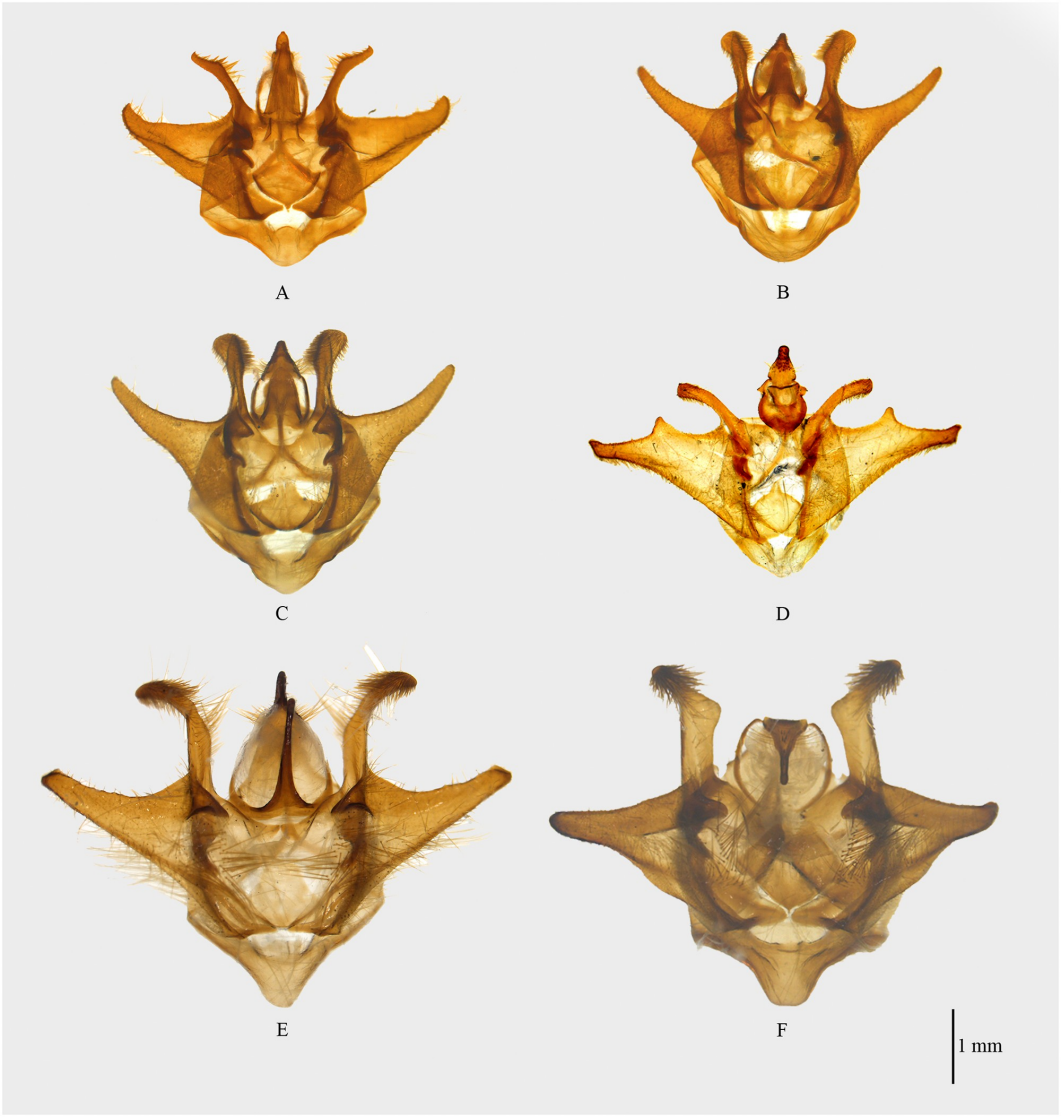
Male genitalia of *Psyra*. A. *P*. *similaria* (Dihang Dibang Biosphere Reserve morph) B. *P*. *debilis debilis* C. *P*. *debilis indica* D. *P*. *falcipennis* E. *P*. *crypta* F. *P*. *spurcataria*.

(Figs 4F, 5F, 5G, 6E, 7E, 8G–8L and 9A).

*Psyra similaria* Moore [4]: 659, pl. 33, fig 1. Syntype (s) ♂, India: Darjeeling (BMNH).

*Psyra similaria*: [46]**:** 513.

*Psyra similaria*: [6]: 20.

*Psyra similaria*: [43]: 202.

*Psyra similaria*: [8]: 223.

*Psyra similaria*: [22]: 26.

*Psyra similaria*: [15]: 467.

*Material examined*. INDIA: 1 ♀, Himachal Pradesh, Dist. Kullu, Great Himalayan National Park, Denga Pool, 31.78400° N, 077.44241° E, 1970 m, 26. V. 2018; 2 ♂♂, 1 ♀, 24. IX. 2019; 1 ♀, Vred Nala, 31.77133° N, 077.47926° E, 2800 m, 08. VI. 2018; 1 ♂, Thati, 31.73912° N, 077.35873° E, 2917 m, 08. IX. 2016, leg. K. Mallick & Team.

4 ♂♂, Uttarakhand, Dist. Uttarkashi, Govind Wildlife Sanctuary, Taluka, 31.07103° N, 079.27039° E, 2460 m, 09. VI. 2012; 1 ♂, 31.0675° N, 078.2519° E, 2605 m, 12. VI. 2012, leg. A. K. Sanyal; 1 ♂, Dist. Chamoli, Valley of Flowers National Park, Ghangaria, 30.70122° N, 079.59402° E, 3128 m, 28. VIII. 2016, leg. H. Kumar & Team; 1 ♂, Dist. Pithoragarh, Askot Wildlife Sanctuary, Babaldhar, 30.17830° N, 080.23179° E, 2442 m, 28. IX. 2017; 1 ♀, Jimjhini, 29.92121° N, 080.39719° E, 2627 m, 18. VI. 2018; 2 ♂♂, 1 ♀, Ringal Forest, 29.92378° N, 080.3930° E, 2800 m, 20. VI. 2018, leg. A. K. Sanyal & Team.

1 ♂, West Bengal, Dist. Darjeeling, Singalila National Park, 1 ♀, Rimbik, 27.1141° N, 088.1105° E, 1905 m, 18. X. 2018; Manedara, 27.11471° N, 088.100° E, 2168 m, 3. XI. 2018, leg. A. K. Sanyal & Team.

1 ♀, Arunachal Pradesh, Dist. Dibang Valley, Dihang Dibang Biosphere Reserve, Meyhoopey, 28.787358° N, 095.954177° E, 2314 m, 17.V.2018; 1 ♂, Karoya, 28.778077° N, 095. 950280° E, 2822 m, 04. VI. 2018; 1 ♂, Ahipu, 28.771627° N, 095.948833° E, 3800 m, 12. VI. 2018; leg. S. Gayen & Team.

*Description*. Length of forewing: Male: 18–21 mm; Female: 21–23 mm.

Head, thorax and abdomen pale ochreous; palpi black, golden-yellow at tips; antennae dorsally yellow and ventrally brown; fore tibia pale ochreous, mid tibia brown, hind tibia ochreous, striated with brown. Prothorax with a single black dot, metathorax with two black dots.

Forewing golden-straw coloured, costa black; a black dot at base. Three prominent, elongated, black spots at the antemedial area, first one small, elliptical, at R_1_; second one at median nervure; the third one is large, on vein 2A and not reaching inner margin. Postmedial line slightly curved, formed of seven black dots on each vein. The submarginal area having three black, triangular, large spots: one subapical, distinctly triangular; second one bidentate, between M_3_ to M_1_ and the third one below CuA_2_ up to 2A, not reaching the inner margin, while the inner angle elongated towards the base and almost crosses the postmedial line. Discal spot black, considerably large, outwardly bidentate; a marginal series of seven black specks; cilia concolourous with the ground colour.

Hindwing much paler, sparsely irrorated with fuscous or brown, which is denser towards base; a prominent dark brown cell spot far above the medial line; an incompletely double, fuscous medial line originating from a black speck on inner margin, initially fused together up to 2A, upper arm very indistinct, straightish, not visible above median nervure, the lower one almost complete, slightly curved, reaching the costa; submarginal band broad towards costa, becoming invisible up to anal angle; marginal series of blacks specks; cilia pale ochreous.

Underside thickly irrorated and suffused with black; in the forewing, basal half above inner margin slightly tinged with purplish; dark and almost parallel postmedial and submarginal lines; hindwing less suffused and striated, with a prominent discal spot, followed by the parallel medial and submarginal band, latter much broad toward costa; both wings with marginal black specks in interneural spaces.

Uncus long, slender; gnathos long, narrow, reaching the tip of uncus; valvae short, broad, costal margin concave, apex slightly curved inwards; costal basal process long, almost reaching uncus, inner side truncate at the subapical region, tip hook-like; saccus broad, protruded at middle; juxta rhomboid, sclerotized; aedeagus long, slender; vesica with two bundles of dense spines of varying length and one terminal, moderately long, pointed cornutus.

Papilla analis oval; apophyses anterior shorter than apophyses posterior; ductus bursae long, medio-ventrally swollen, wrinkled and sclerotized; corpus bursae oval, moderately large, sclerotized, bearing signum with multiple broad irregular marginal spines and few minute central spines.

*Diagnosis*. In the outer morphology, *P*. *similaria* is most similar to *P*. *fulvaria*, but can be easily separable from it by having prominent hindwing medial and submarginal band, while in the latter, the hindwing is uniformly dull fuscous; the underside is also much contrastingly patterned in *P*. *similaria*.

Male genitalia very similar with *P*. *fulvaria* but valvae is not so long in *P*. *similaria*, saccus is medially protruded in *similaria*, while it is with the pointed tip in *fulvaria*; uncus as in *P*. *fulvaria* but gnathos relatively less broad; aedeagus with two bundles of spines like *P*. *szetschwana* and *P*. *angulifera* instead of one in *P*. *fulvaria*. In the female genitalia, the ductus bursae is medio-ventrally swollen and the corpus bursae is oval.

*Distribution*. **India:** Himachal Pradesh (Kullu), Uttarakhand (Uttarkashi, Pithoragarh), Sikkim, West Bengal (Darjeeling), Arunachal Pradesh (Dibang Valley), Meghalaya (East Khasi), [4, 23, 46]; **Global:** Nepal (Kosi, Sagarmatha, Janakpur), China (Tibet) [15, 22].

*Bionomy*. The species is very common across the Indian Himalaya from north-western to eastern sector throughout a wide altitudinal range of 1900–3100 m, with the majority of individuals recorded between 2300 m to 2800 m and in very rare occasion from 3800 m. Major abundance was recorded between annual mean temperature of 10°C to 15°C, however, the species occurs at a much wide temperature range of 8°C to 21°C. The annual precipitation range of the species was very wide between 650 mm and 2500 mm, while majority of the individuals were recorded between 1100 mm to 1600 mm. Species is active throughout the year, though maximum abundance was detected in the monsoon months of June to September. In Western Himalaya, the species was mostly active in moist temperate deciduous forest, upper oak-fir forest and mixed coniferous forest, while eastwards, the species was recorded mostly from the wet temperate forest and rarely from the subalpine birch-fir forest in Arunachal Pradesh (S7 Fig).

#### *Psyra debilis debilis* Warren, 1888

**Fig 10:**
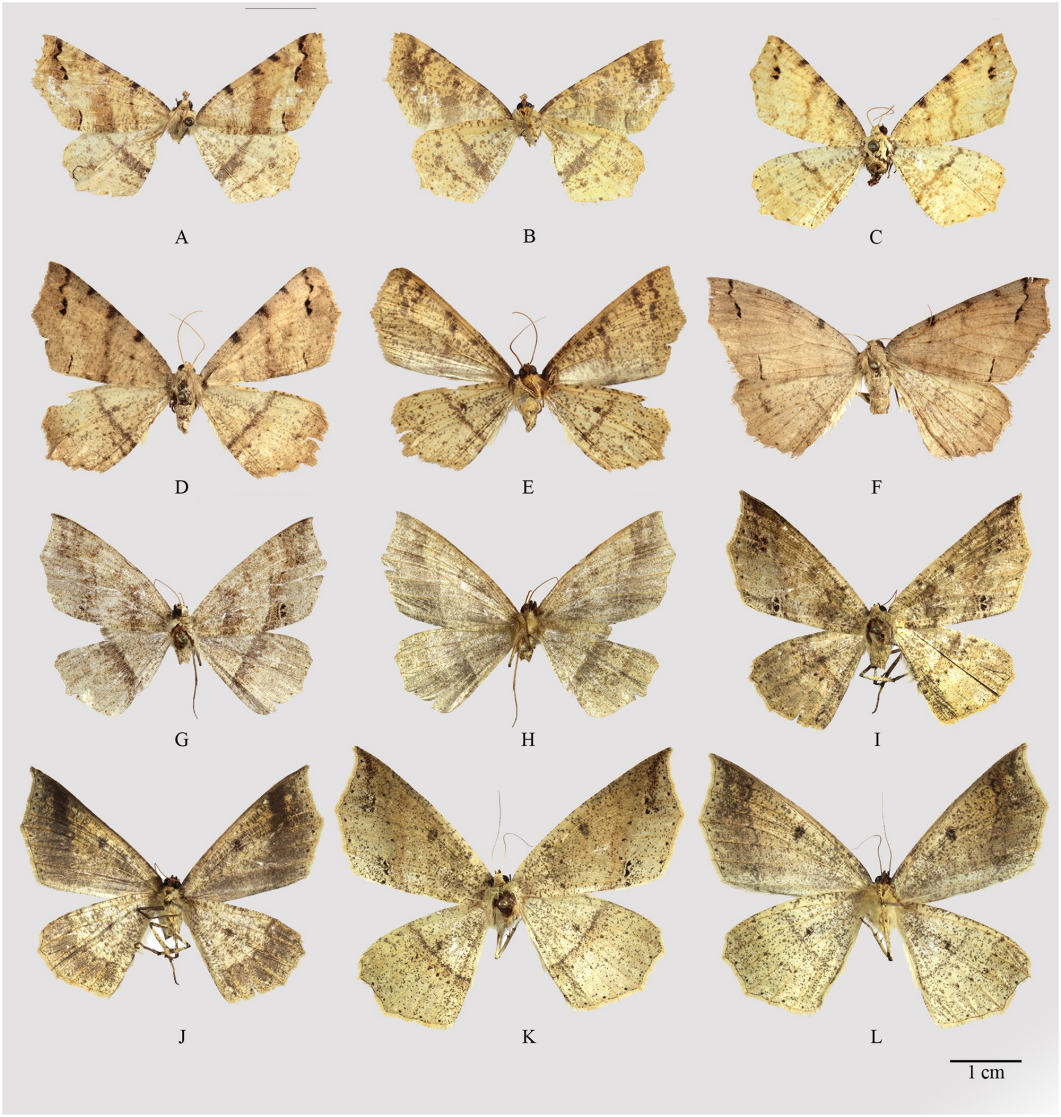
Habitus of *Psyra debilis debilis*, *P*. *debilis indica*, *P*. *falcipennis*. A. *P*. *debilis debilis* ♂ B. *P*. *debilis debilis* ♂ underside C. *P*. *debilis debilis* ♂ D. *P*. *debilis indica* ♂ E. *P*. *debilis indica* ♂ underside F. *P*. *debilis indica* ♂ G. *P*. *falcipennis* ♂ H. *P*. *falcipennis* ♂ underside I. *P*. *falcipennis* ♀ J. *P*. *falcipennis* ♀ underside K. *P*. *falcipennis* ♀ (pale morph) L. *P*. *falcipennis* ♀ underside.

(Figs 5I, 9B and 10A–10C).

*Psyra debilis* Warren [5]: 319. Syntypes 3 ♂(BMNH), Pakistan: Thundiani.

*Psyra debilis*: [8]: 222.

*Psyra debilis debilis*: [7]: 807.

*Material examined*. INDIA: 2 ♂♂, Ladakh, Dist. Kargil, Kukstay, 34.48827° N, 076.27300° E, 3149 m, 30. V. 2018; 1 ♂, Silmo, 34.62106° N, 076.30881° E, 3408 m, 31. V. 2018; 1 ♂, Sanjak, 34.58132° N, 076.52644° E, 2807 m, 03. VI. 2018; 2 ♂♂, Wakaha Choskor, 34.37156° N, 076.38313° E, 3321 m, 18. VI. 2018; 1 ♂, Skamboo, 34.45939° N, 076.23881° E, 3365 m, 28. VII. 2019, leg. M. Ali.

*Description*. Length of forewing: Male: 18–22 mm.

Head, frons, thorax and patagia ochreous; palpi brown; antennae dorsally yellow and brown beneath; metathorax with two brown dots; abdomen pale ochreous dorsally with brown irroration and black segmental bands, ventrally paler; fore, mid and hind tibia ochreous with brown irroration.

Forewing ochreous-brown, slightly irrorated with black; costa golden-ochreous, striated with black, more towards base; four prominent blackish-brown costal blotches at antemedial, medial, postmedial and submarginal area; the antemedial line, starting from the costal blotch is slightly incurved till vein 2A, then straight to inner margin; a faintly curved medial and postmedial line arising from the respective costal blotches reaching inner margin, the space between them suffused with brown forming a medial band which is wider and less prominent at costa; a submarginal waved, black line originating from the submarginal costal blotch, having a prominent outward teeth on R_5_ followed by two continuous black spots on M_1_ and M_2_, after which becoming obsolete and again reappearing below CuA_2_, becoming slightly excurved till inner margin; sometimes a pale brownish suffusion beyond submarginal line; inner margin with series of black spots; a series of small black marginal specks; cilia golden-ochreous.

Hindwing straw-coloured, striated with brown which is denser at basal area; outer margin produced at M_3_; a prominent medial band broad till M_3_ and continued up to costa; a brownish postmedial line with angulation at M_1_, then slightly bent inward till costa; a blackish-brown spot on this line just below M_1_; marginal series of black specks.

Underside golden-yellowish, thickly striated with brown; a fuscous-brown discal spot to each wing; traces of antemedial, medial, postmedial and submarginal lines in the forewing, all prominent towards costa, the last two becoming fused at R_5_; hindwing with the prominent medial and submarginal line as in upper side.

Uncus bell-shaped, basally broad, apically acute; gnathos tongue-like, broad, sclerotized, not reaching the tip of uncus; valvae triangular, slender, costal margin concave with pointed apex; costal basal process straight, almost reaching the length of the uncus, with setose and rounded apex; saccus broad and U-shaped; juxta broad, marginally sclerotized; aedeagus short, more or less straight; vesica with a terminal, moderately long spine and a bunch of spines at the sub-basal region.

*Diagnosis*. *P*. *debilis debilis* can be distinguished from its other subspecies *P*. *debilis indica* by its smaller size, the ground colour being more golden, the brown postmedial band of the forewing, pale brown suffusion after the submarginal line, the broad medial band instead of wavy line in hindwing; There is also a discal spot in the hind wing just touching the medial line in *P*. *d*. *indica*. In the male genitalia, *P*. *debilis* is typically characterized by basally broad uncus; tongue like gnathos, apically bulbous costal basal process and U-shaped saccus.

*Distribution*. **India:** Ladakh; **Global:** Pakistan (Thundiani) [5, 8].

*Bionomy*. This is the only species of *Psyra* in the Indian Himalaya recorded from the Trans-Himalayan zone of Ladakh, restricted mainly to different alpine habitats like deciduous alpine scrub and dwarf juniper scrub. Altitudinal preference of the species is very unique, restricted between 2900 m to 3500 m with annual mean temperature range of 15°C to 17°C. Being typically Trans-Himalayan, the species habitable area is characterized by very low annual mean precipitation of 250 mm and very low nightly relative humidity ranging from 16% to 35%. In accordance with the very short growing season in Ladakh, the species was active between May to July, though it was also reported fairly common during months of August and September, the period when it was originally described (S8 Fig).

#### *Psyra debilis indica* (Butler, 1889)

(Figs 5J, 9C and 10D–10F).

*Tetracis indica* Butler [6]:20, 99, pl. 135, fig 16, syntype ♀ (BMNH), India: Dharamshala.

*Psyra indica*: [6]: 222.

*Psyra indica*: [48]: 14.

*Psyra debilis indica*: [7]: 806.

*Material examined*. INDIA: 1 ♂, Himachal Pradesh, Dist. Kullu, Great Himalayan National Park, Shakti, 31.78837° N, 077.49081° E, 2137 m, 7. IV. 2018; 1 ♂, Shakti Upper Thatch, 31.78646° N, 077.49318° E, 2408 m, 11. IV. 2018; 1 ♂, Denga Pool, 31.78400° N, 077.44241° E, 1970 m, 26. V. 2018; 1 ♂, Kherchar, 31.79334° N, 077.56797° E, 2742 m, 30. V. 2018; 3 ♂♂, Riush Thatch, 31.78602° N, 077.49277° E, 2425 m, 6. VI. 2018; 2 ♂♂, Thihnhi, 31.72932° N, 077.36654° E, 3226 m, 21. VI. 2018, leg. K. Mallick & Team.

3 ♂♂, Uttarakhand, Dist. Uttarkashi, Govind Wildlife Sanctury, Changsil, 31.1256° N, 078.0281° E, 2600 m, 12. V. 2011; 3 ♂♂, Changsil, 31.12683° N, 078.04277° E, 2200 m, 23. VII. 2012; 1 ♂, Taluka, 31.07102° N, 079.27039° E, 2400 m, 10. VI. 2012; 2 ♂♂, Taluka, 31.06753° N, 078.25186° E, 2600 m, 12. VI. 2012; 1 ♂, Taluka, 31.06264° N, 078.2635° E, 2800 m, 14. VI. 2012; 1 ♂, Taluka, 31.04347° N, 078.26258° E, 3447 m, 17. VII. 2012, leg. A. K. Sanyal.

*Description*. Length of forewing: Male: 23–25 mm.

In wing morphology *P*. *debilis indica* can be differentiated from the nominotypical subspecies in following features: generally larger size, average male forewing length 23–25 mm; forewing ground colour darker vinous-brown, irrorated with fuscous; hindwing with slightly wavy, straight medial line almost touching the discal spot and immediately followed by a crenulated postmedial line prominent up to CuA_1_; in hindwing underside postmedial line is represented only by black specks in each vein.

*Distribution*. **India:** Himachal Pradesh (Kangra, Kullu), Uttarakhand (Uttarkashi) [6]; **Global:** Nepal (Mahakali) [48].

*Bionomy*. Unlike its nominotypical subspecies, this subspecies is distributed eastward, in North-Western and Western Himalaya, preferably within the much lower altitudinal belt of 2000 m to 3000 m with mean individual abundance at 2650 m. The subspecies was recorded between wide annual mean temperature range of 8°C to 20°C, with mean abundance at 14.5°C. It occurs in habitats with 1000 mm to 1300 mm of annual precipitation. The species was active during the monsoon months of May to July in wide types of habitats like moist temperate deciduous forest, mixed coniferous forest, upper oak-fir forest, subalpine and alpine dwarf *Rhododendron* scrubs (S9 Fig).

#### *Psyra falcipennis* Yazaki, 1994

(Figs 5L, 6F, 7F, 9D and 10G–10L).

*Psyra falcipennis* Yazaki, [22]: 32, pl. 71, figs 11, 14; text-figs 374, 380. Holotype ♂, Nepal: Mt. Phulchouki (NSMT).

*Psyra falcipennis*: [15]: 470.

*Material examined*. INDIA: 1 ♂, Sikkim, Dist. West Sikkim, Khangchendzonga Biosphere Reserve, Yoksum, 27.37864° N, 088.22087° E, 1879 m, 19. XI. 2019; 1 ♀, 23. XI. 2019; 1 ♀, 24. XI. 2019; 1 ♀, Khoyngtey, 27.37947° N, 088.22678° E, 1950 m, 27. XI. 2019; 1 ♀, Dist. North Sikkim, Khangchendzonga Biosphere Reserve, Rabum, 27.65842° N, 088.60463° E, 2000 m, 13. XII. 2019, leg. A. K. Sanyal & Team.

1 ♀, Arunachal Pradesh, Dist. Dibang Valley, Dihang Dibang Biosphere Reserve, Chigupani, 29.05741° N, 095.01435° E, 1848 m, 18. IV. 2017, leg. S. Gayen & Team.

*Description*. Length of forewing: Male: 22 mm; Female: 24–27 mm.

Head, collar, thorax and patagia olivaceous-brown; palpi dark brown with ochreous tips; antennae ochreous dorsally, dark brown beneath; metathorax with two black dots; abdomen olivaceous dorsally, ventrally ochreous with brown irroration; fore and mid tibia brown, hind tibia ochreous with brown striation.

Forewing olivaceous-brown with black irroration; apex strongly falcate especially in females; costa ochreous till middle from the base, then dark olive, striated sparsely with black throughout; a single, distinct, round, brown discal spot; antemedial line indistinct, highly crenulated, suffused with fuscous on its inner side; postmedial area having two indistinct lines not forming any clear band, approaching each other at inner margin, inner line excurved below costa, the outer line formed by black vein dots and the area between these two lines slightly tinged with fulvous; an oblique, elongated patch, densely irrorated with black, from apex to M_3_, appearing as a dark apical streak in the submarginal area, inside which and disconnected from it there are two irregular, black patches, outlined with rufous between M_3_ and M_1_; series of three black spots from inner margin up to CuA_2_ just beyond the postmedial line; a marginal series of black specks; cilia golden-yellow.

Hindwing pale ochreous with olive tinge and black irroration; a large, brown discal spot immediately followed by straightish, black, distinct medial line; the postmedial line formed by black vein dots; the area between these two lines filled in with fulvous; traces of a very indistinct submarginal line; a marginal series of black specks, cilia golden-brown.

Forewing underside olivaceous-brown, tinged with fulvous, irrorated with black except for medial area; a prominent, black discal spot to both wings; submarginal line moderately crenulated followed by a broad submarginal dark, fuscous band; hindwing pale ochreous, irrorated with black, denser towards base; distinct straight medial line; the submarginal line formed of a row of black vein dots; submarginal dark suffusion; both wings with margin clear with series of black specks.

Uncus short, broad and roundish at the base, with triangular projection at the middle, tip apically narrow, blunt and having numerous minute scobination; gnathos apically broad, less sclerotized; valvae long, basally broad, gradually narrow towards the apex, then sharply upturned at the tip giving the apex a truncate appearance; costal margin with a medial triangular projection; costal basal process slender, outwardly curved; saccus U-shaped; juxta less sclerotized, diamond-shaped with a small, apical medial projection; aedeagus with two conical spines on a sclerotized patch on manica; vesica without any bundle of spines; an apical slender cornutus.

Papilla analis oval; apophyses anterior half the length of apophyses posterior; antrum long, broad; ductus bursae coiled, wrinkled and sclerotized; corpus bursae ellipsoid, moderately large, bearing a large oval-square signum with multiple short marginal spines and very few minute central spines.

*Diagnosis*. The species is typically characterized by its olive ground colour of the forewing and two small, black, triangular spots between M_3_ and M_1_, disconnected from and inside the apical streak. In the male genitalia, *P*. *falcipennis* is typically characterized by the triangular costal medial projection of the valvae and the truncate appearance of valvae apex; the aedeagus with a sclerotized plate having two minute spines in the manica. In the female genitalia, ductus bursae coiled at the middle which is unique to this species.

*Distribution*. **India:** Sikkim (West, North), Arunachal Pradesh (Dibang Valley); **Global:** Nepal (Godavari), China (Shaanxi, Gansu, Zhejiang, Hubei, Hunan, Fujian, Guangxi, Sichuan, Yunnan) [15, 22]. ***The species is being reported here as new to India*.**

*Bionomy*. Being described from Nepal Himalaya in 1994, the species was hitherto unrecorded from the Indian Himalaya before this study. Currently, it is known from both Central and Eastern Himalaya, typically in East Himalayan wet temperate forest between a very narrow range of altitude of 1850 m to 2000 m, though its range extends from 900 m to 2200 m, and very rarely up to 2700 m. Wide annual mean temperature range of 6°C to 18°C characterizes the species habitat, with mean abundance recorded at around 12.5°C. 700–2300 mm annual precipitation typifies the species presence, though mean abundance was observed at 1500 mm. Individuals were mainly detected in the post-monsoon months of November–December in Central Himalaya, whereas during April in the eastern zone (S10 Fig).

#### *Psyra variablils* Mallick, Bandyopadhyay & Sanyal sp. nov.

**Fig 11:**
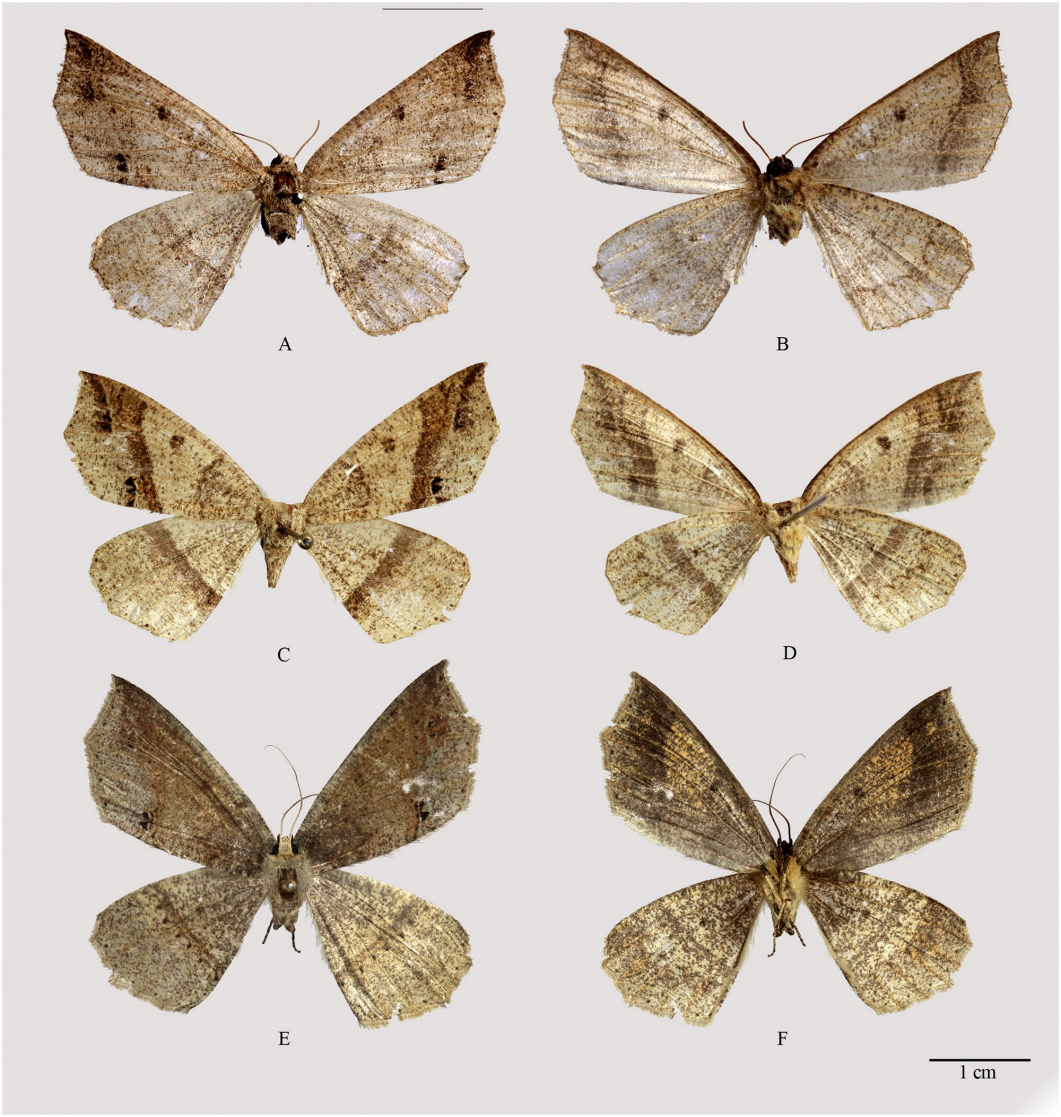
Habitus of *Psyra variabilis*. A. Holotype of *P*. *variabilis* ♂ B. Holotype of *P*. *variabilis* ♂ underside C. Paratype of *P*. *variabilis* ♂ from Uttarakhand D. Paratype of *P*. *variabilis* ♂ underside from Uttarakhand E. Paratype of *P*. *variabilis* ♂ from Sikkim F. Paratype of *P*. *variabilis* ♂ underside from Sikkim.

**Fig 12:**
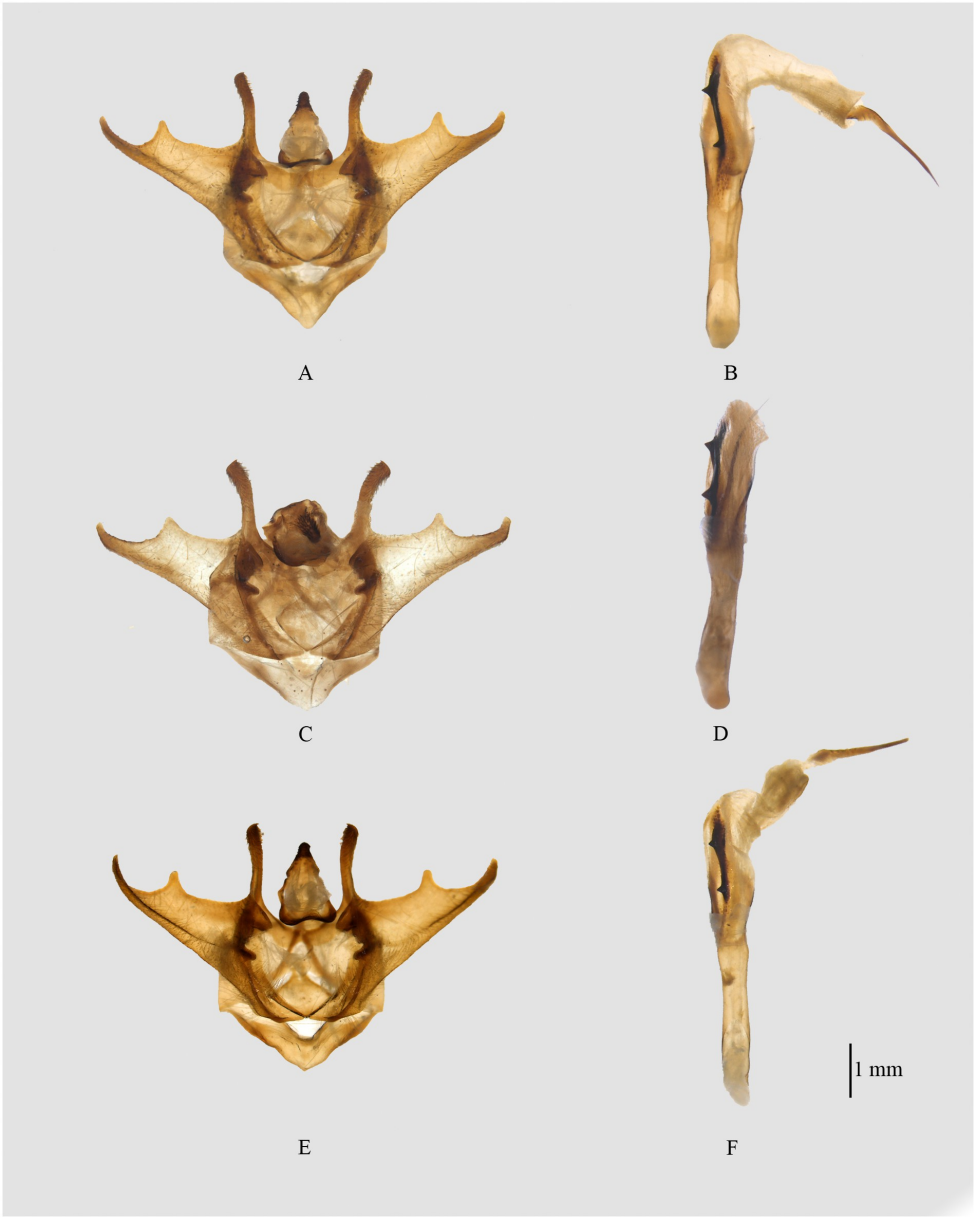
Male genitalia and aedeagus of *Psyra variabilis sp nov*. A. Ventral view of Holotype from Uttarakhand B. Aedeagus of Holotype C. Ventral view of Paratype from Uttarakhand D. Aedeagus of paratype from Uttarakhand E. Ventral view of Paratype from Sikkim F. Aedeagus of paratype from Sikkim.

urn:lsid:zoobank.org:act:F60298E5-0EEE-4D95-82C2-84B38A6459B8 (Figs 11A–11F and 12A–12F).

*Material examined*. **Holotype:** ♂, INDIA: Uttarakhand, Dist. Uttarkashi, Govind Wildlife Sanctuary, Changsil, 31.12519° N, 078.03283° E, 2400 m, 10. VII. 2012, leg. A. K. Sanyal. **Paratypes:** 2 ♂♂, same data as holotype.

2 ♂♂, Sikkim, Dist. West Sikkim, Khangchendzonga Biosphere Reserve, Yoksum, 27.37864° N, 088.22087° E, 1879 m, 19. XI. 2019; 1 ♂, Lingthem, 27.52537° N, 088.49294° E, 1496 m, 11. XII. 2019, leg. A. K. Sanyal & Team.

*Description*. Length of forewing: Male: 22–24 mm.

Vertex, clypeus and patagia ochreous, irrorated with olive scales; collar brownish-olivaceous; antennae filiform, straw-coloured dorsally, ventrally brown with flagellum black-streaked, scape slightly thick and covered with yellowish-white scales; frons yellowish-white; labial palpi dark brown, sides and tip golden-ochreous; metathorax with two black dots; abdomen olivaceous dorsally, strongly irrorated with fuscous, ventral side pale ochreous with slight fuscous irroration; legs ochreous, irrorated with violaceous-brown; foreleg pale ochreous, streaked with black, joints and upper side covered with black scales, single tibial spur covered with long yellowish hairs; midleg having a pair of unequal tibial spurs.

Forewing strongly falcate with acute apex with outer margin strongly angulated at M_3_; ground colour varies from light brownish-ochreous with contrasting dark olive-brown elements to entirely suffused with olivaceous brown, irrorated with fuscous-brown; costa golden-ochreous, irrorated with black; basal area thickly irrorated with dark up to the antemedial line which is highly crenulated outwardly with strong teeth in alternate veins; discal spot roundish and dark brown; postmedial band wider towards costa and becoming narrower towards the inner margin, formed of double lines, outer one slightly crenulated, the area in between them suffused with fulvous-brown; in submarginal area, an oblique, fuscous-brown apical streak up to M_3_, on which there are two triangular black patches between veins M_3_ and M_1_; a series of three black triangular spots from below CuA_2_ up to inner margin, just after postmedial band; a marginal series of black specks; cilia golden-brown.

Hindwing ground colour pale ochreous with dense fuscous irroration towards the base and marginal area; a dark discal spot; a prominent, fulvous-brown medial band, sometimes represented by two distinct lines only, the first one being almost straight followed by series of black vein dots, without any brown suffusion between them; a series of irregular black specks at the outer margin; a faint black cilial line; cilia golden-ochreous basally, with darker outer half.

Underside pale ochreous with sparse to dense fuscous irroration; costa suffused and striated with black; in forewing impression of an antemedial line, double postmedial line with the area between them smudged with fuscous; indistinct, oblique submarginal line; hindwing with either medial band or distinct double medial lines as in upper side; a highly crenulated submarginal line, sometimes with darker suffusion beyond them; both wings with prominent discal spot, fine, black marginal line and marginal series of black specks.

*Male Genitalia*. Uncus beak-shaped, short, basally broad, triangular, with an apically narrow and roundish tip having minute scobination; gnathos apically broad, bilobed, sclerotized; valvae long, broad at the base, apex narrow and upwardly curved; costal margin with a large, triangular, thumb-like projection situated slightly beyond the middle; the basal half of costal margin up to the medial projection shallowly concave, while the outer half from medial projection to valvae apex deeply concave; costal basal process slender, slightly outwardly curved with a hook-like tip, its length exceeds well beyond uncus; saccus roughly triangular or V-shaped with irregular outer margin and acute tip; juxta less sclerotized, diamond-shaped; aedeagus slender with two minute conical spines on a sclerotized patch on apical half; vesica long, without any bundle of spines; a long, stout apical cornutus.

*Diagnosis*. Wing pattern is very similar to *P*. *falcipennis* and *P*. *rufolineria* but the outer line of the double postmedial band in forewing in these two species are marked as series of vein dots only, while in *P*. *variabilis*, this line is slightly crenulated outwardly. The double postmedial lines in hindwing are very close to each other in *P*. *variabilis* compared to other two species. The most distinctive feature of the new species lies in the shape and position of the two submarginal black spots which are distinctly triangular, not outlined with rufous and positioned on the apical streak in *P*. *variabilis*, while those in *P*. *falcipennis* are irregular in shape, outlined with rufous and inside and disconnected from the apical streak.

In the male genitalia, uncus and triangular process on costa of valvae as in *P*. *falcipennis*, but the tip of valvae is pointed, inwardly curved in *P*. *variabilis*, while it is truncate in *P*. *falcipennis*; the base of the uncus is pitcher-shaped in *falcipennis*, while they are with a slight concavity in the new species; saccus in *P*. *variabilis* is V-shaped with pointed tip while that in *falcipennis* is U-shaped with rounder and blunt tip; costal basal process exceeds uncus and have a hook-like tip in the new species while that in *falcipennis* doesn’t exceed uncus and without any apical hook.

Female unknown.

*Distribution*. **India:** Uttarakhand (Uttarkashi), Sikkim (West).

*Bionomy*. The new species is being described from the Western Himalayan landscape of Uttarakhand in moist temperate deciduous forest habitat and Central Himalaya of Sikkim in wet temperate forest. The species habitat is characterized by 1700 m to 2400 m altitudinal range, 11°C to 17°C annual mean temperature and 1300 mm to 2400 mm annual mean precipitation. The individuals were captured during the months of July and November–December when the night time temperature was between 9–14°C with very high relative humidity of 95–96% (S11 Fig).

*Etymology*. New species name refers to the fact that individuals of the species are extremely variable in forewing colour pattern and markings, not only from different landscapes but also within the same population, while their genitalia characters are uniform.

#### *Psyra crypta* Yazaki, 1994

**Fig 13:**
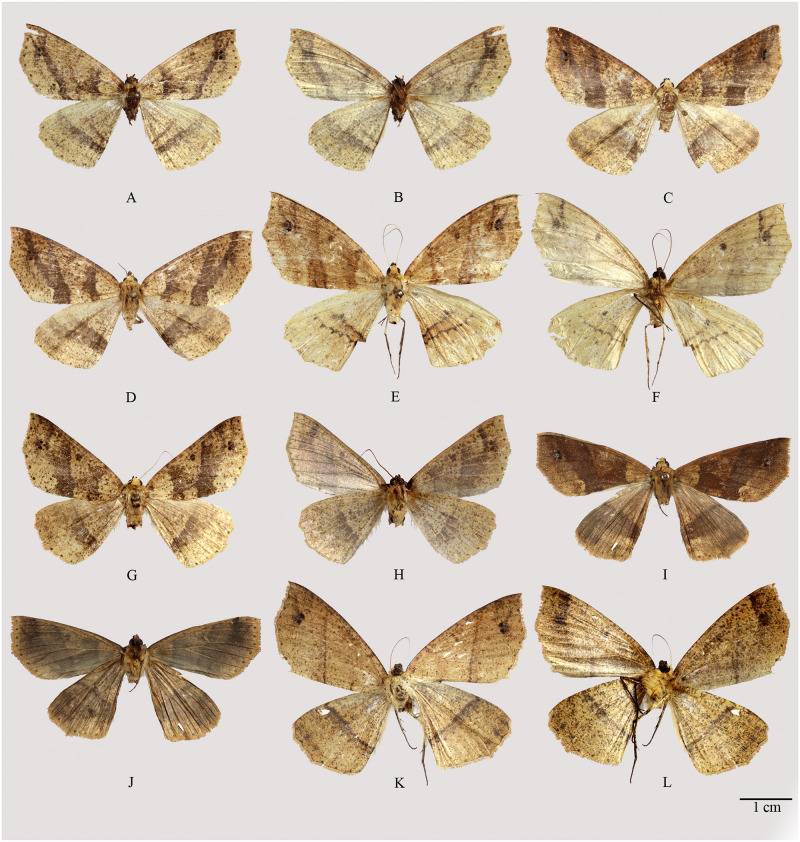
Habitus of *Psyra crypta*, *P*. *spurcataria*. A. *P*. *crypta* ♂ B. *P*. *crypta* ♂ underside C. *P*. *crypta* ♂ D. *P*. *crypta* ♂ E. *P*. *crypta* ♀ F. *P*. *crypta* ♀ underside G. *P*. *spurcataria* ♂ H. *P*. *spurcataria* ♂ underside I. *P*. *spurcataria* ♂ J. *P*. *spurcataria* ♂ underside K. *P*. *spurcataria* ♀ L. *P*. *spurcataria* ♀ underside.

**Fig 14:**
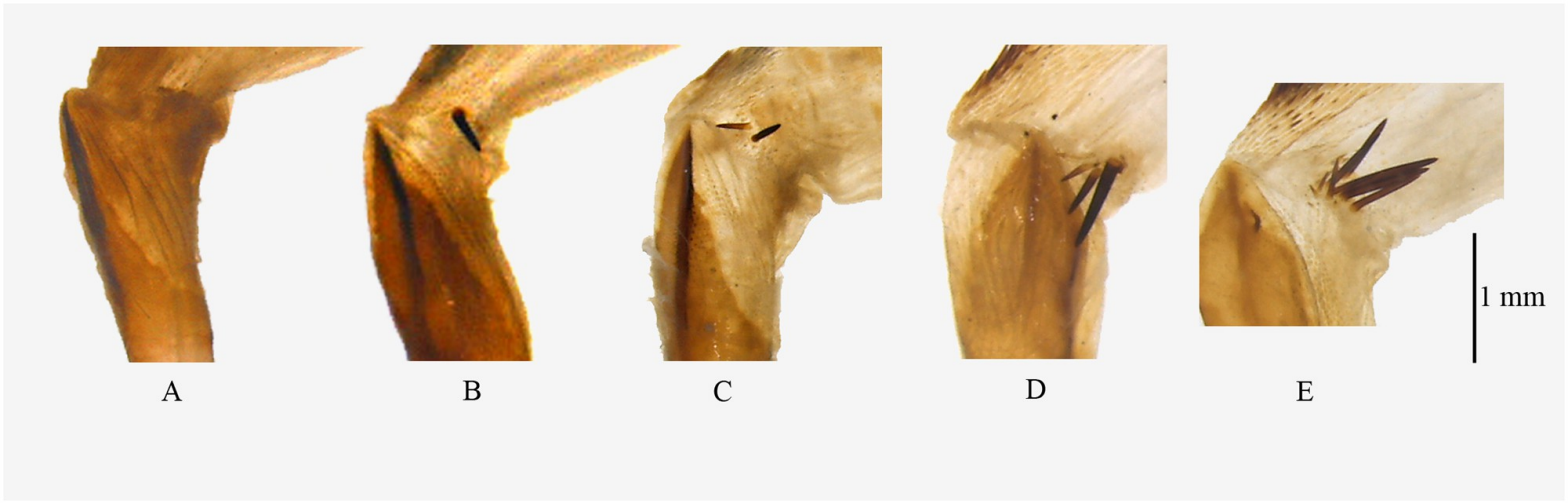
Variation of Basal spine of vesica in aedeagus in *Psyra crypta*. A. Absence of spine B. A single spine C. Double spine D. Triple spine E. Multiple spine.

(Figs 5H, 6G, 7G, 9E, 13A–13F and 14A–14E).

*Psyra crypta* Yazaki [22]: 26, pl. 71, figs 10, 13, 16; text-figs 373, 379. Holotype ♂, Nepal: Mt. Phulchouki (NSMT).

*Material examined*. INDIA: 1 ♀, Himachal Pradesh, Dist. Kullu, Great Himalayan National Park, Denga Pool, 31.784° N, 077.44241° E, 1970 m, 24. IX. 2019, leg. K. Mallick & Team.

1 ♀, Uttarakhand, Dist. Pithoragarh, Askot Wildlife Sanctuary, 29.91536° N, 080.4032° E, 2314 m, 22. IX. 2016; 2 ♂♂, Ringal Forest, 29.92458° N, 080.39169° E, 2932 m, 25. IX. 2016; 1 ♂, Ringal Forest, 29.92378° N, 080.393° E, 2800 m, 20. VI. 2018; 3 ♂♂, Jimjhini, 29.92121° N, 080.39719° E, 2627 m, 18. VI. 2018, leg. A. K. Sanyal & Team; 1 ♀, Kanar, 29.90064° N, 080.39195° E, 1675 m, 30. X. 2019, leg. U. Bandyopadhyay.

1 ♂, 1 ♀, Sikkim, Dist. West Sikkim, Khangchendzonga Biosphere Reserve, Yoksum, 27.37864° N, 088.22087° E, 1879 m, 19. XI. 2019; 1 ♀, 20. XI. 2019; 1 ♀, 26. XI. 2019; 1 ♀, Khoyngtey, 27.37947° N, 088.22678° E, 1950 m, 28. XI. 2019; 1 ♀, Dist. North Sikkim, Khangchendzonga Biosphere Reserve, Rabum, 27.65842° N, 088.60463° E, 2000 m, 13. XII. 2019, leg. A. K. Sanyal & Team.

1 ♀, West Bengal, Dist. Darjeeling, Singalila National Park, Manedara, 27.11471° N, 088.1° E, 2168 m, 20. X. 2019; 1 ♂, 28. X. 2019; 2 ♂♂, 29. X. 2019; 1 ♀, 1. XI. 2019; 1 ♂, Khopidara, 27.00525° N, 088.1189° E, 2054 m, 6. XI. 2018, leg. A. K. Sanyal & Team.

1 ♂, Arunachal Pradesh, Dist. Dibang Valley, Dihang Dibang Biosphere Reserve, Etabe, 28.80089° N, 095.95936° E, 2273 m, 06. IV. 2018, leg. S. Gayen & Team.

*Description*. Length of forewing: Male: 21–23 mm; Female: 25–27 mm.

Head, clypeus, thorax reddish-ochreous, collar and frons darker, palpi dark brown; antennae filiform, ochreous dorsally and ventrally brown; thorax irregularly covered with pale yellow scales and brown irroration; metathorax with two black dots; fore tibia, mid tibia and hind tibia ochreous, striated with blackish-brown dorsally; hind tibia of male dilated with bright ochreous hair pencil; abdomen ochreous, strongly suffused with brown dorsally.

Forewing ground colour pale reddish-brown with fuscous and fulvous irroration; costa red-brown, streaked with black throughout; basal area without much striation; antemedial band wavy, fuscous or reddish-brown; discal spot dark with whitish centre; postmedial band lighter towards costa, formed of two straightish lines which are parallel to each other towards inner margin and diverging towards costa, inner line curved inwards up to CuA_1_, then reaches the inner margin almost at right angle; outer line slightly wavy, little excurved below costa and incurved up to CuA_1_, then slightly excurved up to inner margin, the area between these two lines strongly suffused with dark fuscous, reddish-brown or olivaceous-brown; submarginal line originating at costa and continues as distinctly wavy up to M_1_, then gradually becoming indistinct and more or less straight as it reaches the inner margin; submarginal and postmedial lines come in close proximity between M_1_ and M_3_ where two prominent, conjoined black spots present which are surrounded by whitish scales; medial and postmedial band sometimes conjoined near inner margin to form a single broad band; marginal series of white-centred black specks; females generally much paler and suffused with fulvous instead of fuscous; bands also much lighter; cilia ochreous.

Hindwing ground colour same as forewing; the medial brown band is divided into two well separated lines, inner one straight, visible up to median nervure, very close to discal dot, often touching it; outer one almost complete, crenulated and with dark suffusion beyond it; traces of a submarginal brown line; in female, ground colour much lighter without any fuscous suffusion; the medial band is replaced by two clear dark lines, the area between them is filled in with fulvous; marginal series of dark specks.

Underside more reddish, strongly irrorated in male; prominent discal dot; antemedial, postmedial and submarginal diffused dark bands; females with prominent discal dots in both wings and traces of postmedial and submarginal blackish lines prominent only towards costa with two black spots between M_1_ and M_3_; hindwing with two prominent medial lines and postmedial series of blackish vein dots.

Uncus short, beak-like, apically sclerotized and with finger-like projection; gnathos long, very slender, strongly sclerotized, pointed at tip; valvae broad, triangular with truncate apex; costal basal process very long, bent outwardly at middle; saccus U-shaped; juxta moderately sclerotized; aedeagus long, slender; vesica with a bunch of dense short spines and an apical, long, acute cornutus.

Papilla analis oval; apophyses anterior shorter than apophyses posterior; antrum broad, strongly sclerotized, wrinkled; ductus bursae long, wrinkled and sclerotized; corpus bursae oval, moderately large, bearing a large oval signum with multiple, short, blunt marginal spines and very few minute central spines.

*Diagnosis*. In outer appearance, *P*. *crypta* is almost identical with *P*. *spurcataria*, but can be distinguished from it by the character of hindwing medial band which is distinctly double in both upper and underside, with the outer line much crenulated in *crypta*, whereas, in *spurcataria*, the medial band is compact and without any crenulated outer line. Male genitalia is similar with *P*. *spurcataria* but uncus is broader in apical half than *P*. *spurcataria;* the medial process of gnathos is longer and stouter in *P*. *crypta*, whereas, the process is shorter, not reaching the length of uncus in *spurcataria*; costal margin of valvae in *crypta* is much more concave giving it a narrower appearance; moreover, the bend in the costal basal process is much more medial in position in *crypta* rather than more towards the tip in *spurcataria*. The apical spine or cornutus is shorter and stouter than *P*. *spurcataria;* in female genitalia, ductus bursae shorter and broader than *P*. *spurcataria* and lamella postvaginalis absent.

*Remarks*. Basal spines in the aedeagus of *P*. *crypta* may vary in numbers within and between populations, while other morphological and genitalia characters remain the same. The number of spines may be single, double or triple and sometimes even up to five large and three shorter ones (Fig 14).

*Distribution*. **India:** Himachal Pradesh (Kullu), Uttarakhand (Chamoli, Pithoragarh), Sikkim (West, North), West Bengal (Darjeeling), Arunachal Pradesh (Dibang Valley) [23]; **Global:** Nepal (Kosi, Janakpur, Godavari) [22].

*Bionomy*. The species is very widely distributed from North-Western to Eastern Himalaya with mean abundance at an altitude of 2200 m while most of the individuals were recorded between 1900–2600 m, though the species range extends nearly between 1600 m to 3000 m. Mean abundance was recorded at an annual mean temperature of 14°C, whereas most of the individuals were recorded between 11°C to 16°C. The annual precipitation range of the species was between 1200 mm and 2900 mm, while maximum abundance was observed between 1700 mm to 2300 mm, with mean abundance at 1850 mm. Species was rarely active during Pre-monsoon in April to June while maximum population bloom was observed in the post-monsoon season of October–November. In Western Himalaya, the species was mainly active in various Oak forests like Banj (*Quercus leucotrichophora*), Rianj (*Q*. *lanuginosa*) and Kharsu (*Q*. *semicarpifolia*) dominated upper oak-fir forest. In Central Himalaya and Eastern Himalaya, the species mainly inhabits the wet temperate forest and mixed coniferous forest (S12 Fig).

#### *Psyra spurcataria* (Walker, 1863)

(Figs 5K, 6H, 7H, 9F and 13G–13L).

*Hyperythra spurcataria* Walker [2]: 1498. Lectotype ♂, India: Darjeeling (BMNH).

*Hyperythra spurcataria*: Moore [4]: 619.

*Hyperythra spurcataria*: Cotes and Swinhoe [46]**:** 478.

*Psyra spurcataria*: Butler, 1889 [6]: 20.

*Orbasia spurcataria*: Swinhoe [43]: 222.

*Psyra spurcataria*: Hampson, 1895 [8]: 221.

*Zethenia florida* Bastelberger, 1911 [13]: 22. Lectotype ♂, China: Formosa [Taiwan]: Arijan (DEI).

*Psyra spurcataria*: Prout, 1927 [49]: 795.

*Psyra spurcataria*: Yazaki [21]: 35.

*Psyra spurcataria*: [15]: 469.

*Material examined*. INDIA: 1 ♂, Himachal Pradesh, Dist. Kangra, Dharamshala, Kotwali Bazar, 32.21659° N, 076.31811° E, 1362 m, 16. X. 2018, leg. A. Raha & Team; 1 ♀, Dist. Kullu, Great Himalayan National Park, Vred Nala, 31.77133° N, 077.47926° E, 2800 m, 08. VI. 2018, leg. K. Mallick & Team.

1 ♀, Uttarakhand, Dist. Pithoragarh, Askot Wildlife Sanctuary, Chilamdhar, 30.13751° N, 080.24781° E, 1714 m, 09. X. 2017; 2 ♂♂, Gowalghat, 29.91398° N, 080.40338° E, 2248 m, 14. VI. 2018, leg. A. K. Sanyal & Team; 1 ♂, Sarmoli, 30.0769° N, 080.23568° E, 2195 m, 18. X. 2019, leg. U. Bandyopadhyay.

1 ♂, 2 ♀♀, Sikkim, Dist. West Sikkim, Khangchendzonga Biosphere Reserve, Yoksum, 27.37864° N, 088.22087° E, 1879 m, 19. XI. 2019; 1 ♂, 1 ♀, 20. XI. 2019; 1 ♂, 1 ♀, 22. XI. 2019; 2 ♂♂, 23. XI. 2019; 1 ♂, Khoyngtey, 27.37947° N, 088.22678° E, 1950 m, 28. XI. 2019; Dist. North Sikkim, Khangchendzonga Biosphere Reserve, Rabum, 27.65842° N, 088.60463° E, 2000 m, 13. XII. 2019, leg. A. K. Sanyal & Team.

1 ♂, West Bengal, Dist. Darjeeling, Singalila National Park, Chitrey, 26.9913° N, 088.1119° E, 2366 m, 26. VIII. 2016; 1 ♀, Meghma, 27.0327° N, 088.08314° E, 2971 m, 19. V. 2018, leg. K. Bhattacharyya & Team; 1 ♀, Manedara, 27.11471° N, 088.1° E, 2168 m, 22. X. 2018; 1 ♀, 28. X. 2018; 1 ♂, 1 ♀, 01. XI. 2018, leg. A. K. Sanyal & Team; 1 ♀, Dist. Kalimpong, Neora Valley National Park, Suntale Khola, 27.01042° N, 088.78983° E, 760 m, 27. XI. 2017, leg. P. Chatterjee & Team; 1 ♂, Chaudapheri, 27.0863° N, 088.6596° E, 2600 m, 12. VII. 2018, leg. K. Bhattacharyya & Team.

1 ♀, Arunachal Pradesh, Dist. Dibang Valley, Dihang Dibang Biosphere Reserve, Mippi, 28.96423° N, 095.808200° E, 1554 m, 31. X. 2017; 1 ♀, Anini, 27.01042° N, 088.78983° E, 760 m, 27. XI. 2017, leg. S. Gayen & Team.

*Description*. Length of forewing: Male: 22–23 mm; Female: 24–27 mm.

Head, thorax, patagia ochreous; collar darker; palpi dark brown; antennae dorsally ochreous, brown beneath; metathorax with two dark dots; abdomen ochreous, strongly suffused with fuscous dorsally; legs dark violaceous-brown, hind tibia with ochreous hair pencil.

Forewing ground colour ochreous to reddish-brown with fuscous and fulvous irroration; costa dark rufous with dense fuscous irroration; basal area clear without much irroration; in males, antemedial band fuscous-brown, broadest at middle and obsolescent towards costa, in female, traces of an indistinct antemedial line instead of band; discoidal spot distinct, large, with whitish centre in males, obsolescent or just a faint streak in females; postmedial band broad and as Y-like appearance towards costa, slightly incurved, becoming slightly excurved below CuA_2_ up to inner margin; in female, the postmedial band is represented only by two separate lines, inner one almost straight, the outer one represented by series of vein dots, almost coalescing with the inner one towards inner margin; a brown oblique apical streak up to M_3_ between which and postmedial band, two small, disjoined black spots present between M_3_ to M_1_; marginal series of black specks with whitish centre; marginal series of streaks instead of cilial line; cilia pale ochreous.

Hindwing ground colour same as forewing; a prominent broad, fuscous medial band, broader towards the costa; discal spot indistinct; submarginal band obsolescent, only visible from apex to vein M_1_; in females, the postmedial band is narrow and of the same width throughout; inner margin straight and thick, outer margin crenulated; marginal series of dark specks with whitish centre.

Underside ground colour paler with slight violaceous suffusion, thickly irrorated with fuscous; markings as in upper side.

Uncus triangular, slender with finger-like apical projection, tip blunt; gnathos with the short median process; valvae triangular, slender, apex blunt; costal basal process long, broad, bent outwards at two-third from base; saccus broad V-shaped; juxta diamond-shaped; aedeagus very long, slender; vesica with three bundles of minute spines and a terminal acute, long cornutus; eighth sternite with a pair of sclerotized, spine-like processes.

Papilla analis oval; apophyses anterior shorter than apophyses posterior; antrum broad, strongly sclerotized; ductus bursae long, narrow, wrinkled, moderately sclerotized forming lamella postvaginalis; corpus bursae oval, large, bearing a large round signum with multiple, short, marginal spines and few minute central spines.

*Diagnosis*. *P*. *spurcataria* is almost identical in wing pattern with *P*. *crypta* but in hindwing, the postmedial line is less serrate, slightly waved, obscure, while it is strongly serrate in *P*. *crypta*; the oblique apical streak of *P*. *spurcataria* is replaced by a submarginal discontinuous line in *P*. *crypta*; the postmedial crenulated line of hindwing underside is very prominent and complete in *P*. *crypta* but it is obsolescent and incomplete in *P*. *spurcataria*; the male genitalia is distinct from all other species of the genus by having a pair of the spine like processes in the eighth sternite; uncus narrower in apical half than *P*. *crypta*; the medial process of gnathos much shorter and not reaching the length of uncus but in *P*. *crypta*, it is longer than uncus; aedeagus with three bundles of vesica spines and one very long apical cornutus instead of one bundle of the spine in *P*. *crypta*; in female genitalia, a distinct lamella postvaginalis present which is unique to the species.

*Distribution*. **India:** Himachal Pradesh (Kangra, Kullu), Uttarakhand (Chamoli, Nainital, Pithoragarh), Sikkim (West, North), West Bengal (Darjeeling, Kalimpong), Arunachal Pradesh (Dibang Valley), Assam, Meghalaya (East Khasi) [8, 50]; **Global:** Nepal (Mechi, Janakpur, Godavari), Myanmar (Siam), China (Guangxi, Sichuan, Yunnan, Tibet), Taiwan [15, 21, 49].

*Bionomy*. The species is widely distributed from Western Himalaya to Eastern Himalaya with mean abundance at an altitude of 2150 m while most of the individuals were recorded between 1700–2700 m, though the species range extends between 750 m to 3350 m. Mean abundance was recorded at an annual mean temperature of 15.7°C, whereas individuals were recorded between 7.6°C to 21.6°C. The annual precipitation range of the species was between 800 mm and 2900 mm, while maximum abundance was recorded between 1600 mm to 2500 mm, and sometimes even up to 4700 mm. Species was active during the monsoon months of June–August and post-monsoon months of October–November. In Western Himalaya, the species was active in Chir pine forest and upper oak forest, while it was mainly active in the wet temperate forests of Central Himalaya to Eastern Himalaya (S13 Fig).

#### DNA barcoding

**Fig 15:**
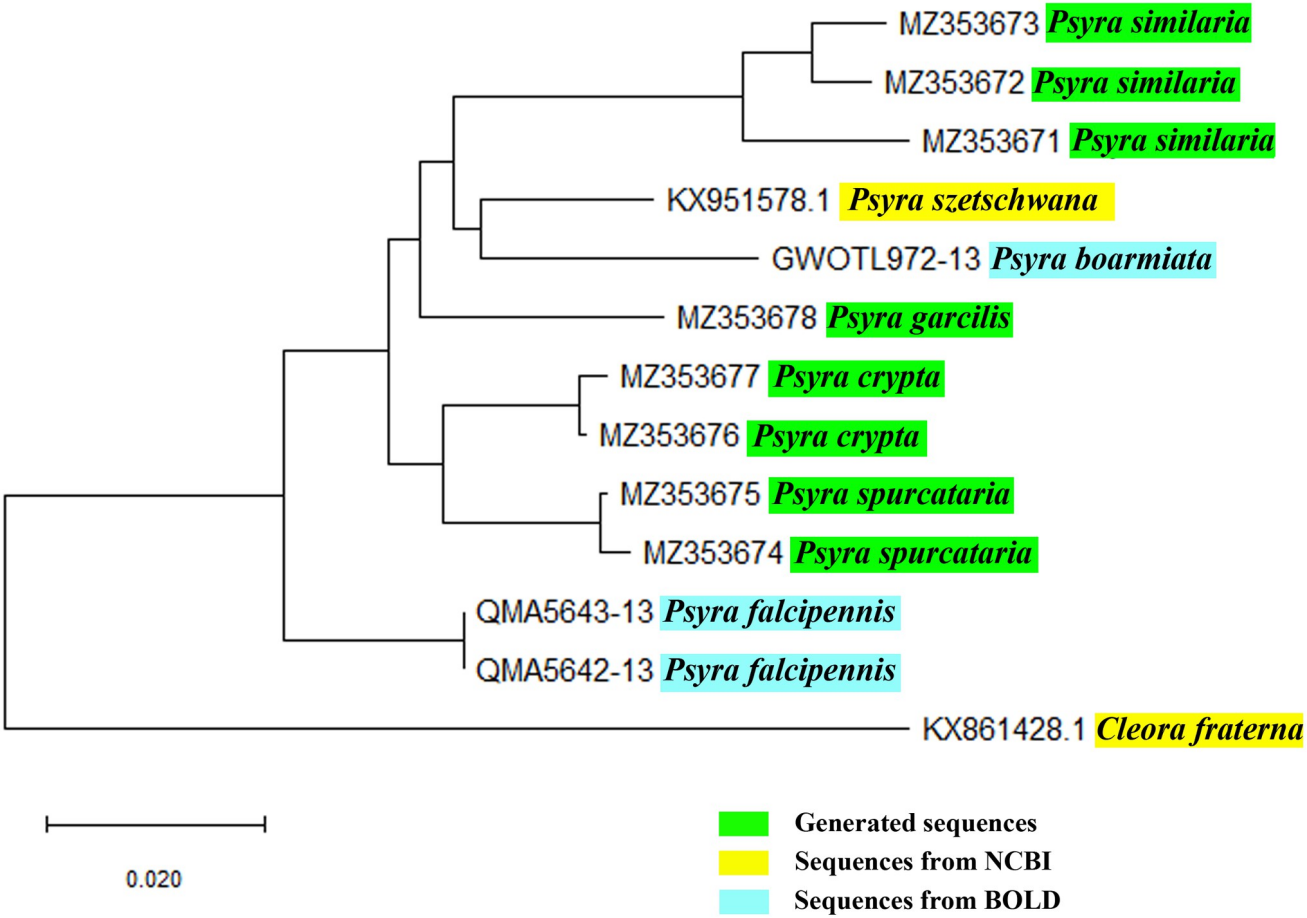
Neighbour-joining (NJ) phylogenetic tree of *Psyra* species constructed with 1,000 bootstraps of replications using Kimura-2-parameter (K2P) model. Eight DNA sequences of 4 morphologically identified species from our collection was aligned against four globally available sequences of three *Psyra* species along with *Cleora fraterna* as an outgroup, retrieved from the National Centre for Biotechnology Information (NCBI) and Barcode of Life Database (BOLD).

The 8 DNA barcodes generated in this study are accessible in NCBI with their respective accession numbers, as novel submissions of *P*. *crypta*, *P*. *similaria*, *P*. *spurcataria* and *P*. *gracilis* to the global database. As per the NJ Tree (Fig 15), cohesive clustering was seen among all the 7 species of *Psyra*. The overall mean distance in the final dataset comprising of the generated sequences aligned with all the available sequences of *Psyra* (3 species) from the global database was 5.17%. *P*. *spurcataria* formed a sister clade with *P*. *crypta* with a divergence of 3.05%. Intra-specific divergence was 0.35% for both the species. For *P*. *gracilis*, the inter-specific divergence was the highest (6.44%) with *P*. *similaria* and the lowest (4.41%) with *P*. *spurcataria*. The mean within-group divergence for *P*. *similaria* was 2.27%. Out of the 3 generated sequences for this species, the one from Eastern Himalaya (accession no MZ353671) formed a sister clade with the other two from Western Himalaya, having respective divergences of 2.3% and 3.2%. Because of this high intra-specific divergence and distant collection localities, we suspect that the Eastern Himalayan specimen might be an undescribed species/subspecies. Though we could not describe it here due to lack of enough specimens and generated barcodes, further confounded by the absence of commendable morphological differences with other *P*. *similaria* except numbers and arrangement of spines in the aedeagus vesica.

### Ecological account

Throughout the length and breadth of the Indian Himalaya, the *Psyra* species were distributed within a narrow range of the altitudinal band, mean altitudinal distribution of the genus being at 2150 m, whereas, majority of the species were distributed within 2000–2280 m. However, the range extends within 1600–2670 m, and in few exceptional cases, individual species may ascend or descend within 750–3800 m. As a typical temperate montane genus, the annual mean temperature and annual precipitation was more towards the lower range, mean temperature being at 13°C, whereas most of the species were recorded within 10.55°C to 15.7°C. Annual precipitation range for the genus was between 1200 mm to 2300 mm, with mean species distribution at 1700 mm, however, individual species inhabit areas receiving as low as 190 mm annual precipitation up to as high as 4736 mm (S19 Fig).

### Habitat preference

**Fig 16:**
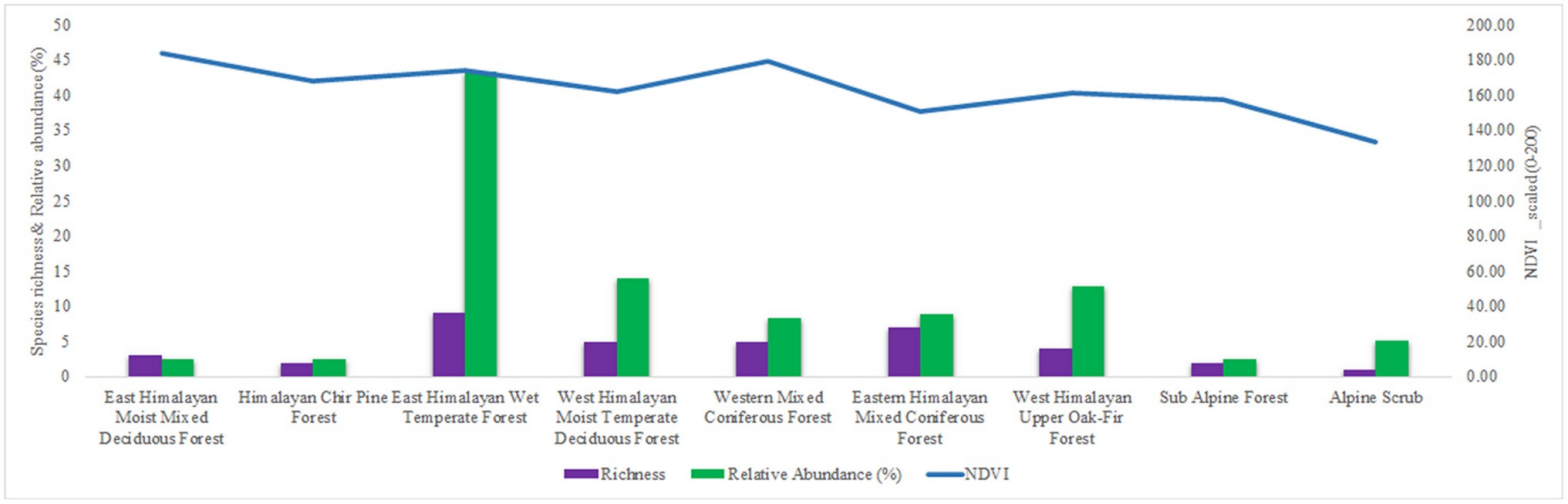
Species richness and relative abundance of *Psyra* from 9 major habitat types and their average Normalized Difference Vegetation Index (NDVI) values sampled in Indian Himalaya during sampling period of 2010–2012 and 2016–2019.

Across the whole stretch of Indian Himalaya, *Psyra* species were recorded from 19 sub-categories of forest types, which were broadly grouped into 9 major habitat types (Table 1). Among them, East Himalayan wet temperate forest harboured highest species richness with the record of 9 species having significantly high relative abundance in comparison to other habitats (Fig 16). Seven species were recorded from the East Himalayan mixed coniferous forest, but their relative abundance was very low in this habitat. Five species were encountered from West Himalayan moist temperate deciduous forest and western mixed coniferous forest with moderate abundance. Four species with relatively medium abundance were found in West Himalayan upper oak-fir forest. The two lowest altitudinal forest types of the sub-tropical zone, i.e., moist mixed deciduous forest and Chir pine forest had very low *Psyra* activity with records of 2 and 3 species respectively. Two species with very low relative abundance were observed in sub-alpine forest, whereas, only one species with high abundance was recorded from alpine scrub (Fig 16).

**Table 1:** Major habitat types in different biogeographic provinces of the Indian Himalaya with their respective altitudinal range, average Normalized Difference Vegetation Index (NDVI) values and *Psyra* species recorded during sampling period of 2010–2012 and 2016–2019.

Habitat Type	Biogeographic Province	State	Altitudinal Range (m)	Average NDVI	*Psyra* species recorded
East Himalayan moist mixed deciduous forest	Central Himalaya & Eastern Himalaya	West Bengal & Arunachal Pradesh	600–1000	181.8	*angulifera*, *gracilis*, *spurcataria*
Himalayan Chir pine forest	North-Western Himalaya & Western Himalaya	Himachal Pradesh & Uttarakhand	1300–1800	168.17	*angulifera*, *spurcataria*
East Himalayan wet temperate forest	Central Himalaya & Eastern Himalaya	West Bengal, Sikkim & Arunachal Pradesh	1300–2300	173.65	*angulifera*, *crypta*, *cuneata*, *falcipennis*, *gracilis*, *similaria*, *variabilis*, *spurcataria*, *szetschwana*
West Himalayan moist temperate deciduous forest	North-Western Himalaya & Western Himalaya	Himachal Pradesh & Uttarakhand	1900–2500	161.60	*angulifera*, *crypta*, *debilis indica*, *similaria*, *variabilis*
Western mixed coniferous forest	North-Western Himalaya & Western Himalaya	Himachal Pradesh & Uttarakhand	1600–2500	179.09	*angulifera*, *crypta*, *debilis indica*, *similaria*, *variabilis*, *spurcataria*
East Himalayan mixed coniferous forest	Central Himalaya & Eastern Himalaya	West Bengal & Arunachal Pradesh	1900–3000	150.77	*angulifera*, *crypta*, *dsagara*, *fulvaria*, *gracilis*, *similaria*, *spurcataria*
West Himalayan upper oak-fir forest	North-Western Himalaya & Western Himalaya	Himachal Pradesh & Uttarakhand	2600–3000	160.97	*crypta*, *debilis indica*, *similaria*, *spurcataria*
Sub-alpine forest	North-Western Himalaya & Western Himalaya & Eastern Himalaya	Himachal Pradesh, Uttarakhand & Arunachal Pradesh	3000–3800	157.04	*debilis indica*, *similaria*
Alpine scrub	Trans-Himalaya & Western Himalaya	Ladakh & Uttarakhand	2800–3500	133.60	*debilis debilis*, *debilis indica*

Among the total 12 species/1 subspecies recorded, 4 species, viz. *P*. *angulifera*, *P*. *similaria*, *P*. *spurcataria* and *P*. *crypta* were sampled from all the biogeographic provinces across six habitat types showing a wide range in habitat preference. Six species were narrowly specialist in their habitat choice: *P*. *cuneata*, *P*. *falcipennis* and *P*. *szetschwana* recorded only from East Himalayan wet temperate forest; *P*. *debilis debilis* recorded only from Trans-Himalayan alpine scrub; *P*. *dsagara* and *P*. *fulvaria* recorded only from East Himalayan mixed coniferous forest.

#### Climatic/Habitat suitability

The individual climatic suitability models generated for the genus *Psyra* and the selected species had AUC values above 0.8 (±0.05–0.28) and TSS scores 0.72–0.84, indicating good model predictions [39].

*Current climatic suitability of genus* Psyra. Mean temperature of the warmest quarter, precipitation of the coldest quarter, temperature seasonality and precipitation of the driest month (S14 Fig) were the major bioclimatic factors accounting for 80% cumulative influence on current distribution (S18A Fig) of the genus *Psyra* (Table 2).

**Table 2:** Area under Curve (AUC) values and True Skill Statistics (TSS) scores of MaxEnt species distribution models generated for *Psyra* genus and 3 selected taxa *P*. *angulifera*, *P*. *debilis debilis*, *P debilis indica* along with relative contribution (%) of the significant bioclimatic variables, their range and values at which the occurrence probability of the taxa was highest.

Taxa	AUC value	TSS score	Bioclimatic Variables	Contribution (%)	Range	Highest occurrence probability
*Psyra*	0.965	0.847	Mean temperature of warmest quarter (BIO10)	28.5	0.1–3°C	1.7°C
			Precipitation of coldest quarter (BIO19)	20.6	0–700 mm	290 mm
			Temperature seasonality (BIO4)	20.1	2–9%	4.3%
			Precipitation of driest month (BIO14)	11.4	0–120 mm	5 mm
*P*. *angulifera*	0.968	0.833	Annual precipitation (BIO12)	56.7	0–7000 mm	2000 mm
			Elevation (DEM)	35.9	1000–4500 m	2300 m
			Precipitation of driest month (BIO14)	3.5	2–52 mm	6 mm
*P*. *debilis debilis*	0.851	0.722	Mean temperature of wettest quarter (BIO8)	55.8	-2–0.5°C	-2 –-1.3°C
			Temperature seasonality (BIO4)	28.3	7.5–14%	11.5–14%
*P*. *debilis indica*	0.927	0.794	Precipitation of driest quarter (BIO17)	65.8	0–190 mm	175–190 mm
			Mean diurnal range (BIO2)	22.6	0.5–1.3°C	0.5–0.6°C

**Fig 17:**
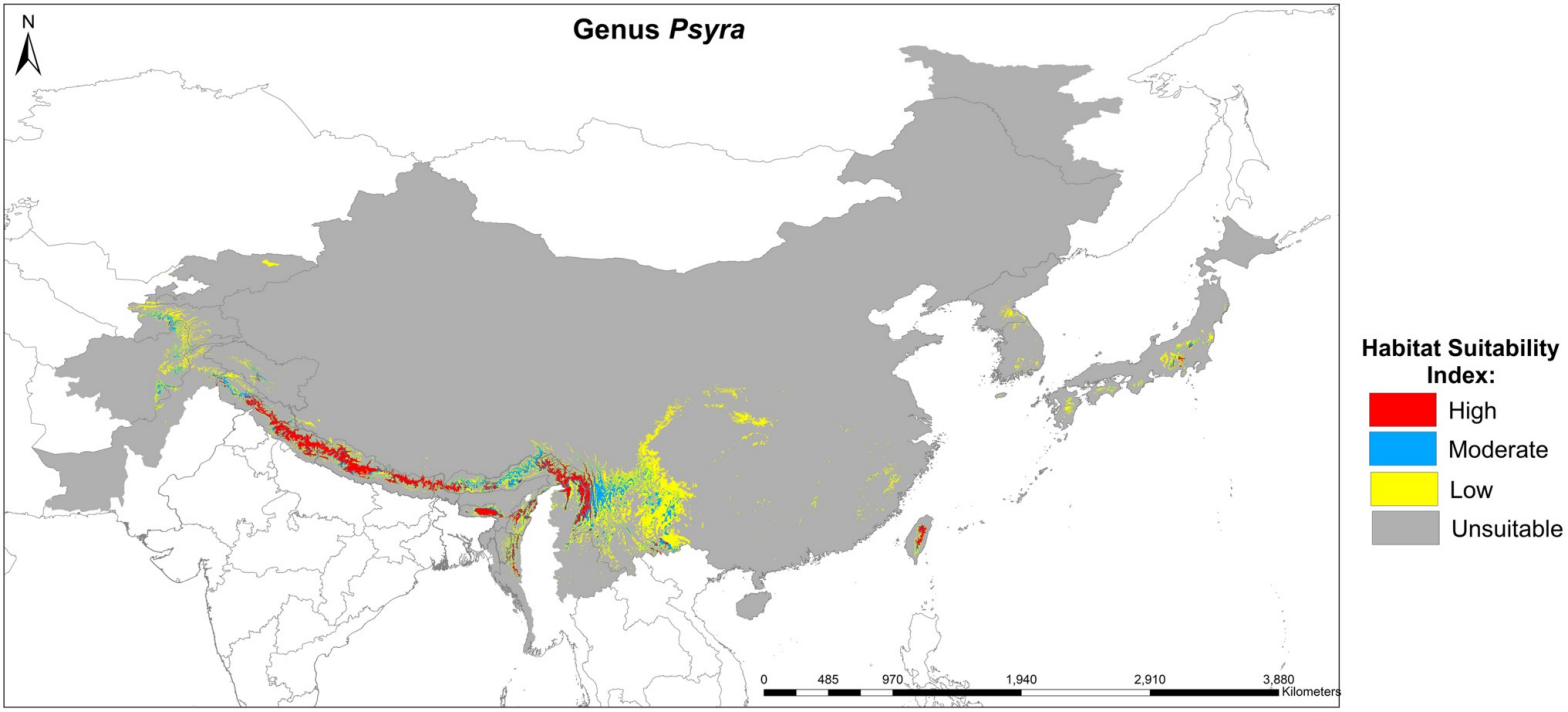
Predicted habitat suitability of the genus *Psyra* under current climatic condition (1970–2000) based on MaxEnt species distribution model.

High suitable area of 18.9 ×10^4^ sq. km under current climatic condition stretched almost throughout the Himalayan Biodiversity Hotspot (HBH) from Himachal Pradesh to western Bhutan, along with Garo-Khasi and Naga-Patkai hill ranges of North-East India, Arakan Mountain range of Myanmar, Hengduan range in the border areas of Myanmar-China, Chungyang Shan of Taiwan, Akaishi Mountains and Mount Nikko-Shirane in Japan. Additionally, 22.9 × 10^4^ sq. km of moderate suitable areas spread across the Hindu Kush range of south eastern and eastern Afghanistan, Karakoram-Pamir landscape along the border of Afghanistan and Tajikistan, Kashmir valley and Zanskar range, Dafla-Abor-Miri ranges of North-East India, Greater and Lesser Himalayan range of southern and eastern Bhutan and Hengduan range of China (Fig 17).

*Current & future climatic suitability of* Psyra angulifera. The current and predicted future distribution of *P*. *angulifera* was influenced by annual precipitation, elevation and precipitation of the driest month (S15 Fig), having 96% cumulative influence (S18B Fig) on species distribution (Table 2).

**Fig 18:**
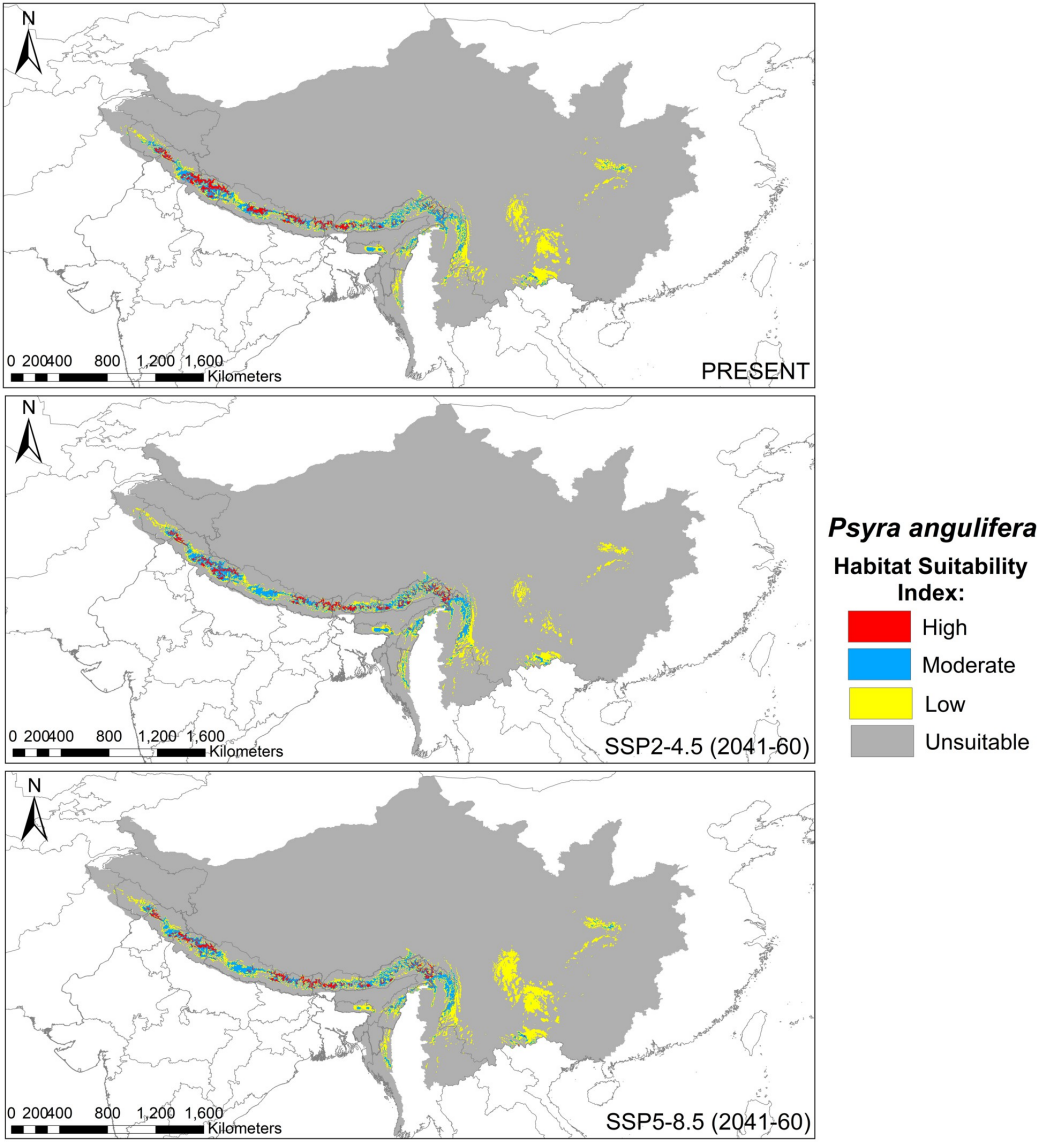
Predicted habitat suitability of *Psyra angulifera* under current (1970–2000) and two future climatic scenarios SSP2-4.5 and SSP5-8.5 in the year 2041–60 based on MaxEnt species distribution model.

In the current scenario, 46.2 × 10^3^ sq. km of the highly suitable area spread throughout HBH in disjunct patches: along the state borders of Jammu & Kashmir and Himachal Pradesh; the entire state of Uttarakhand; Western, Central and Eastern Nepal; Sikkim and northern West Bengal; western and central Bhutan; and few scattered areas in West-Kameng, Lower Subansiri, Dibang Valley and Anjaw districts of Arunachal Pradesh. In addition, 11.3 × 10^4^ sq. km of moderately suitable areas stretched continuously along HBH, Khasi Hills and Hengduan Mountain (Fig 18).

A loss of 12.5% high suitable area is predicted in the years 2041–60 under scenario SSP2-4.5, majorly in Uttarakhand and Western and Central Nepal, whereas, certain area gain is detected around the current suitable areas in Arunachal Pradesh. Under the scenario SSP5-8.5, the projected area was further reduced to 35.4 × 10^3^ sq. km with a total loss of 23.3% making the overall distribution of *Psyra angulifera* highly disjunct. In both the futuristic scenarios of SSP2-4.5 and SSP5-8.5 (Table 3), respective gain of 15.3% and 3% in moderate suitable areas are projected along Khasi and Naga-Patkai hills and Arakan-Hengduan Mountain range. Few areas are also predicted to be lost in Zanskar range and Khasi hills under SSP5-8.5 (Fig 18).

**Table 3:** The predicted suitable area (high, moderate and low) of the genus *Psyra* and selected taxa under the current climatic condition with respective area gain or loss in the year 2041–60 under two future climatic scenarios SSP2-4.5 and SSP5-8.5.

	SCENARIO	HABITAT SUITABILITY (sq. km)			HABITAT GAIN/LOSS (%)		
		High	Moderate	Low	High	Moderate	Low
**Genus *Psyra***	**Present**	189661	229899	550238			
***P*. *angulifera***	**Present**	46190.25	112691.25	220887			
	**SSP2-4.5 (2041–60)**	40439.25	129883.5	180042.75	-12.45	15.26	-18.49
	**SSP5-8.5 (2041–60)**	35417.25	116012.25	247657.5	-23.32	2.95	12.12
***P*. *debilis indica***	**Present**	16038	24482.25	57165.75			
	**SSP2-4.5 (2041–60)**	13000.5	25373.25	59656.5	-18.93	3.64	4.36
	**SSP5-8.5 (2041–60)**	6297.75	23672.25	52994.25	-60.73	-3.31	-7.30
***P*. *debilis debilis***	**Present**	5386.5	3138.75	6621.75			
	**SSP2-4.5 (2041–60)**	9375.75	3665.25	7047	74.06	16.77	6.42
	**SSP5-8.5 (2041–60)**	7674.75	2936.25	6216.75	42.48	-6.45	-6.17

*Current & future climatic suitability of* Psyra debilis debilis. With respective contributions of 55.8% and 28.3% (S18C Fig), mean temperature of wettest quarter and temperature seasonality (Table 2) are predicted to influence the current and future distribution of *Psyra debils debilis* (S16 Fig).

**Fig 19:**
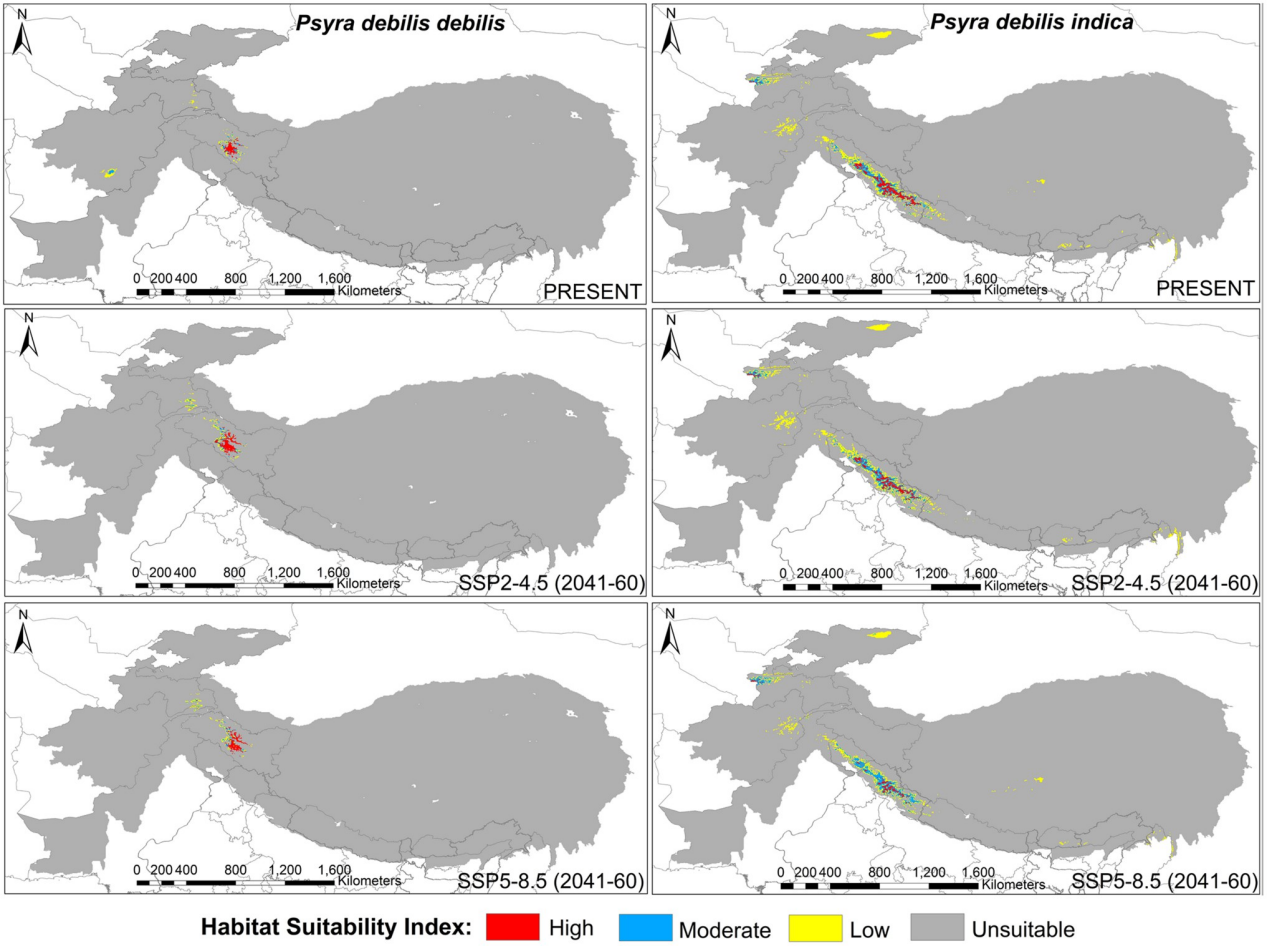
Predicted habitat suitability of *Psyra debilis debilis & P*. *debilis indica* under current (1970–2000) and two future climatic scenarios SSP2-4.5 and SSP5-8.5 in the year 2041–60 based on MaxEnt species distribution model.

The current high suitable area of 53.9 × 10^2^ sq. km concentrated in Zanskar range in Ladakh and adjoining Pakistan is projected to undergo an area gain of 74.1% and 42.5% under the scenarios SSP2-4.5 and SSP5-8.5 respectively in the years 2041–60. Rather than occupying novel areas, the majority of the gain is predicted around the present high suitable areas in Karakoram range, the occupancy thus increasing to 93.8 × 10^2^ sq. km and 76.7 × 10^2^ sq. km (Table 3) under the optimistic and pessimistic scenario respectively. A moderately suitable area around Hindu Kush range in Kandahar under the present climatic scenario is predicted to be lost completely under both the future scenarios, whereas few novel areas of moderate suitability are predicted to be gained around Pamir range in Tajikistan (Fig 19).

*Current & future climatic suitability of* Psyra debilis indica. Precipitation of driest quarter and mean diurnal range of temperature (S17 Fig) governed 90% (S18D Fig) of the distribution for this subspecies (Table 2).

Under the current scenario, high suitable areas of 16.1 × 10^3^ sq. km stretched continuously from Zanskar valley in Kashmir up till Chamoli district in Uttarakhand (Fig 19). In the years 2041–60 under scenario SSP2-4.5, 18.9% area loss is projected. Under scenario SSP5-8.5, 60.7% area is predicted to be lost making the previously continuous population broken into disjunct patches concentrated mainly in Shimla and Kullu district of Himachal Pradesh and Uttarkashi district of Uttarakhand. Current moderately suitable area of 24.5 × 10^3^ sq. km is projected to undergo 3.7% area gain under SSP2-4.5 and 3.3% area loss under SSP5-8.5 in the years 2041–2060 (Table 3).

#### Influence of major environmental predictors

**Fig 20:**
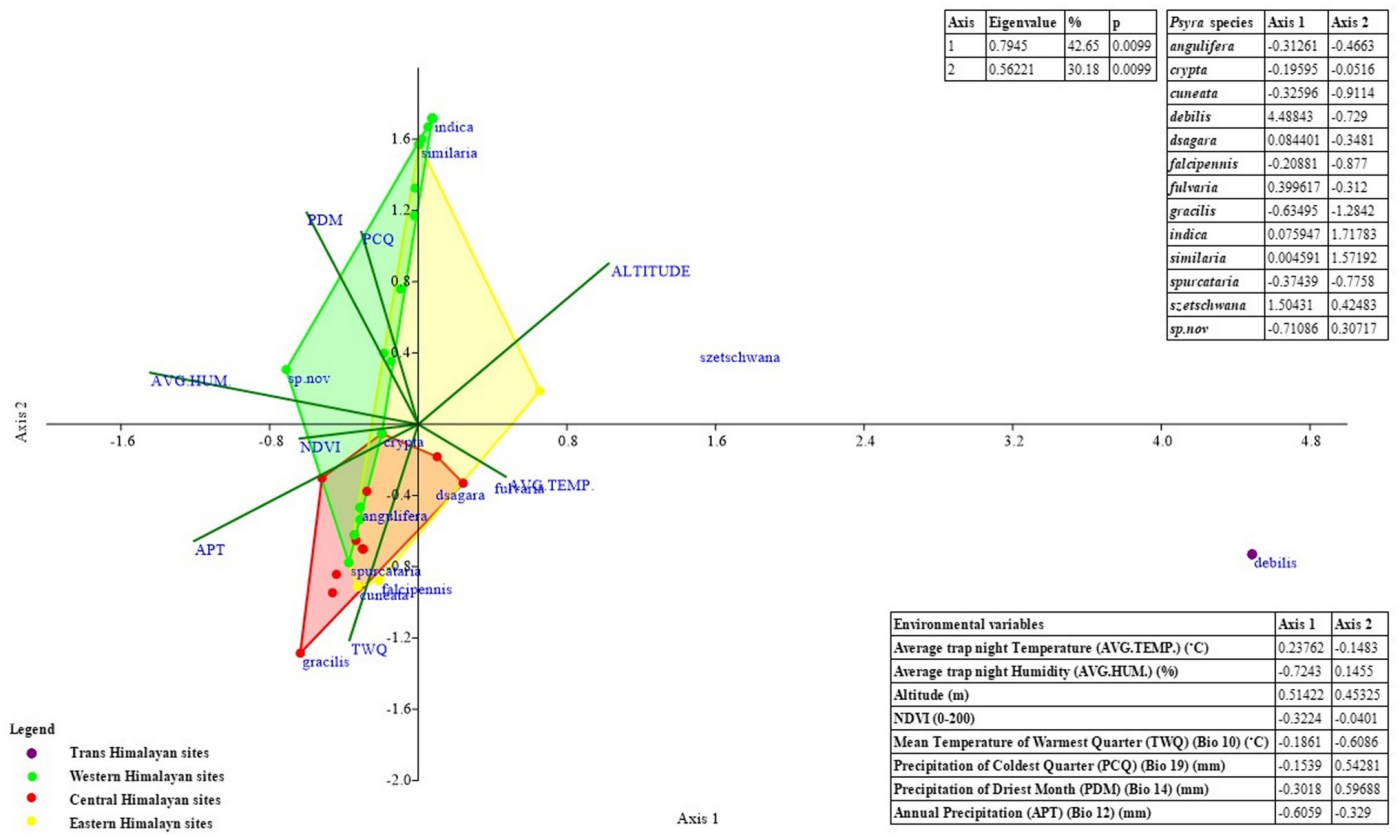
Canonical Correspondence Analysis (CCA) ordination plot of the *Psyra* species abundance against environmental predictors in 4 Himalayan zones (Trans, Western, Central & Eastern shown as coloured areas) sampled during 2010–2012 and 2016–2019. The green lines represent the extent of each environmental predictors (shown in CAPITAL) while the coloured points represent sites sampled in respective Himalayan zones. Percentage variation explained by each axis, intraset correlations between individual species, environmental predictors and ordination axes are shown in inset tables.

In the Canonical Correspondence Analysis (CCA) of environmental predictors on species abundance in 4 Himalayan zones viz. trans, western, central and eastern, the first two axes explained 72.83% cumulative variation in the data with a significance level of <0.01 (Fig 20). For abundance variation in these zones, the first CCA axis was positively influenced by altitude (r = 0.51) and average trap night temperature (r = 0.24). This axis was most negatively influenced by average trap night humidity (r = -0.72) and annual precipitation (r = -0.61). NDVI (r = -0.32), precipitation of driest month (r = -0.30), mean temperature of warmest quarter (r = -0.18) and precipitation of coldest quarter (r = -0.15) also had negative influence on axis 1. For individual species (for species score, weighted average of species abundance has been considered which is given in parentheses), the first CCA axis was highly positive for *P*. *debilis debilis* (r = 4.49), *P*. *szetschwana* (r = 1.50) and *P*. *fulvaria* (r = 0.39) which indicates a positive influence of the altitude and average trap night temperature on these species. This axis was highly negative for *P*. *variabilis* (r = -0.71), *P*. *gracilis* (r = -0.63), *P*. *spurcataria* (r = -0.37), *P*. *cuneata* (r = -0.33) and *P*. *angulifera* (r = -0.31) which indicates that abundance of these species were influenced by average trap night humidity, annual precipitation and NDVI.

The second CCA axis was positively influenced by precipitation of driest month (r = 0.60), precipitation of coldest quarter (r = 0.54) and altitude (r = 0.45), depicting strong association with *P*. *debilis indica* (r = 1.72), *P*. *similaria* (r = 1.57), *P*. *szetschwana* (r = 0.42) and *P*. *variablis* (r = 0.31). This axis was most negatively influenced by mean temperature of warmest quarter (r = -0.61) and annual precipitation (r = -0.33) and associated species were *P*. *gracilis* (r = -1.28), *P*. *cuneata* (r = -0.91), *P*. *falcipennis* (r = -0.88), *P*. *spurcataria* (r = -0.78), *P*. *debilis debilis* (r = -0.73) and *P*. *angulifera* (r = -0.47).

The CCA biplot indicated that sites sampled at Trans-Himalaya consisting of single species *P*. *debilis debilis* were uniquely positioned along the very high value of CCA axis 1, being governed mostly by altitude and average trap night temperature and showing very distant cluster from eastern, central and western Himalayan sites. The Central Himalayan sites were mainly clustered along negative values of both the axes, depicting positive effect of mean temperature of warmest quarter, annual precipitation and NDVI and strong negative influence of altitude. The Western Himalayan sites were majorly clustered between the positive value of axis 1 and negative value of axis 2, depicting the positive influence of average trap night humidity, precipitation of driest month and precipitation of coldest quarter and negative influence of average trap night temperature and altitude. The Eastern Himalayan cluster had minimum overlap with western sites and was positioned between the positive values of both the axes, depicting the positive influence of altitude and average trap night temperature but negative influence of average trap night humidity and NDVI.

#### Seasonal abundance

**Fig 21:**
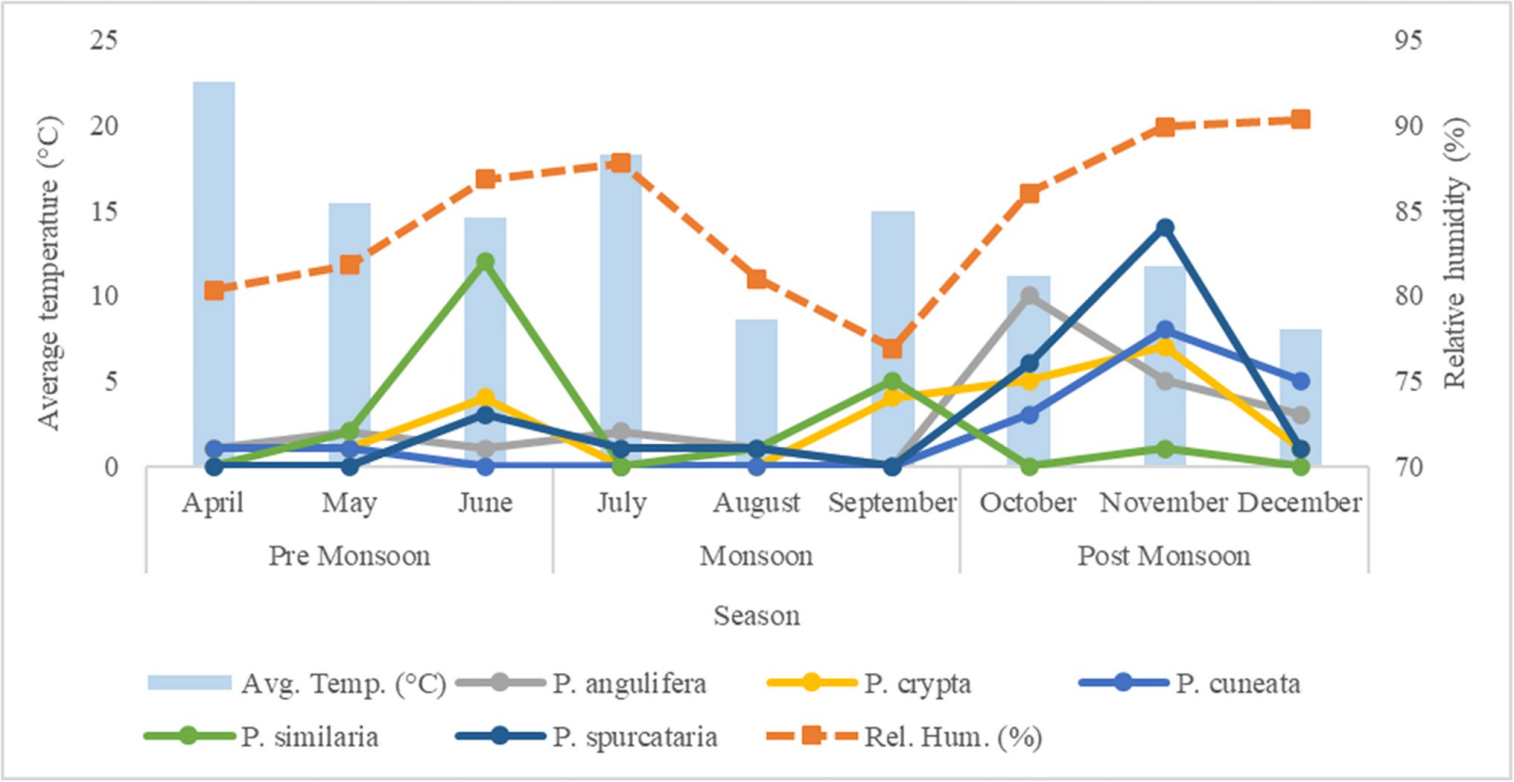
Abundance fluctuations of different *Psyra* species against average trap night temperature and relative humidity in respective months grouped into three broad seasons of Indian Himalaya during sampling period of 2010–2012 and 2016–2019.

Season had a strong influence on *Psyra* activity, i.e., monthly abundance of prominent species varied with fluctuations in average trap night temperature and relative humidity recorded during trap sessions (Fig 21). The general trend indicated that species abundance was high during the pre-monsoon month of June, after which species detection was low during the peak monsoon months of July–August. Abundance again started to rise from September onwards and became maximum during post-monsoon months of October–November. Maximum individual activity was recorded at high relative humidity (86–89%) and moderate (11–14°C) average trap night temperature.

Majority of *Psyra* species had 2–3 activity peaks in a year, though exact number of generations in a year is yet to be documented. The abundance of *P*. *angulifera* remains low all through the year with a population bloom during the months of October to December with a peak during October at 10°C average trap night temperature and 86% relative humidity. *P*. *spurcataria* was active in low numbers during the month of June but became highly abundant during October–November. The seasonal pattern of *P*. *similaria* was unique compared to other species, as its highest abundance was recorded in the pre-monsoon month of June followed by a second peak during September. *P*. *crypta* was moderately active during June and after being almost inactive during the monsoon, individuals started to come to light traps in high numbers during September to November. *P*. *cuneata* was active in very low abundance during pre-monsoon months, becoming negligible in monsoon season after which it became most abundant during October to December.

## Discussion & conclusion

The genus *Psyra*, being primarily originated and evolved in temperate mountainous ecosystems, chiefly within monsoon-influenced Himalayan Biodiversity Hotspot, is distributed throughout its biogeographic provinces and different habitat types. They also colonize the associated hill ranges of North-East India and are equally diverse within Hengduan-Qinghai Mountain ranges and Yunnan-Guizhou Plateau in southern China. Our study reports 12 species/1 subspecies including one new species, *P*. *variabilis* sp. nov. and 4 species/1 subspecies as novel additions to the country, viz. *P*. *gracilis*, *P*. *szetschwana*, *P*. *dsagara*, *P*. *falcipennis* and *P*. *debilis debilis*, known till date from Nepal, China and Pakistan, thus updating the species inventory to 14 species/1 subspecies from India. *P*. *gracilis* and *P*. *szetschwana*, recorded till date only from China, is being reported here from the contiguous landscape of eastern Arunachal Pradesh, thus extending the known range of these species further westward. Among two unreported Indian species in this communication, *P*. *trilineata*, originally described from a single female collected from Darjeeling, is not mentioned anywhere after Hampson [8], and with no recent occurrence record, it can be speculated that Moore described a female *P*. *spurcataria*, with almost identical outer appearance, as *trilineata*, although it cannot be finally concluded without dissecting the holotype currently kept in BMNH. Similarly, *P*. *rufolinearia*, described from a single female from China, and later added to the Indian list from Rothschild’s Darjeeling collection, again from a single female, has not been recently reported anywhere within its known range. With almost identical wing patterns and the type locality being very close to the known distribution range of *P*. *falcipennis*, which was described from Nepal in 1994 and highly abundant throughout Nepal to China, Liu et al. [15] speculated that these two species should be merged in future, with *falcipennis* downgraded as a junior synonym of *rufolinearia*. Another long-standing ambiguity pertaining to the Indian species of *Psyra* centres on the subspecific status and distribution of *P*. *debilis* and *P*. *indica*. The nominotypical subspecies, described from Thundiani in the present Punjab province of Pakistan, was recorded for the first time within the current political boundary of India, almost 130 years after its description, from the Trans-Himalayan cold desert habitat of Ladakh. On the other hand, the subspecies *P*. *debilis indica* was described originally and also reported in this study from much humid Western Himalaya. We described the male genitalia of *P*. *debilis debilis* for the first time here which indicates that the two subspecies share identical male genitalia features, whereas, their outer morphology, especially average wingspan and ecological specificity were quite different. We also found that the two subspecies are non-overlapping in their distribution, with only 1.7% habitat overlap, thus reconfirming their subspecific status. Furthermore, Thundiani as the type locality of *P*. *debilis debilis* is in a quite lower altitude zone receiving significantly higher annual precipitation than the current distribution range of the subspecies only in Trans-Himalayan cold desert, giving rise to the dual probability that either the subspecies already had a range shift towards higher latitude/altitude in the span of last 130 years, or, the type series consisted of *P*. *debilis indica*, both the scenarios are beyond resolving at this point without examining the type specimens of *debilis* or repeat sampling in Thundiani.

The generated barcode sequences of *Psyra* species revealed that overall divergence within the dataset was comparatively low (5.17%), suggesting strong monophyly within the genus. Barcode sequences were successful enough to differentiate between highly cryptic species like *P*. *spurcataria* and *P*. *crypta*, which can only be diagnosed with very minute genitalia differences. As both are novel submissions to Global Barcode Database, we hope that differentiation between these two species would be easier in the future, without the need for genitalia dissection. Homogeneity could not be achieved within the *P*. *similaria* clade, giving rise to the possibility that the specimen collected and sequenced from Dihang Dibang Biosphere Reserve might be a sister taxon, although this cannot be established since morphological and genitalia differences were not sufficient. *P*. *similaria* which is mainly distributed from Western to Central Himalaya, and being documented here for the first time from the eastern extremity of the Himalayas, it seems probable that a sister species/subspecies inhabits the Eastern Himalayan landscape. But at present, due to lack of additional specimens and barcode sequences, we could not describe it as a new taxon. Another relevant aspect in this regard is that the specimen from Dihang Dibang Biosphere Reserve differs from the western population in the arrangement and number of vesica spines, having a circular ridge of small, thin spines instead of two prominent bunch of spines in *P*. *similaria*. These intraspecific differences in the number of spines are also prominent within the populations of *P*. *crypta* and *P*. *debilis*, giving rise to the assumption that the number of vesica spines cannot be a stable character to differentiate between the species. It had also been observed in other families of moth that the number of vesica spines may depend on the size of the individual and males may lose or shed their spines after first mating, and mated males with lost spines had no problem in further mating and ejaculation [51]. Another aspect of notable intraspecific variation exists among widely distributed species like *P*. *angulifera*, *P*. *similaria*, *P*. *crypta*, *P*. *falcipennis and P*. *variabilis*, where, pale to dark morphs appear in accordance with humidity gradient. Thus, drier areas like north-western or western population were dominated by paler individuals, whereas, more humid areas like central and eastern population were dominated by darker individuals.

In typical montane areas, majority of Ennominae genera inhabits closed habitats characterized by high canopy cover and dense undergrowth, as opposed to open habitats [52]. Among 12 species recorded in this study, majority were sampled from closed canopy, humid, evergreen forest types typical of mid-altitudinal zone. Thus, East Himalayan wet temperate forest dominated by broadleaved species of families Lauraceae and Fagaceae like *Acer*, *Michelia*, *Machilus* with undergrowth of *Polygonum*, *Rubus* and many ferns, was home to 9 species including both habitat generalists and specialists. Contiguous with wet temperate zone and partially overlapping with it, the mixed coniferous forests in both western and central-eastern provinces, dominated by *Tsuga*, *Abies*, *Betula*, *Taxus*, *Picea* with dense to low undergrowth of Bamboo, *Rhododendron*, *Rosa* etc., was inhabited by 7–8 species with medium abundance. Forest types immediately below or above this mid-altitudinal broadleaf-conifer zone, characterized by lesser degree of canopy cover like temperate deciduous forest or upper oak-fir forest harboured medium to low species richness and abundance. Trans-Himalayan alpine pastures which are typically open kind of habitat without any tree species and with the ground layer covered by *Caragana-Lonicera-Artemisia* formation with associated patches of *Rhododendron*-*Juniper*, was home to very narrowly specialist species like *P*. *debilis debilis*. Without much information about specific host plant choice of Himalayan *Psyra* species, how plant species composition in these forest types are affecting the diversity of the genus, will be a major focus of future studies. Though a majority of the *Psyra* species were polycyclic, their abundance and richness were at peak during the post-monsoon months of October–November, with another prominent period of activity being reported at the onset of monsoon during June. In the typical Himalayan forests, monsoon favours luxuriant development of tree foliage and undergrowth herbs and shrubs offering wide availability of larval food plants, thus favouring high adult activities in post-monsoon months.

Climate, especially temperature and precipitation in accordance with major topographical feature like elevation govern the distribution of montane species [53]. *Psyra* species composition, abundance and their relation to predictor variables clearly indicated that altitude was the most significant governing factor determining the ensemble structure along with average trap-night temperature and annual precipitation. These findings were bolstered by the distribution modelling which predicted that both precipitation and temperature influence the occurrence probability of the *Psyra* species. Analyzing the overall bioclimatic ranges where the maximum occurrence probability of the species was predicted, we suggest that the species of this genus will be found in areas with moderate mean summer temperature, high winter precipitation in the form of snowfall and mild temperature variability throughout the year. The habitat suitability of the most abundant species *P*. *angulifera* was significantly influenced by overall higher annual precipitation throughout the year, with an evidential influence of winter precipitation. For both *genus Psyra* and *P*. *debilis indica*, comparatively higher precipitation in the coldest/driest quarter seems to be favourable for their distribution. In the Himalayas, the coldest quarter coincides with the driest quarter in the peak winter season when most of the precipitation occurs in the form of snowfall. Higher snowfall during this period ensures water availability throughout the growing season, thus influencing the species distribution via a more direct effect on the availability of their host plants. On the other hand, both temperature of specific quarter of the year as well as their year-long variability governed species distribution. Mean temperature of the wettest quarter in July-August governed the distribution of species in the arid Trans-Himalaya. This is the only growing window in the Trans-Himalaya when the ground layer remains mostly free of snow-cover, giving the herbaceous plant species the opportunity to grow. Suitable temperature during this period in a cold-desert habitat probably favours more growth, thus ensuring availability of both nectar sources for adult moths and fresh foliage for their larvae. The suitable temperature may also trigger over-wintering pupae to emerge when there is availability of food plant for both adults and larvae. Moreover, year-long variation in temperature influences species in more subtle ways. Moderate temperature seasonality favours the overall distribution of the genus, whereas, higher variability characterizes suitable habitats for restricted-range species like *P*. *debilis debilis*, for which incidences of days with extreme temperature may be beneficial. Influenced by moderate temperature in the wettest quarter and high annual temperature variation, this cold-loving high-altitude species was predicted to colonize new areas majorly around its existing habitats in both the future climatic scenarios. Range expansion of Lepidoptera species in response to climate change, though well-recorded among highly migratory butterflies like *Euripus nyctelius* Doubleday (Nymphalidae) [54] or globally known pest species like *Spodoptera frugiperda* (Noctuidae), *Lymantria dispar*, *L*. *monacha* (Erebidae) [55, 56], is rarely documented among very restricted range species. Constrained by their limited trophic breadth, this narrow-tolerant species will accumulate new suitable habitats in response to stochasticity in climatic events. Similar climatic trajectory has been predicted for many range-restricted alpine steppe plant groups like *Taxus wallichiana*, *Rhodiola*, *Roscoea*, *Incarvillea* [57–60], who in contrary to the expectations of the “nowhere to go hypothesis”, would gain additional potential habitat available due to reduction in permanent snow cover with climate warming. Though greater availability of host plants and wider thermal regime would facilitate Trans-Himalayan species, loss of high suitable areas for both *P*. *angulifera* and *P*. *debilis indica* have been predicted from the regions of Western Himalaya and Central Nepal. Moreover, by the end of 2060, under the pessimistic scenario, *P*. *debilis indica* would shrink to highly scattered patches of population. Apart from the climatic constraints, habitat fragmentation might also be one of the causes for the decline in the available species habitats. This kind of species-specific trajectories of future distribution shift among different species of same genus may arise due to differential habitat and micro-climatic tolerances.

Our study provides an overview of updated morpho and molecular taxonomy, ecology, phenology, distribution, habitat suitability and climatic responses of a highly diverse lineage of herbivorous insect group, based on systematically sampled data over a vast biogeographic scale, encompassing entire latitudinal and altitudinal extent of Himalayan Biodiversity Hotspot. Detailed taxonomic treatment supported by identification keys and novel barcode sequences presented here, not only resolved the long-standing ambiguities surrounding highly cryptic sister species complexes, but also indicated the presence of subspecific or even specific level differences between geographically separated populations of *P*. *similaria*, which may be confirmed through further integrated taxonomic approach. Estimating how climate change will impact the habitat distribution of the *Psyra* species can be useful in planning land use management around their existing population, identifying areas where the species may already exist but not yet detected, thus recognizing top-priority survey sites [61]. Moreover, combined taxonomic, ecological and predictive modelling approaches simultaneously under single sampling framework adopted in this study can fulfil the virtual gap created by the absence of historical or long-term data set which hinder the immediate conservation planning of this climatically vulnerable insect species groups.

## Supporting information

S1 Fig Box-whisker plot of altitude, annual mean temperature & annual Precipitation of *P*. *gracilis*, compiled from both primary and secondary data. (TIF)

S2 Fig Box-whisker plot of altitude, annual mean temperature & annual precipitation of *P*. *angulifera*, compiled from both primary and secondary data. (TIF)

S3 Fig Box-whisker plot of altitude, annual mean temperature & annual precipitation of *P*. *cuneata*, compiled from both primary and secondary data. (TIF)

S4 Fig Box-whisker plot of altitude, annual mean temperature & annual precipitation of *P*. *szetschwana*, compiled from both primary and secondary data. (TIF)

S5 Fig Box-whisker plot of altitude, annual mean temperature & annual precipitation of *P*. *fulvaria*, compiled from both primary and secondary data. (TIF)

S6 Fig Box-whisker plot of altitude, annual mean temperature & annual precipitation of *P*. *dsagara*, compiled from both primary and secondary data. (TIF)

S7 Fig Box-whisker plot of altitude, annual mean temperature & annual precipitation of *P*. *similaria*, compiled from both primary and secondary data. (TIF)

S8 Fig Box-whisker plot of altitude, annual mean temperature & annual precipitation of *P*. *debilis debilis*, compiled from both primary and secondary data. (TIF)

S9 Fig Box-whisker plot of altitude, annual mean temperature & annual precipitation of *P*. *debilis indica*, compiled from both primary and secondary data. (TIF)

S10 Fig Box-whisker plot of altitude, annual mean temperature & annual precipitation of *P*. *variabilis*, compiled from both primary and secondary data. (TIF)

S11 Fig Box-whisker plot of altitude, annual mean temperature & annual precipitation of *P*. *falcipennis*, compiled from both primary and secondary data. (TIF)

S12 Fig Box-whisker plot of altitude, annual mean temperature & annual precipitation of *P*. *crypta*, compiled from both primary and secondary data. (TIF)

S13 Fig Box-whisker plot of altitude, annual mean temperature & annual precipitation of *P*. *spurcataria*, compiled from both primary and secondary data. (TIF)

S14 Fig Response curves generated by Maxent for genus *Psyra*. (TIF)

S15 Fig Response curves generated by Maxent for *P*. *angulifera*. (TIF)

S16 Fig Response curves generated by Maxent for *P*. *debilis debilis*. (TIF)

S17 Fig Response curves generated by Maxent for *P*. *debilis indica*. (TIF)

S18 Fig Jackknife of regularised training gain for the modelled *Psyra* species. A. Genus *Psyra* B. *P*. *angulifera* C. *P*. *debilis debilis* D. *P*. *debilis indica*.
(TIF)

S19 Fig Box-whisker plot of altitude, annual mean temperature & annual precipitation of genus *Psyra*, compiled from both primary and secondary data. (TIF)

## Abbreviations

BMNH: The Natural History Museum, London, United Kingdom
DEI: Deutsches Entomologisches Institut, Eberswalde, Germany
NSMT: The National Science Museum (Natural History), Tokyo, Japan
ZFMK: Zoologisches Forschungsmuseum Alexander Koenig, Bonn, Germany
