# Supplementary figures and images for: Taxonomy and ecology of genus Psyra Walker, 1860 (Lepidoptera: Geometridae: Ennominae) from Indian Himalaya
Source: PLoS One. 2022 Apr 13;17(4):e0266100. doi: 10.1371/journal.pone.0266100 (PMC9007390; doi:10.1371/journal.pone.0266100)

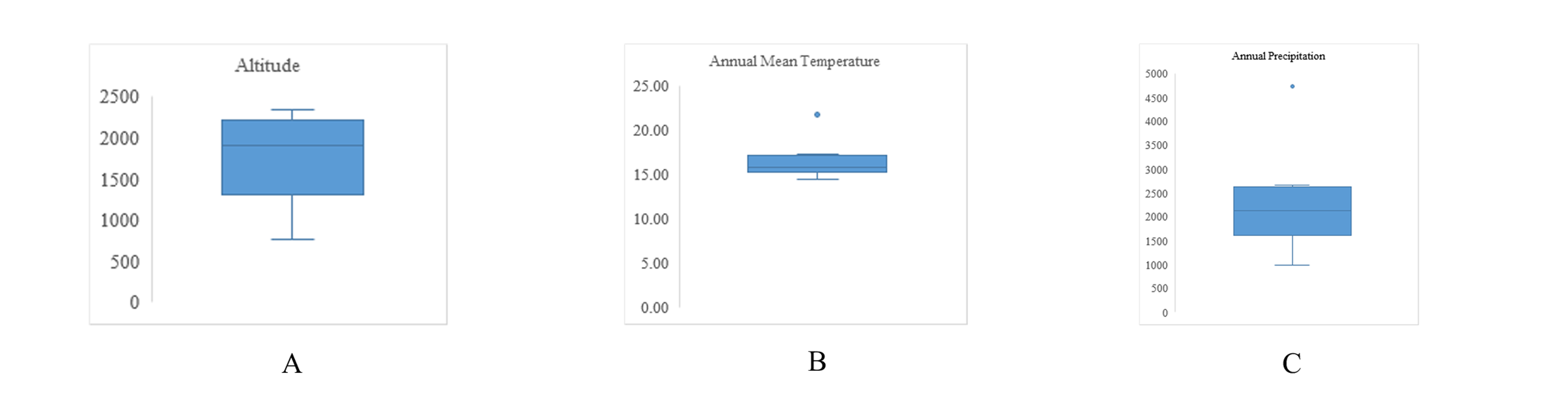

Supplement: S1 Fig — (TIF) [file pone.0266100.s001.tif]

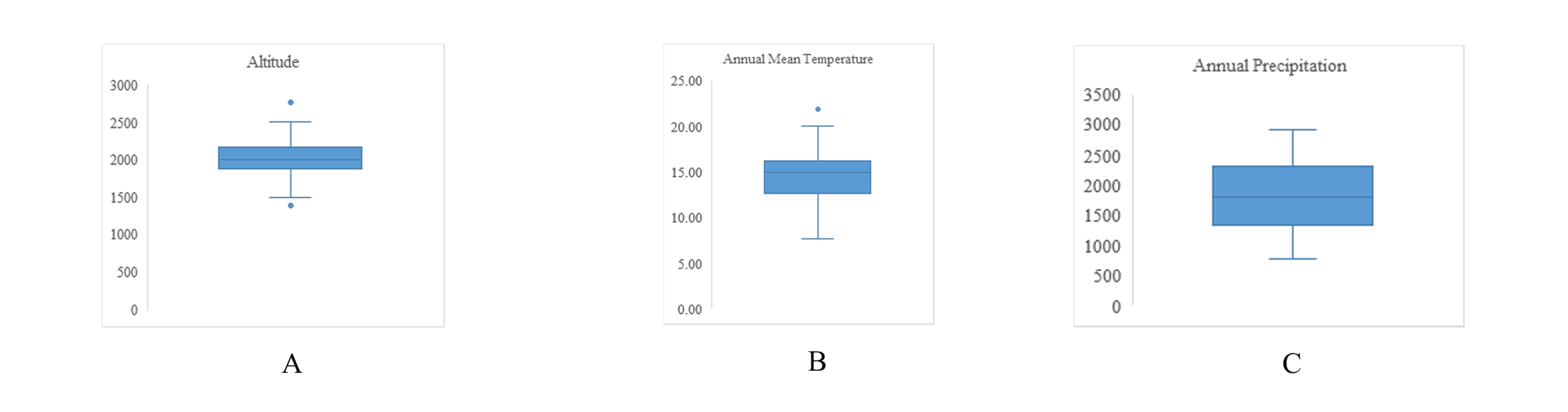

Supplement: S2 Fig — (TIF) [file pone.0266100.s002.tif]

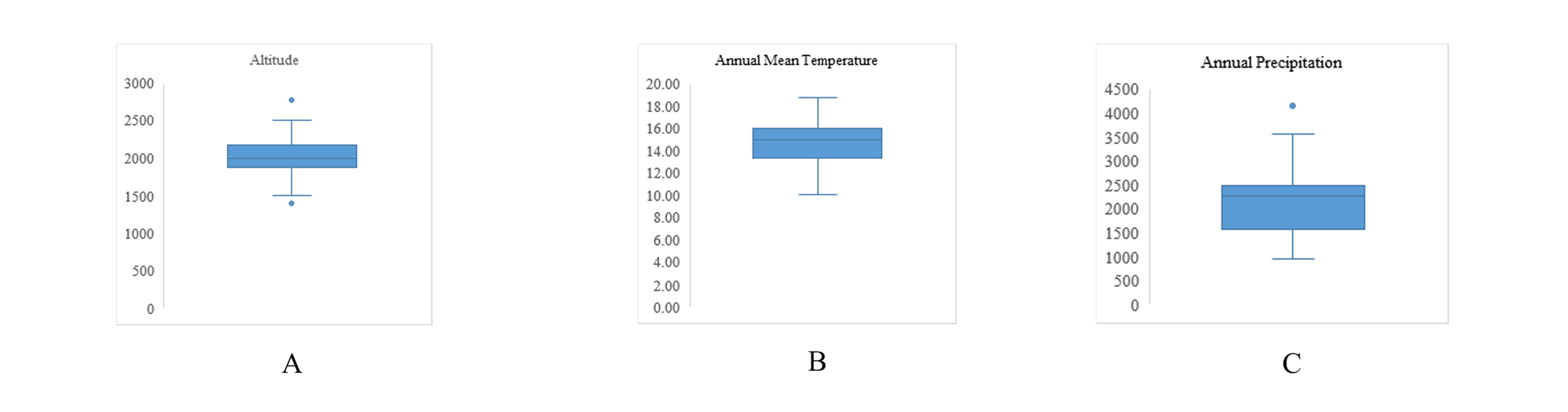

Supplement: S3 Fig — (TIF) [file pone.0266100.s003.tif]

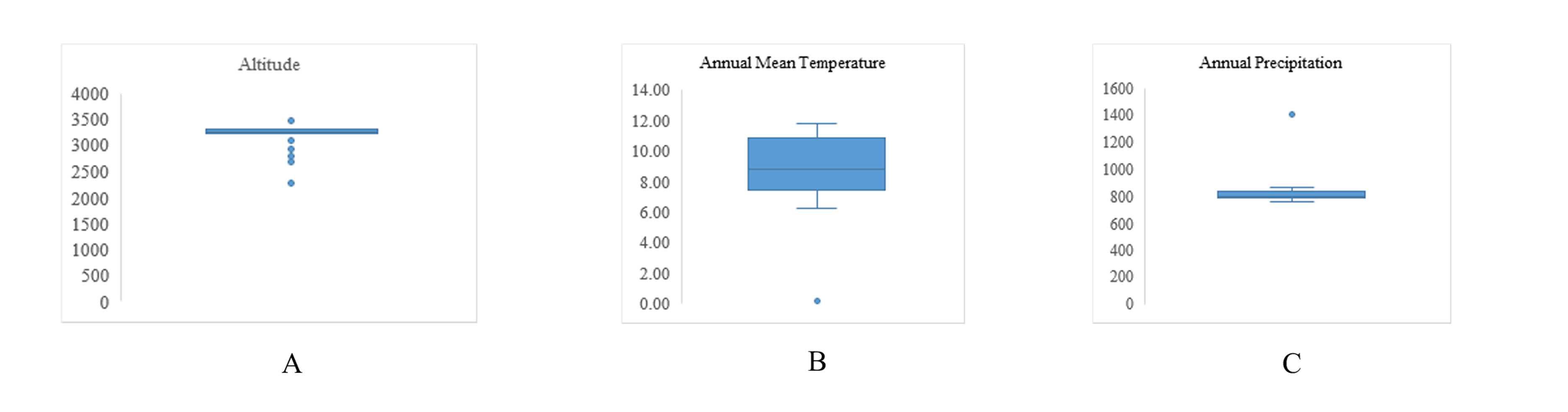

Supplement: S4 Fig — (TIF) [file pone.0266100.s004.tif]

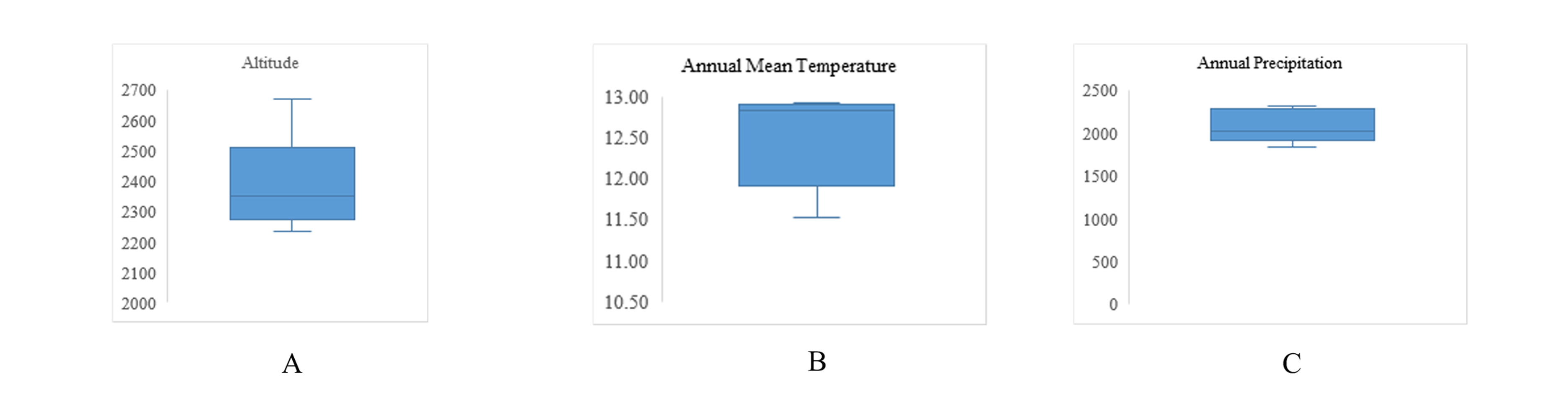

Supplement: S5 Fig — (TIF) [file pone.0266100.s005.tif]

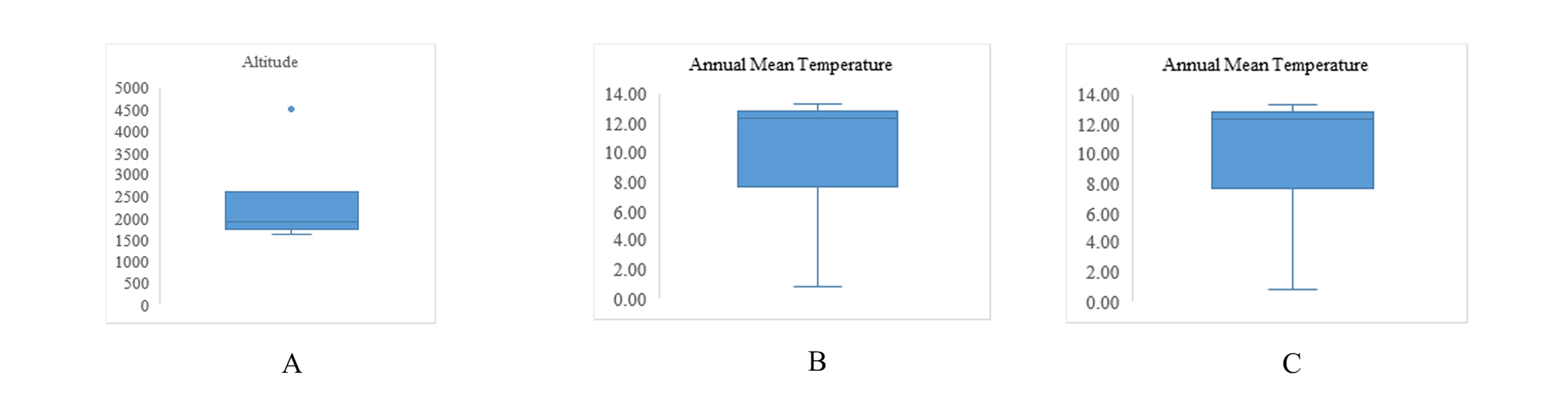

Supplement: S6 Fig — (TIF) [file pone.0266100.s006.tif]

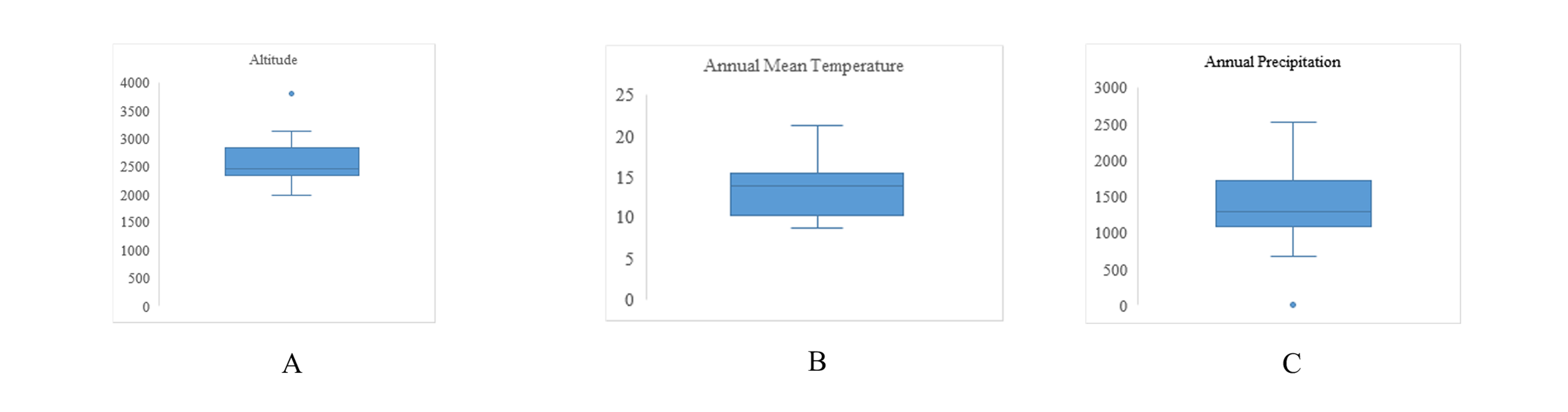

Supplement: S7 Fig — (TIF) [file pone.0266100.s007.tif]

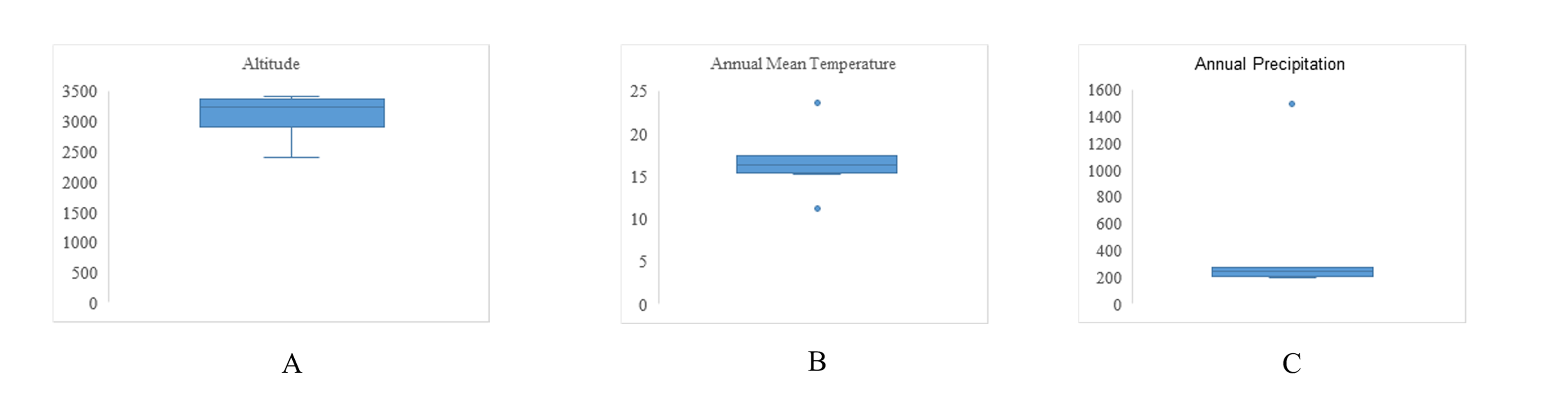

Supplement: S8 Fig — (TIF) [file pone.0266100.s008.tif]

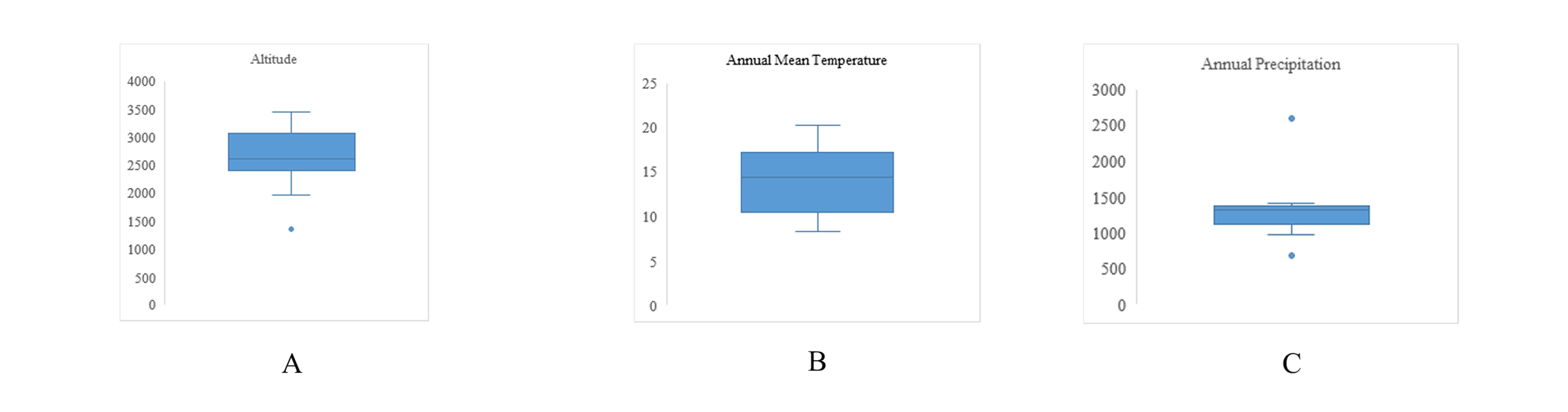

Supplement: S9 Fig — (TIF) [file pone.0266100.s009.tif]

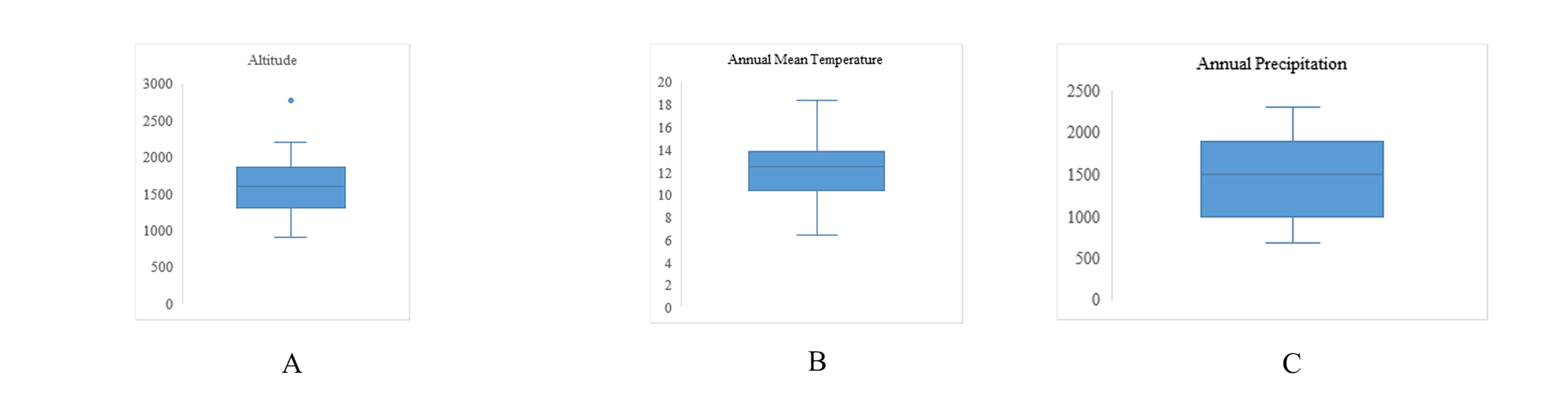

Supplement: S10 Fig — (TIF) [file pone.0266100.s010.tif]

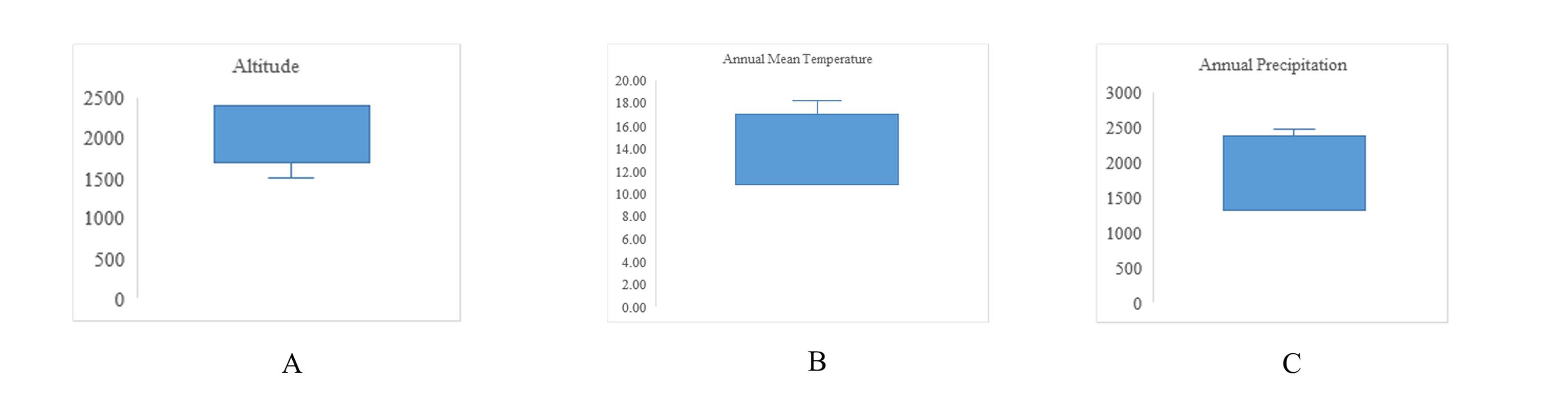

Supplement: S11 Fig — (TIF) [file pone.0266100.s011.tif]

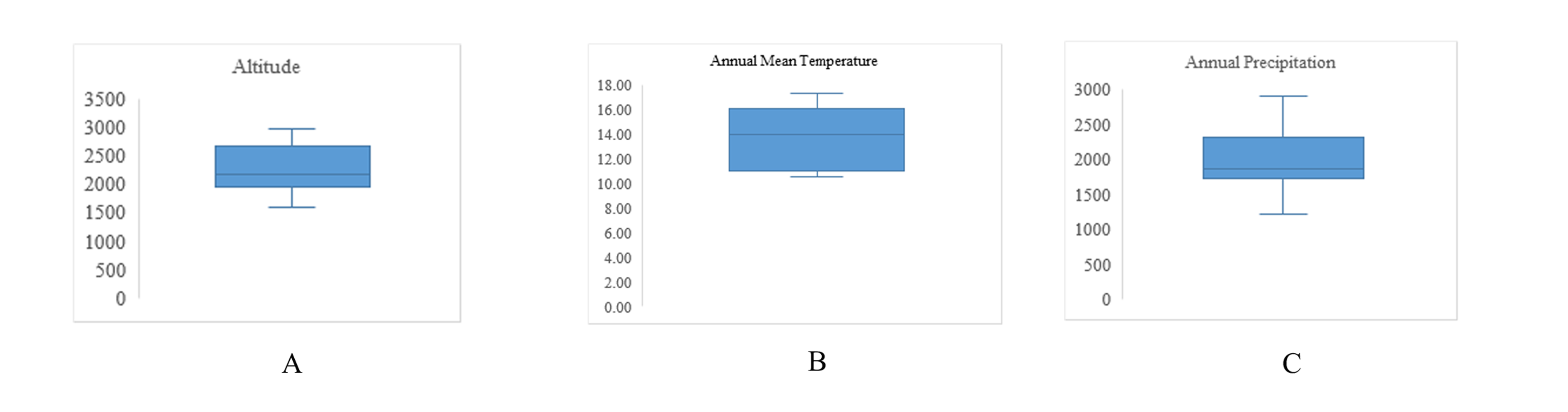

Supplement: S12 Fig — (TIF) [file pone.0266100.s012.tif]

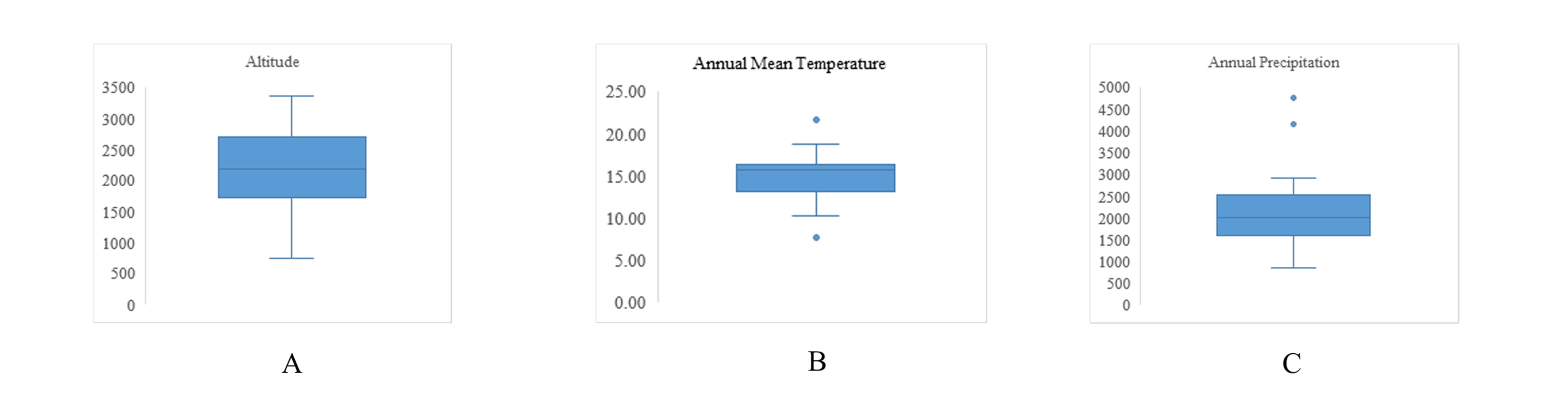

Supplement: S13 Fig — (TIF) [file pone.0266100.s013.tif]

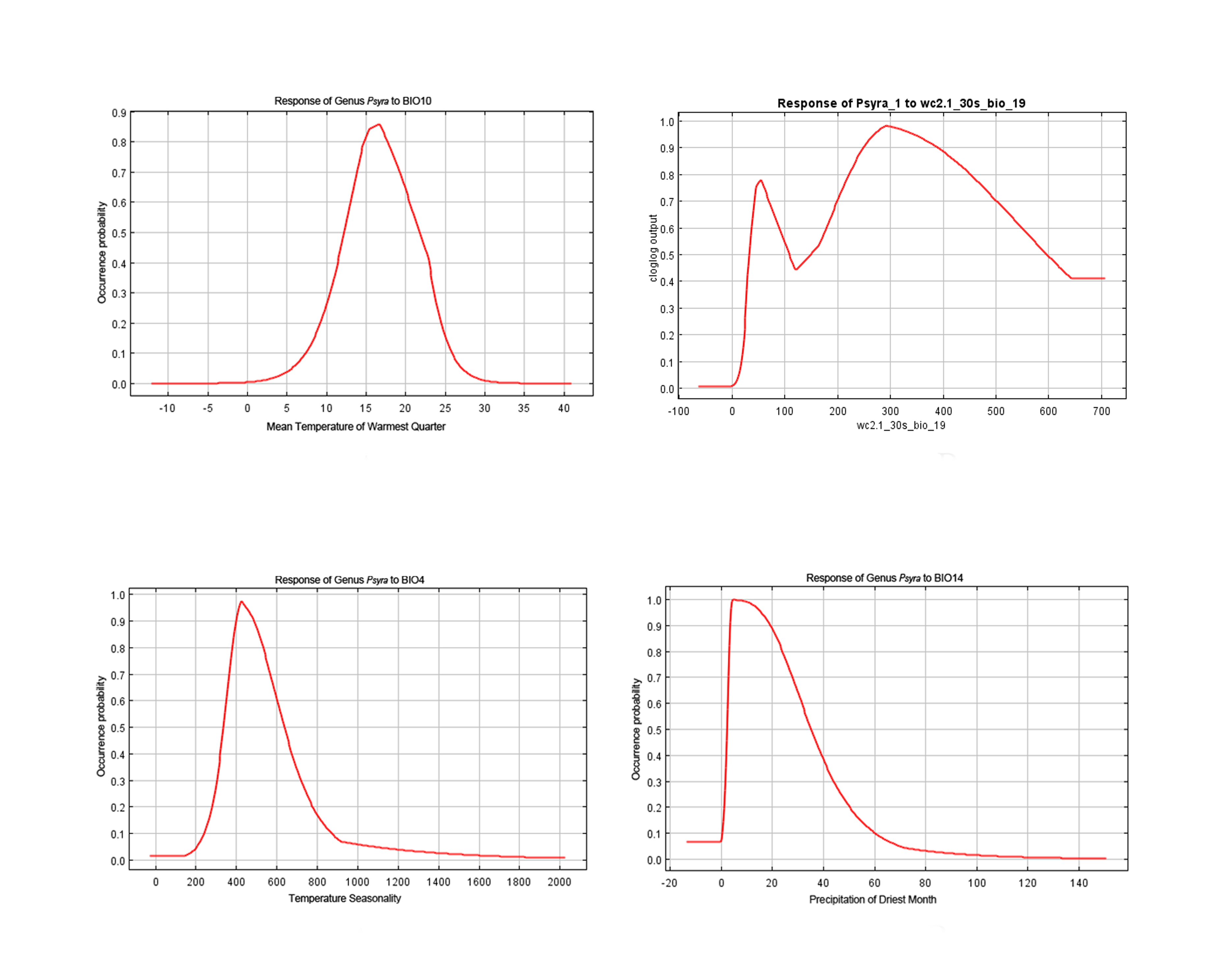

Supplement: S14 Fig — (TIF) [file pone.0266100.s014.tif]

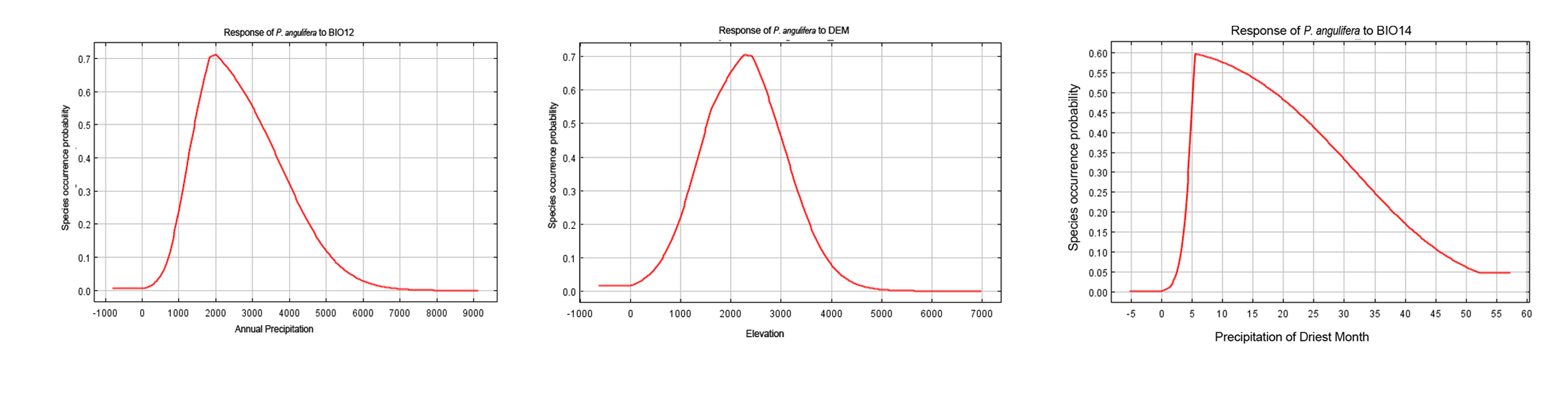

Supplement: S15 Fig — (TIF) [file pone.0266100.s015.tif]

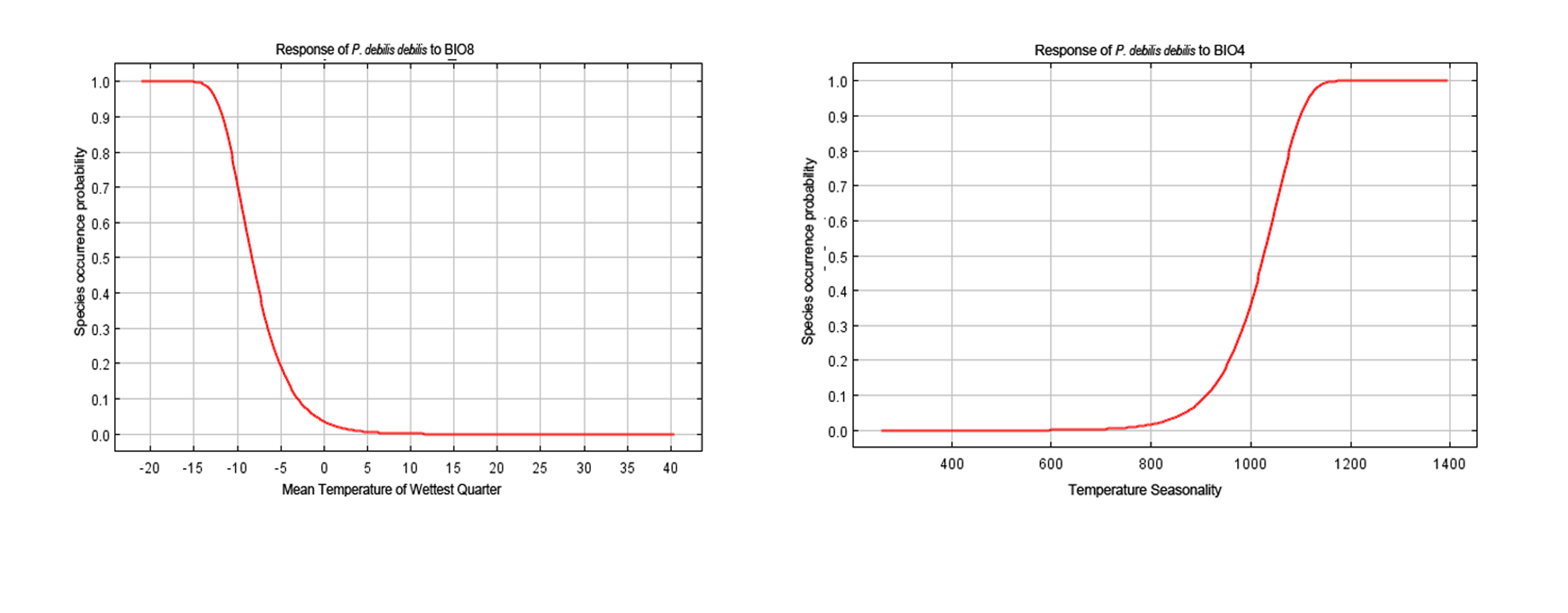

Supplement: S16 Fig — (TIF) [file pone.0266100.s016.tif]

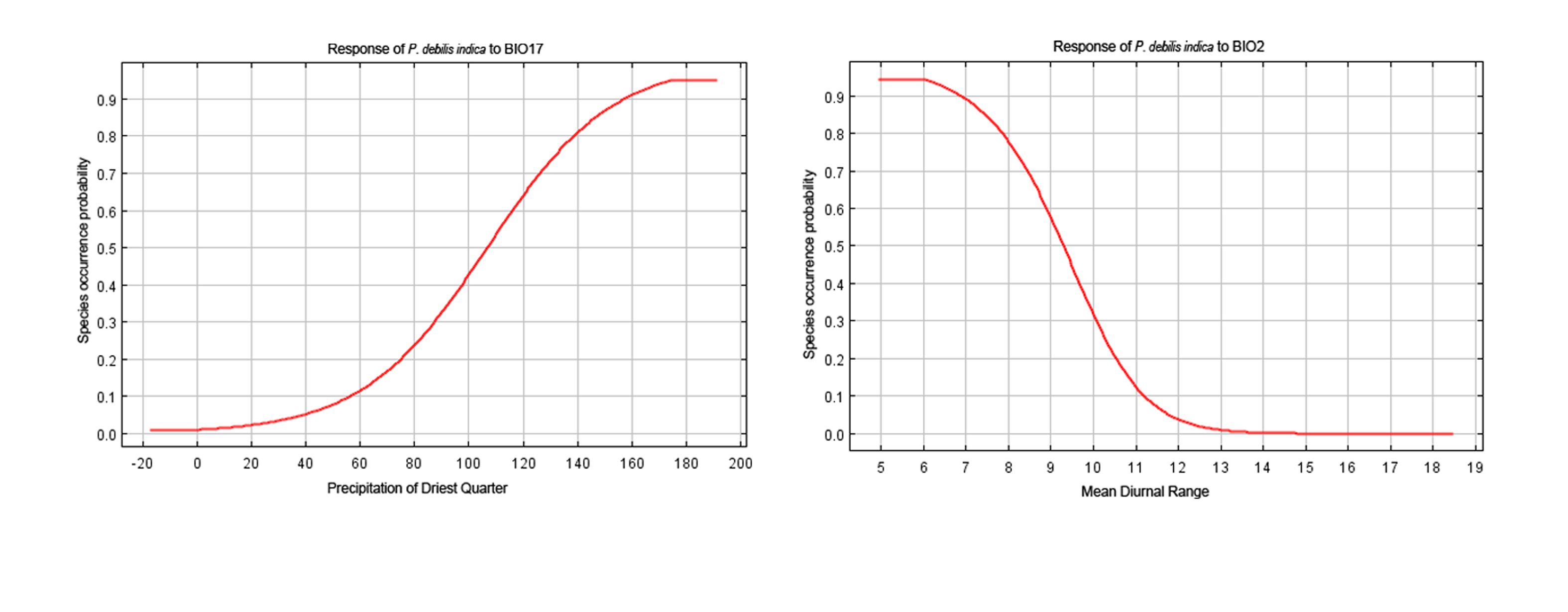

Supplement: S17 Fig — (TIF) [file pone.0266100.s017.tif]

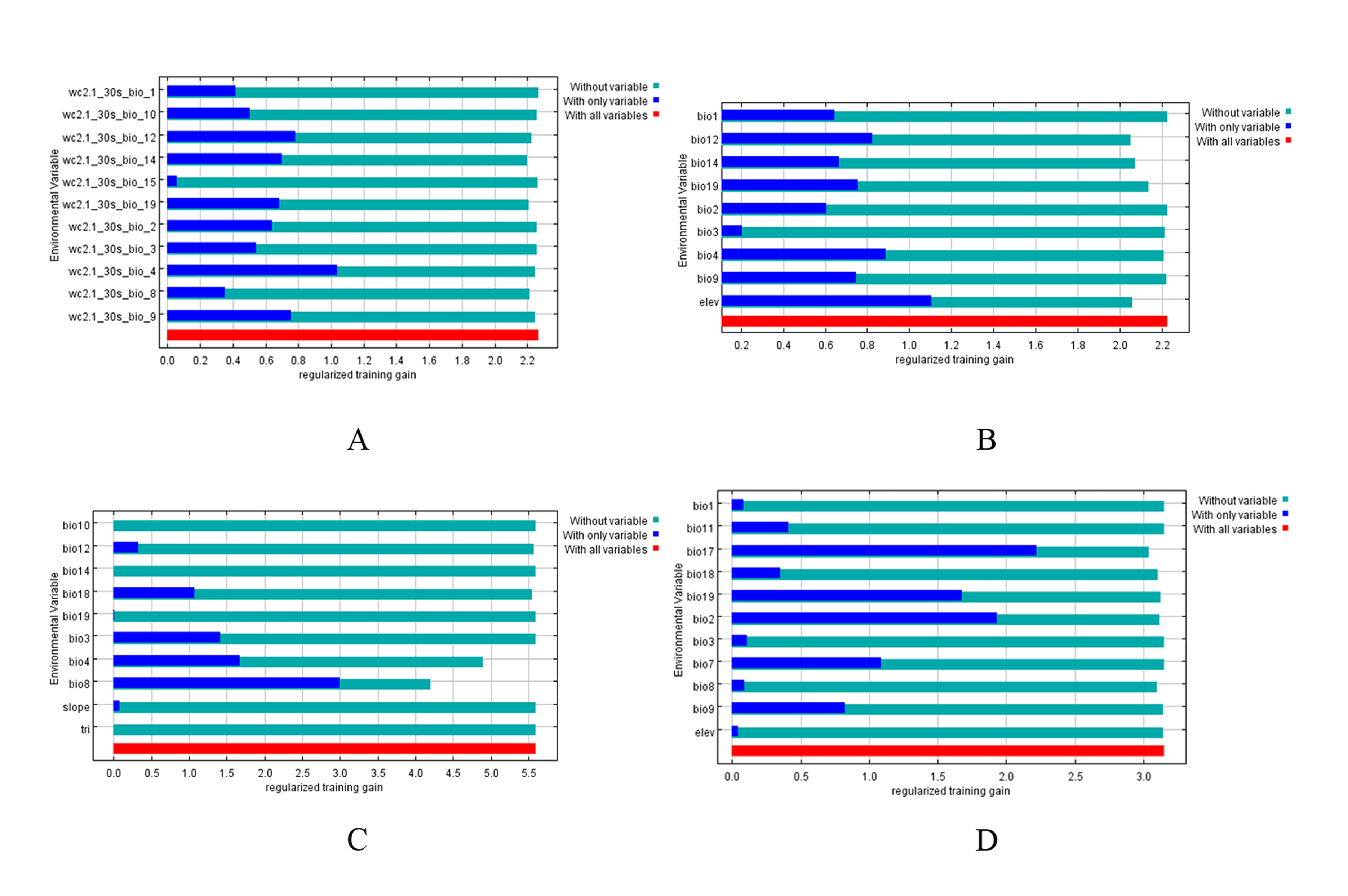

Supplement: S18 Fig — A. Genus Psyra B. P. angulifera C. P. debilis debilis D. P. debilis indica. (TIF) [file pone.0266100.s018.tif]

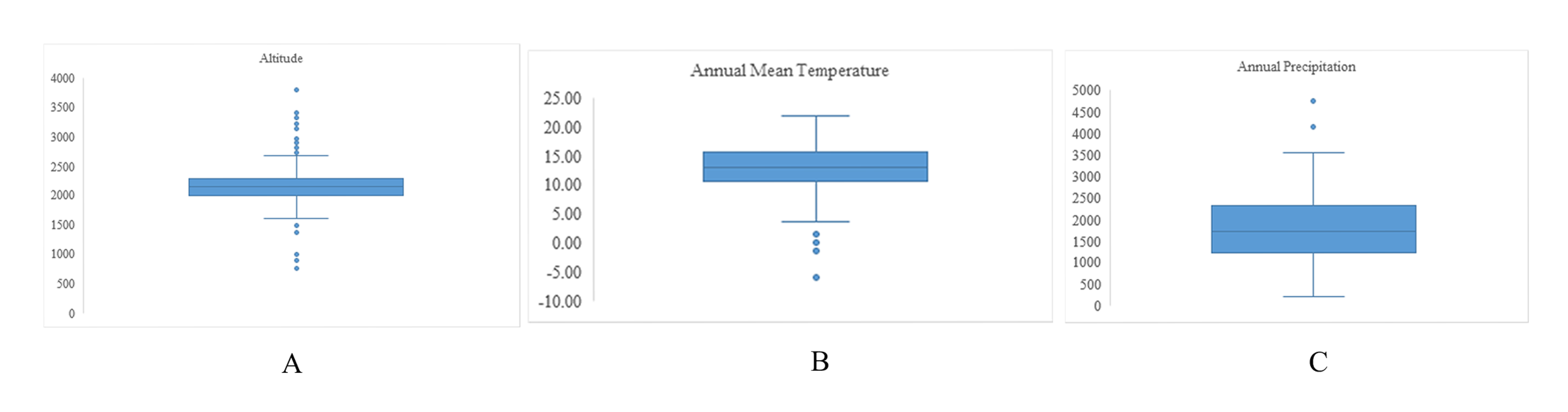

Supplement: S19 Fig — (TIF) [file pone.0266100.s019.tif]
